# Frequently asked questions about chlorophyll fluorescence, the sequel

**DOI:** 10.1007/s11120-016-0318-y

**Published:** 2016-11-04

**Authors:** Hazem M. Kalaji, Gert Schansker, Marian Brestic, Filippo Bussotti, Angeles Calatayud, Lorenzo Ferroni, Vasilij Goltsev, Lucia Guidi, Anjana Jajoo, Pengmin Li, Pasquale Losciale, Vinod K. Mishra, Amarendra N. Misra, Sergio G. Nebauer, Simonetta Pancaldi, Consuelo Penella, Martina Pollastrini, Kancherla Suresh, Eduardo Tambussi, Marcos Yanniccari, Marek Zivcak, Magdalena D. Cetner, Izabela A. Samborska, Alexandrina Stirbet, Katarina Olsovska, Kristyna Kunderlikova, Henry Shelonzek, Szymon Rusinowski, Wojciech Bąba

**Affiliations:** 1 Department of Plant Physiology, Faculty of Agriculture and Biology, Warsaw University of Life Sciences – SGGW, Nowoursynowska 159, 02-776 Warsaw, Poland; 2 Wesemlinstrasse 58, 6006 Lucerne, Switzerland; 3 Department of Plant Physiology, Slovak Agricultural University, Tr. A. Hlinku 2, 949 76 Nitra, Slovak Republic; 4 Department of Agricultural, Food and Environmental Sciences, University of Florence, Piazzale delle Cascine 28, 50144 Florence, Italy; 5 Departamento de Horticultura, Instituto Valenciano de Investigaciones Agrarias, Ctra. Moncada-Náquera Km 4.5., 46113 Moncada, Valencia Spain; 6 Department of Life Sciences and Biotechnology, University of Ferrara, Corso Ercole I d’Este, 32, 44121 Ferrara, Italy; 7 Department of Biophysics and Radiobiology, Faculty of Biology, St. Kliment Ohridski University of Sofia, 8 Dr.Tzankov Blvd., 1164 Sofia, Bulgaria; 8 Department of Agriculture, Food and Environment, Via del Borghetto, 80, 56124 Pisa, Italy; 9 School of Life Sciences, Devi Ahilya University, Indore, M.P. 452 001 India; 10 State Key Laboratory of Crop Stress Biology for Arid Areas, College of Horticulture, Northwest A&F University, Yangling, 712100 Shaanxi China; 11 Consiglio per la ricerca in agricoltura e l’analisi dell’economia agraria [Research Unit for Agriculture in Dry Environments], 70125 Bari, Italy; 12 Department of Biotechnology, Doon (P.G.) College of Agriculture Science, Dehradun, Uttarakhand 248001 India; 13 Centre for Life Sciences, Central University of Jharkhand, Ratu-Lohardaga Road, Ranchi, 835205 India; 14 Departamento de Producción vegetal, Universitat Politècnica de València, Camino de Vera sn., 46022 Valencia, Spain; 15 ICAR – Indian Institute of Oil Palm Research, Pedavegi, West Godavari Dt., Andhra Pradesh 534 450 India; 16 Institute of Plant Physiology, INFIVE (Universidad Nacional de La Plata — Consejo Nacional de Investigaciones Científicas y Técnicas), Diagonal 113 N°495, CC 327, La Plata, Argentina; 17 204 Anne Burras Lane, Newport News, VA 23606 USA; 18 Department of Plant Physiology, Slovak University of Agriculture, A. Hlinku 2, 94976 Nitra, Slovak Republic; 19 Department of Plant Anatomy and Cytology, Faculty of Biology and Environmental Protection, University of Silesia, ul. Jagiellońska 28, 40-032 Katowice, Poland; 20 Institute for Ecology of Industrial Areas, Kossutha 6, 40-844 Katowice, Poland; 21 Department of Plant Ecology, Institute of Botany, Jagiellonian University, Lubicz 46, 31-512 Kraków, Poland

**Keywords:** Chl *a* fluorescence, Delayed fluorescence, Photochemical quenching, Energy partitioning, Area

## Abstract

Using chlorophyll (Chl) *a* fluorescence many aspects of the photosynthetic apparatus can be studied, both in vitro and, noninvasively, in vivo. Complementary techniques can help to interpret changes in the Chl *a* fluorescence kinetics. Kalaji et al. (Photosynth Res 122:121–158, 2014a) addressed several questions about instruments, methods and applications based on Chl *a* fluorescence. Here, additional Chl *a* fluorescence-related topics are discussed again in a question and answer format. Examples are the effect of connectivity on photochemical quenching, the correction of *F*_*V*_/*F*_*M*_ values for PSI fluorescence, the energy partitioning concept, the interpretation of the complementary area, probing the donor side of PSII, the assignment of bands of 77 K fluorescence emission spectra to fluorescence emitters, the relationship between prompt and delayed fluorescence, potential problems when sampling tree canopies, the use of fluorescence parameters in QTL studies, the use of Chl *a* fluorescence in biosensor applications and the application of neural network approaches for the analysis of fluorescence measurements. The answers draw on knowledge from different Chl *a* fluorescence analysis domains, yielding in several cases new insights.

## Introduction

In 2014 we published a paper in question and answer format on a series of chlorophyll (Chl) *a* fluorescence-related topics (Kalaji et al. 2014a). There were, however, still enough questions left for a sequel. In the present paper we treat questions on the relationship between prompt fluorescence (PF), measured with fluorimeters like the PAM and the HandyPEA, and delayed fluorescence (DF), the much weaker cousin of PF that is emitted in response to recombination reactions within PSII; energy partitioning; *q*_P_ versus *q*_L_; the analysis of several forms of stress using Chl *a* fluorescence; the JIP test parameters area and *F*_*J*_; the consequences of fluorescence emitted by PSI for parameters like *F*_*V*_/*F*_*M*_; considerations when sampling trees; the assignment of 77 K fluorescence bands; QTL studies on Chl *a* fluorescence-related traits from a Chl *a* fluorescence point of view and several other topics.

## Question 1: What is chlorophyll *a* fluorescence and why do we study it?

Chl *a* fluorescence can be defined as the red to far-red light emitted by photosynthetic tissues/organisms when illuminated by light of approximately 400–700 nm (photosynthetically active radiation or PAR) (McCree 1972). Within this spectrum, blue and red light excite chlorophyll more efficiently than green light. Although Chl *a* fluorescence represents only a small fraction of the absorbed energy [approximately 0.5–10% (Latimer et al. 1956; Brody and Rabinowitch 1957; Barber et al. 1989; Porcar-Castell et al. 2014)], its intensity is inversely proportional to the fraction of energy used for photosynthesis (a redox effect) (Duysens and Sweers 1963). For this reason, the Chl *a* fluorescence signal can be used as a probe for photosynthetic activity. At the same time, Chl *a* fluorescence is also inversely proportional to changes in dissipative heat emission (a yield effect, i.e., an increase in the yield of heat emission causes a decrease in the yield of fluorescence emission) (e.g., Krause and Weis 1991) and, therefore, Chl *a* fluorescence can be used as well to monitor regulatory processes affecting the PSII antenna (see, e.g., Question 8). Finally, P680^+^ is a strong quencher of Chl *a* fluorescence (Steffen et al. 2005) and this effect allows the study of the different redox states (S states) the oxygen-evolving complex of PSII, due to the fact that the lifetime of P680^+^ is *S* state dependent. All of these things taken together could turn Chl *a* fluorescence into a indecipherable signal, but thanks to the development of specific protocols, and by using complementary techniques, the different effects can be separated, turning Chl *a* fluorescence into a powerful tool for the study of photosynthesis: quenching analysis (Bradbury and Baker 1981; Quick and Horton 1984; Schreiber et al. 1986), JIP test (Strasser and Strasser 1995; Strasser et al. 2004), non-photochemical quenching (NPQ) (Demmig and Winter 1988; Horton and Hague 1988), electron transport rate (ETR) (Genty et al. 1989; Krall and Edwards 1990), rapid light curves (RLCs) (White and Critchley 1999; Ralph and Gademann 2005), flash-induced fluorescence (Robinson and Crofts 1983; de Wijn and van Gorkom 2001; Bouges-Bocquet 1980, Ioannidis et al. 2000), dark-adaptation kinetics of OJIP transients (Bukhov et al. 2001; Schansker et al. 2005), Chl *a* fluorescence and photoacoustic spectroscopy (Buschmann and Koscányi 1989; Snel et al. 1990; Allakhverdiev et al. 1994; Bukhov et al. 1997), Chl *a* fluorescence and 820-nm absorbance/transmission (Klughammer and Schreiber 1994; Schansker et al. 2003), Chl *a* fluorescence and delayed fluorescence (Goltsev et al. 2012; Kalaji et al. 2012a), imaging (Nedbal and Whitmarsh 2004; Hideg and Schreiber 2007; Lichtenthaler et al. 2007; Gorbe and Calatayud 2012), the actinic light wavelength dependence of photosynthesis (Schreiber et al. 2012) and more recently attention has been paid to statistic aspects of the measurements of parameters (e.g., Bussotti et al. 2011a). The photosynthetic literature is huge with many topics studied such as plant breeding (Baker and Rosenqvist 2004; Kalaji and Pietkiewicz 2004; Kalaji and Guo 2008), seed vigor and seed quality assessment (Jalink et al. 1998; Dell’Aquila et al. 2002; Konstantinova et al. 2002), fruit and vegetable quality determination and postharvest processing control (Merz et al. 1996; Nedbal et al. 2000), senescence (Adams et al. 1990a; Kotakis et al. 2014), climate change effects (Ashraf and Harris 2004) and a variety of algae (Gorbunov et al. 1999; Antal et al. 2009; Grouneva et al. 2009). Furthermore, Chl *a* fluorescence measurements have been used for monitoring plant stresses (Guidi and Calatayud 2014), such as photoinhibition (Sarvikas et al. 2010; Matsubara et al. 2011), heat stress (Allakhverdiev et al. 2007; Ducruet et al. 2007; Tóth et al. 2007a; Kalaji et al. 2011a; Brestič et al. 2012), UV stress (Vass et al. 1999; van Rensen et al. 2007; Guidi et al. 2011), salt stress (Kalaji and Pietkiewicz 1993; Demetriou et al. 2007; Melgar et al. 2009; Kalaji et al. 2011b; Penella et al. 2016), drought stress (Lu and Zhang 1998; Flexas et al. 2002; Živčák et al. 2013), urban tree conditions (Hermans et al. 2003; Swoczyna et al. 2010a, b), environmental pollution (Bussotti et al. 2005; Kalaji and Łoboda 2007; Romanowska-Duda et al. 2010; Tuba et al. 2010; Bussotti et al. 2011b; Cotrozzi et al. 2016), sulfur-deprivation/H_2_ production in *Chlamydomonas* (Antal et al. 2007; Nagy et al. 2012) and water quality (Romanowska-Duda et al. 2005; Ralph et al. 2007; Baumann et al. 2009).

## Question 2: Does Chl *a* fluorescence only probe PSII?

A common misunderstanding is that variable Chl *a* fluorescence is a specific probe for PSII. This is true for flash experiments, in which *Q*_*A*_ in all PSII RCs is reduced by a saturating single turnover flash. However, if longer pulses of light are given, *Q*_*A*_ will become reduced and oxidized multiple times, and under these conditions fluorescence also becomes a probe for the reduction and redox state of the PQ pool and even for the electron flow through PSI and PSI content (Schansker et al. 2005; Ceppi et al. 2012).

Under steady-state conditions, i.e., a stable level of photosynthesis reached after a few minutes of illumination, the whole photosynthetic apparatus is in equilibrium and electron flow through any of the components of the electron transport chain (including PSII) would be indicative for the overall photosynthetic rate (Kramer et al. 2004a; Scheibe et al. 2005; Eichelmann et al. 2009). As a consequence, under steady-state conditions, the electron flux calculated on the basis of the Chl *a* fluorescence signal can be used as a measure for the overall photosynthetic activity. This point was demonstrated by Genty et al. (1989, 1990a).

Another common mistake is to interpret fluorescence measurements in terms of single reaction centers. In the case of photoinhibition it is, e.g., often assumed or implied that the quantum yield of individual PSII RCs changes, whereas it is more realistic to interpret changes in the parameter *F*_*V*_/*F*_*M*_ in terms of changes in the quantum yield of the population of PSII RCs as a whole.

The importance of looking at photosynthesis measurements in stochastic terms can be illustrated by experiments showing that at high light intensities 80% of the PSII RCs can be inhibited before the electron transport rate becomes affected (e.g., Heber et al. 1988).

This observation also illustrates that at high light intensities PSII activity has little relevance for photosynthetic activity, whereas at low light intensities PSII RCs become rate limiting. This also means that the effect of a treatment on PSII measured at a single light intensity has limited meaning.

## Question 3: What is the Kautsky effect?

**Fig. 1:**
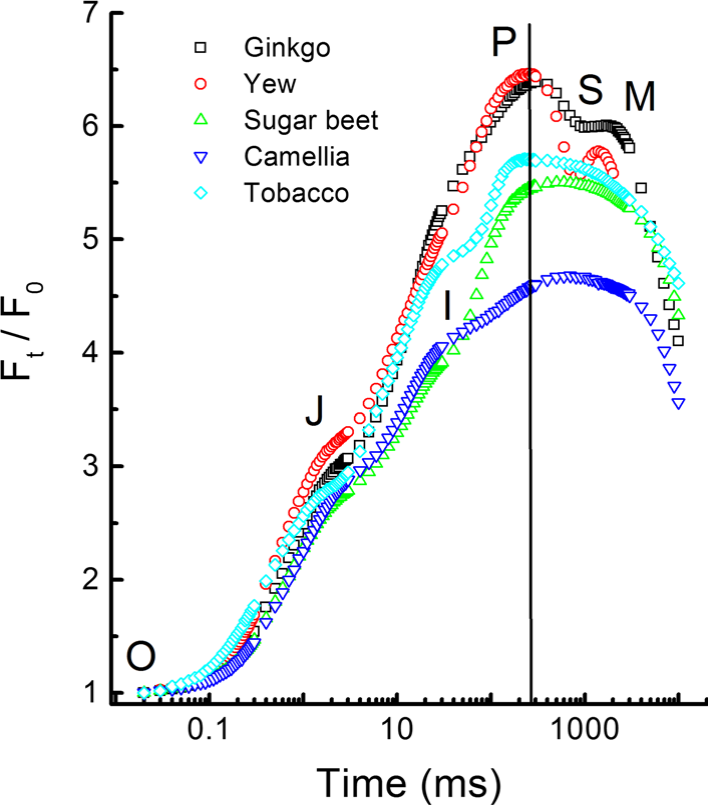
Chl *a* fluorescence induction transients measured on angiosperm (sugar beet, camellia and tobacco) and gymnosperm (*Ginkgo* and yew) leaves. The fast induction kinetics OJIP are similar for both types of plants with a higher *F*_*M*_/*F*_*O*_ ratio in gymnosperms and the same OJIP kinetics for all leaves/needles measured. Beyond *P* the kinetics differ quite strongly between both types of plants (Schansker et al., unpublished data)

Kautsky and Hirsch (1931) observed for several types of leaves that a dark-to-light transition is characterized by an initial fast increase of the fluorescence intensity followed by a slow decrease to a minimum level, after which the fluorescence intensity remains at this low intensity. The authors assigned the stable low level of fluorescence to steady-state photosynthesis. They noted further that the slow fluorescence decrease had the same time dependence as the induction of CO_2_ assimilation and concluded that the fast fluorescence rise reflects a photochemical reaction since it was insensitive to cyanide and temperature changes. The fluorescence changes occurring during induction of photosynthesis have been studied intensively during the last 50 years and, in honor of the first publication on this phenomenon, such a fluorescence transient is called a Kautsky transient, and the changes in the fluorescence intensity the Kautsky effect. In Fig. 1 examples of the first 10 s of Kautsky transients measured on several angiosperm and gymnosperm plants are shown on a logarithmic timescale. The fluorescence rise phase (OJIP) reflects the reduction of the photosynthetic electron transport chain (see Kalaji et al. 2014a for a more comprehensive discussion) and its kinetics, as illustrated in Fig. 1, are quite similar for all photosynthetic organisms. The fluorescence decrease has kinetics that differ quite strongly between different types of photosynthetic organisms (in Fig. 1 angiosperm vs. gymnosperm plants). The *S* and *M* steps observed in transients of gymnosperm species lack/are hidden in transients of angiosperm species. Using 820-nm transmission measurements it was shown that the initial fluorescence kinetics beyond *P* depend strongly on the activation of electron flow at the PSI acceptor side, associated with the activation of ferredoxin-NADP^+^ reductase (FNR) (Kautsky et al. 1960; Munday and Govindjee 1969; Satoh 1981; Harbinson and Hedley 1993; Schansker et al. 2003, 2008; Ilík et al. 2006). Fluorescence then declines within 3–5 min with the onset of photosynthetic CO_2_ fixation until it reaches a lower, steady-state fluorescence intensity (*F*_S_). In fully photosynthetically active leaves this steady-state level, especially at high light intensities, is usually close to the *F*_*O*_ level (e.g., Flexas et al. 2002).

## Question 4: What is quantum yield?

In a general sense, the quantum yield can be defined by an action, e.g., oxygen evolution or a stable charge separation, divided by the number of photons that has to be absorbed for this action. The quantum yield of oxygen evolution has been studied intensively (Warburg and Negelein 1923; Emerson and Lewis 1943; Govindjee 1999). Govindjee et al. (1968) concluded for *Chlorella* cells that the quantum yield for oxygen evolution is at least 0.12, which means that at least 8 light quanta are needed for this process. The maximum quantum yield of a stable charge separation for the dark-adapted state is in the literature defined as *F*_*V*_/*F*_*M*_, and this gives a value of about 0.88 in higher plants (see Question 6). Tyystjärvi and Aro (1996) determined a quantum yield for the photoinhibition of PSII of 7 × 10^−8^, which means that for every 14.3 million photons absorbed, one PSII RC is inactivated. For each photochemical process such a quantum yield can be determined.

If we look at the potential fate of a single photon that has excited a chlorophyll molecule, the sum of the different de-excitation pathways, due to the law of energy conservation, is 1. The three main de-excitation pathways are photochemistry (induction of a stable charge separation), emission as heat, and emission as Chl *a* fluorescence. In open PSII RCs photochemistry is the fastest process and has the highest probability/quantum yield (see also Questions 6 and 13). In more physical terms the quantum yield of photochemistry is the rate constant for photochemistry divided by the sum of the rate constants of all competing processes (photochemistry, heat dissipation, Chl *a* fluorescence emission) [for a more in-depth treatment of this topic: Harbinson and Rosenqvist (2003), Strasser et al. (2004) and Lazár (2016)].

Since photochemistry, fluorescence and heat are competing de-excitation processes, fluorescence measurements can be used to assess the balance between photochemistry and non-photochemical dissipation of absorbed light quanta (photons) under different environmental conditions. It is important to keep in mind that more fluorescence means either less photochemistry and/or less heat (see also Question 13).

## Question 5: When are reaction centers considered to be closed?

The biochemical definition of a closed reaction center is simple. If *Q*_*A*_ is in the reduced state (*Q*_*A*_^−^) no further stable charge separations can occur and the rate constant for photochemistry (kP) goes to 0 (the presence of P680^+^ will also close PSII, but this we will not treat here; see for P680^+^ Questions 18 and 22). Unfortunately, the redox state of *Q*_*A*_ cannot be measured directly under most conditions; therefore, Chl *a* fluorescence is used instead. Based on the paper of Duysens and Sweers (1963) a closed RC is generally equated to *F*_*M*_. However, looking at the literature, the *F*_*M*_ value depends on the technique used to determine it. A single turnover xenon or laser flash is thought to reduce *Q*_*A*_ in all reaction centers. However, the *F*_*M*_ measured under these conditions is 30–50% lower than the *F*_*M*_ induced by a saturating pulse of 200–500 ms (Samson and Bruce 1996). In addition, even at very high light intensities (12,000–15,000 µmol photons m^−2^ s^−1^), where the excitation rate is once every 40–50 µs (Neubauer and Schreiber 1987; Lazár and Pospíšil 1999), which is considerably higher than the re-oxidation time of *Q*_*A*_^−^ of 100–200 µs in the presence of *Q*_*B*_ and 400–600 µs in the presence of *Q*_B_^−^ (Petrouleas and Crofts 2005), it still takes 80–100 ms to reach *F*_*M*_ (Schreiber 1986; Neubauer and Schreiber 1987; Schansker et al. 2006). From a practical point of view, it can be argued that the *F*_*M*_ represents a state with all RCs closed for both single turnover flashes and saturating pulses. The difference is that during a saturating pulse many other things happen as well that affect the fluorescence intensity and, therefore, the *F*_*M*_ values of flash and pulse experiments are not directly comparable.

## Question 6: How can fluorescence measurements and derived fluorescence parameter be corrected for fluorescence emission by PSI?

As noted in the previous paper (Kalaji et al. 2014a), at wavelengths longer than 700 nm PSI fluorescence emission contributes considerably to *F*_*O*_. For commercial fluorimeters this contribution may be as high as 30–35% for C3 plants and 50–60% for C4 plants (Genty et al. 1990b; Adams et al. 1990b; Pfündel 1998; Peterson et al. 2001). The stronger contribution of PSI fluorescence (*F*_PSI_) in C4 plants is due to a higher PSI/PSII ratio (Edwards and Walker 1983; Ku et al. 1991) and to higher levels of spillover of excitation energy from PSII to PSI (Pfündel and Pfeffer 1997). The question whether PSI emits variable fluorescence at room temperature has been studied as well. It is often assumed that the fluorescence yield of open and closed RCs of PSI is the same (Butler 1978; Kyle et al. 1983; Savikhin 2006). Byrdin et al. (2000) reported a 12% increase of the fluorescence yield of PSI of *Synechococcus elongatus* on closing. If *F*_PSI_ is 30% of the *F*_*O*_ fluorescence emission, then 12% more would be equal to 4% of *F*_*O*_, and, since *F*_*M*_ is 5–6 times *F*_*O*_, this would represent 1% or less of the total variable fluorescence. In other words, even if there is some PSI variable fluorescence, this amount is so small that it can be ignored. This is further supported by several kinetic experiments. In leaves or intact chloroplasts, in the presence or absence of DCMU, the *F*_*M*_ is the same (Schreiber and Krieger 1996; Tóth et al. 2005b) despite the fact that in the absence of DCMU P700 is reduced at *F*_*M*_ and in its presence is oxidized (Schansker et al. 2005). In a variation on this experiment Peterson et al. (2014) showed that during fluorescence induction (*F*_*O*_ to *F*_*M*_) the relationship between F(680) (more PSII fluorescence) and F(750) (more PSI fluorescence) did not show an oscillation related to the P700 oxidation and reduction kinetics occurring during OJIP fluorescence rise. Peterson et al. (2014) concluded that variable PSI fluorescence was less than 0.8% of *F*_*V*_. In contrast, theoretical simulations performed by Lazár (2013), based on known values of rate constants of PSI reactions and considering the reported PSII/PSI stoichiometry, yielded an OJIP simulation with approximately correct kinetics. On the basis of these results Lazár concluded that the contribution of PSI variable Chl *a* fluorescence to total *F*_*V*_ could be 8–17%. However, a close link between PSI kinetics and the OJIP rise can also be explained on the basis of the PSII conformational change hypothesis (Schansker et al. 2014).

Several authors have studied methods to correct parameters like *F*_*V*_/*F*_*M*_ for the contribution of PSI fluorescence, but, so far, this has not led to a simple formula that can be applied. It is important to note that the PSI contribution is instrument sensitive. Pfündel (1998) wrote that a special PAM instrument that detects the fluorescence emission at wavelengths shorter than 710 nm shows very little, or at least much less, contribution of PSI fluorescence.

Pfündel (1998) showed for a set of C3, C3–C4 and C4 plants that there is a linear relationship between the parameter *F*_*M*_/*F*_*V*_ determined at room temperature and the parameter F735/F685 determined at 77 K, with a slope *m* and an intercept of the *Y* axis *b*. In the model of Pfündel (1998):

$$ F_{M} /F_{V} = \, b \, + \, m \, \times {\text{ F}}735/{\text{F}}685 $$

For the data set of Pfündel (1998) this gave a regression coefficient of 0.963. On the basis of this approach, a corrected *F*_*V*_/*F*_*M*_ value of about 0.88 was obtained. To use this approach, it would be necessary to construct a calibration curve, like Pfündel (1998) did, for each instrument used and then to determine for the samples of interest both the *F*_*O*_ and *F*_*M*_ at room temperature measured on leaves and the 77 K fluorescence emission spectrum of diluted leaf powder, which in most cases is impractical.

Franck et al. (2002) approached the topic in a different way, developing a method for the resolution of the PSII and PSI contributions to the fluorescence emission spectrum. The authors noted that, for diluted PSII particles, the *F*_*M*_/*F*_*O*_ was wavelength independent. On that basis, they concluded that the wavelength dependence of *F*_*M*_/*F*_*O*_ observed for leaves was due to the presence of PSI. Furthermore, they assumed that the PSI and PSII spectra do not change and, therefore, that these spectra can be scaled to obtain the *F*_*O*_ and *F*_*M*_ spectra. After correction by this method, the authors obtained a *F*_*V*_/*F*_*M*_ value of 0.83 instead of 0.81. This difference is considerably smaller than the correction found by Pfündel (1998).

The quantum yield of PSII can also be determined on the basis of time-resolved (ps) fluorescence measurements. Wientjes et al. (2013a) acclimated *Arabidopsis* plants to 20, 100 and 800 µmol photons m^−2^ s^−1^. Under such conditions the PSII antenna size decreased as the light intensity increased. The quantum yields derived from the time-resolved fluorescence measurements were 0.84, 0.89 and 0.91, respectively. The *F*_*V*_/*F*_*M*_ values (corrected for the PSI contribution) determined for the same plants were 0.83, 0.87 and 0.86, respectively. Since the first set of data is measured on thylakoid membranes and the second set of data on leaves, there are several possible explanations for the observed discrepancies.

The data of Wientjes et al. (2013a) support the choice of a *F*_*V*_/*F*_*M*_ value of 0.87 or 0.88 as a good approximation of the real *F*_*V*_/*F*_*M*_ value, at least for C3 plants. Taking 0.88 as the real value of the parameter *F*_*V*_/*F*_*M*_ of PSII (=Φ_P0_) of C3 and C4 plants, it can be used to estimate the contribution of PSI fluorescence:

1 $$ F_{\text{PSI}} = \left[ {\left( {\phi_{P0} \cdot \frac{{F_{m} }}{{F_{m} - F_{o} }} } \right) - 1} \right] \cdot F_{m} $$

when we take a typical *F*_*V*_/*F*_*M*_ value for C3 plants (e.g., 0.836), we get *F*_PSI_ = ~5.2% of *F*_*M*_. When we take a typical value for C4 plants (e.g., 0.80), we get *F*_PSI_ = ~10% of *F*_*M*_. This calculation can, however, only be applied to *F*_*O*_ and *F*_*M*_ measurements on plants that are completely relaxed with respect to photoprotective dissipation mechanisms (no NPQ) and non-stressed (no photoinhibition). The data of Wientjes et al. (2013a) also suggest that 0.88 is too high for plants acclimated to shade conditions. Another approach will also have to be developed for the correction of the *F*_*V*_/*F*_*M*_ values in the photosynthetic organisms in which the thylakoid stacking is hindered by the presence of phycobilisomes (cyanobacteria, red algae), or the thylakoids are appressed for their entire length (brown algae, diatoms, etc.), or display a not yet well-differentiated grana-intergrana arrangement (most green algae) (see Trissl and Wilhelm 1993; Solymosi 2012). Further, Peterson et al. (2014) described an additional contribution to *F*_*O*_ in greening maize (up to 12–15% of *F*_*M*_ at 680 nm) and sunflower (up to 8% of *F*_*M*_ at 680 nm) leaves which was absent in mature leaves and correcting for which improved the analysis of the fluorescence data. The authors ascribed this fluorescence to emission by partially assembled PSII and could be the same fluorescence emission Srivastava et al. (1999) ascribed to free LHCII. Once *F*_PSI_ has been determined, it can be subtracted from all *F*_*t*_ values and the resulting fluorescence data can be used for the calculation of all fluorescence parameters.

A correction of fluorescence measurements for the PSI contribution is especially relevant when fluorescence measurements are correlated with data obtained by other methods (e.g., gas exchange or absorbance measurements).

Strong red LEDs with a peak emission at ~650 nm were the first LEDs that became commercially available for a reasonable price. Instruments that use such LEDs need to measure fluorescence above 700 nm to avoid overlap with the emission of the red LEDs. This is the case for, e.g., classical PAM instruments and HandyPEAs. Using, e.g., blue LEDs it is possible to avoid the overlap problem and to measure fluorescence emission at ~685 nm, where the contribution of PSI fluorescence is very small (Krause and Weis 1991; Gitelson et al. 1998). However, Peterson et al. (2001, 2014) argued that in the end the fluorescence detected above 700 nm may be the better probe, because light around 680 nm is much more strongly absorbed by the leaf and, therefore, more a probe for the top cell layers of the leaf. Further, it should be noted that differences in filters and other specifications between instruments may affect the contribution of PSI to fluorescence measurements and can explain, at least to some extent, differences in the values of parameters like *F*_*V*_/*F*_*M*_ between different fluorometers.

Part of the JIP test parameters (e.g., M_*o*_, Area, Sm, *V*_*J*_, *V*_*I*_, ψ*E*_*o*_) only depend on variable fluorescence and are not affected by the contribution of PSI fluorescence. For measurements derived from OJIP measurements it may be noted that, as long as the PSII to PSI ratio does not change, PSI fluorescence causes a systematic error. This means that it does not affect the comparability of measurements of comparable samples. With respect to the quenching analysis, the effect of PSI fluorescence emission on the calculated parameters increases for measurements made at stronger actinic light intensities. Higher light intensities quench *F*_*M*_, and to a lesser extent *F*_*O*_, but are not expected to affect *F*_PSI_, increasing the relative contribution of *F*_PSI_. Pfündel et al. (2013) studied the effects of *F*_PSI_ under steady-state conditions. They noted that the method of Oxborough and Baker (1997) to calculate *F*_*O*_′ systematically produces values that are too low and they ascribed this to the fact that Oxborough and Baker (1997) did not take the contribution of *F*_PSI_ into account. Pfündel et al. (2013) also showed that correcting fluorescence data of maize for *F*_PSI_ makes the relationship between Φ_PSI_ and Φ_PSII_ more linear.

In summary, PSI fluorescence emission has only a significant effect on *F*_*O*_. Even a rough correction of fluorescence data for PSI fluorescence emission, assuming that the real *F*_*V*_/*F*_*M*_ value is 0.88, will considerably improve the quality of the fluorescence data.

## Question 7: How does cytochrome *b*_6_/*f* regulate and affect the redox state of the photosynthetic electron transport and parameters like *q*_E_ and ETR?

The cytochrome (cyt) *b*_6_/*f* complex is located between PSII and PSI at a crossroad of different electron pathways (linear electron transport, *Q* cycle, chlororespiration, cyclic electron transport) (Sacksteder et al. 2000; Bennoun 2002; Mulkidjanian 2010; Johnson 2011; Shikanai 2014) and is an important site for the regulation of electron flow and the control of regulatory mechanisms like state transitions (see Question 8) and *q*_E_. The *Q* cycle and cyclic electron transport increase the ATP to NADPH ratio by diverting electrons away from NADP^+^ while at the same time increasing the pH difference over the membrane (Sacksteder et al. 2000; Munekage et al. 2010; Johnson 2011). Lowering the lumen pH decreases the re-oxidation rate of PQH_2_, thereby slowing down electron transport (Witt 1979; Heber et al. 1988; Harbinson et al. 1990). A low lumen pH is also the driving force behind *q*_E_. Kramer et al. (1999), reviewing the literature on the lumen pH, argued that under most conditions the lumen pH remains between pH 5.8 and 6.3. The feedback inhibition of PQH_2_ oxidation may play an important role in keeping the lumen pH within this range. At the same time, this feedback inhibition will lead to a more reduced PQ pool where the cyt *b*_6_/*f* complex is known as a sensor for the PQ pool redox state, activating a kinase that can phosphorylate LHCII when the PQ pool becomes more reduced; this is the classical definition of state transitions (see Question 8).

Under steady-state conditions, a higher light intensity means a more reduced PQ pool and a more oxidized PSI donor side (Klughammer and Schreiber 1994; Živčák et al. 2014). This is due to the fact that PSII can pump electrons faster to the PQ pool and PSI can pump them faster to the electron acceptors at its acceptor side than cyt *b*_6_/*f* can transfer them from the PQ pool to plastocyanin and, then, P700. This imbalance increases as the light intensity is raised and can be detected by measuring Chl *a* fluorescence and 820-nm transmission/absorption simultaneously (Klughammer and Schreiber 1994; Živčák et al. 2014). The described feedback mechanism can respond rapidly to fluctuations in the light intensity and will keep PSI in a relatively oxidized state. The excitation quenching ability of P700^+^ state of PSI has recently been suggested to play a photoprotective role, since in the cyanobacterium *Arthrospira platensis* P700^+^ photostability was shown to reduce PSI photodestruction (Shubin et al. 2008). A similar mechanism was hypothesized to be operative in vascular plants as well (Tikkanen and Aro 2014; Ferroni et al. 2014). A similar observation (sensitivity of PSI to a reduced acceptor side) was made for plants in which cyclic electron transport was inactivated and the plants were exposed to fluctuating light conditions (Suorsa et al. 2012). Tikkanen et al. (2014) have argued that the ability of the chloroplast apparatus to keep PSI in a relatively oxidized state is critical, because damage to PSI is nearly irreversible. It is this aspect that makes the cyt *b*_6_/*f* complex a particularly important regulatory point for electron flow under conditions of changing light conditions (Genty and Harbinson 2004). Tikkanen et al. (2015) recently concluded that the cyt *b*_6_/*f* complex and not downregulation of PSII by the processes related to *q*_E_ regulates linear electron transport. They based this on the observation that PsbS-less *npq4* mutant of *Arabidopsis thaliana* has an impaired ability to generate *q*_E_, but still combines a highly reduced PQ pool with strongly oxidized P700 in high light, indicating that the ability of cyt *b*_6_/*f* to control electron flow is retained. This supports the observation of Belgio et al. (2014) that processes related to *q*_E_ do not reduce the efficiency of PSII to trap excitation energy.

## Question 8: What is a state transition and how does it affect Chl *a* fluorescence?

Bonaventura and Myers (1969) were the first to describe state transitions in cells of *Chlorella* only a few years after the existence of two photosystems had been shown (Duysens et al. 1961). Duysens et al. (1961) had used light of 560 and 680 nm to preferentially excite either of those two photosystems. This was done as well by Bonaventura and Meyers by using so-called light 2 of 645 nm exciting PSII and PSI and light 1 of 710 nm preferentially exciting PSI. The authors observed slow excitation-wavelength-dependent changes in the O_2_ evolution rate and Chl *a* fluorescence, which they interpreted as a change in the distribution of light energy between the two photosystems. In 1977, John Bennett showed that in the light several photosynthetic proteins became phosphorylated (especially LHCII and a 9-kDa protein) and he suggested a link with the above-described state transitions (Bennett 1977). Subsequently, it was shown that a reduced PQ pool was needed to activate the kinase that phosporylated LHCII and that cyt *b*_6_/*f* acted as a redox sensor (Allen et al. 1981; Bennett et al. 1988; Rintamäki et al. 2000).

In the literature, several methods can be found to detect state transitions. A variation of the experimental approach of Bonaventura and Myers (1969) is the determination of the effect of pre-illumination with PSII and PSI light on *F*_*M*_′ induced by a saturating pulse (e.g., Lunde et al. 2000). A variant of this approach is to use the *F*_*S*_/*F*_*M*_′ ratio induced in response to either PSI or PSII light (Wagner et al. 2008). Emission spectra (77 K) are also widely used to detect state transitions. In green algae such as *Chlamydomonas reinhardtii*, in which state 2 is induced by anaerobic conditions that cause a reduction of the PQ pool, this works very well (Depège et al. 2003; Iwai et al. 2008). On going from state 1 to state 2, the PSII bands decrease and the PSI bands increase in amplitude. A variation on this approach is to use the F735/F685 ratio (at 77 K) as a measure for state transitions. This ratio increases during the transition from state 1 to state 2 and decreases during the transition from state 2 to state 1 as, e.g., demonstrated by McCormac et al. (1994) for *Spirodela oligorrhiza*. Studying OJIP transients, Schreiber et al. (1995) observed that state 2 in *C. reinhardtii* and *Synechocystis* PCC 6803 is associated with a reduction in the JI amplitude. Schansker et al. (2006) observed that already at low actinic light intensities the JI amplitude in the steady state decreased, an effect that had still not reversed after 15 min of dark adaptation. The authors interpreted this in analogy with Schreiber et al. (1995) as the effect of a state 1 to state 2 transition. Recently, a simple model to simulate state transitions in *C. reinhardtii* was created (Ebenhöh et al. 2014) based on which Stirbet and Govindjee (2016) tried to simulate the slow PS(M)T fluorescence decline.

Depège et al. 2003 identified the kinase (Stt7) in *C. reinhardtii*, and then, Bellafiore et al. (2005) identified its higher plant ortholog (STN7) in *Arabidopsis thaliani*. However, in the STN7 mutant, which lacked this gene had a phenotype that was very similar to that of the wild type. Only under fluctuating light conditions, the growth of the STN7-less mutant was affected. Grieco et al. (2012) observed that in the STN7 mutant grown under fluctuating white light the PSI content decreases and they proposed, therefore, that state transitions are important in protecting PSI against damage under fluctuating white light conditions.

According to the classical concept of state transitions, the phosphorylated LHCII disconnects from PSII and migrates to PSI, which leads to a redistribution of excitation energy from PSII to PSI (Allen 1992; McCormac et al. 1994; Misra and Biswal 2000). This view has been modified in recent years. Wientjes et al. (2013b) showed that LHCII acts as an efficient antenna for both photosystems under most naturally occurring conditions and that only under special conditions (strong preferential excitation of PSI with FR light or strong light) LHCII migrates to PSII, where, especially under high light conditions, the light it absorbs can be more easily quenched. Grieco et al. (2015) looked at PSII and PSI as located in a connected lake of LHCIIs. For high light conditions, it has been shown that the LHCII kinase becomes inactivated by reduced thioredoxins leading to the dephosphorylated state of LHCII (Rintamäki et al. 2000).

In summary, since its discovery in 1969 the role of state transitions in higher plants has evolved and is still evolving. Based on recent studies on plants lacking the LHCII kinase, state transitions are thought to play a regulatory role under fluctuating light conditions, possibly protecting PSI. Its role in the redistribution of light energy, in higher plants, seems to be less important. For a comprehensive review of this topic, see, e.g., Goldschmidt-Clermont and Bassi (2015).

## Question 9: How can photochemical quenching be defined and what type of information does it provide?

Photochemical quenching is a reflection of the redox state of *Q*_*A*_. If the photosynthetic electron transport chain is oxidized, re-oxidation of *Q*_*A*_ by forward electron transfer can compete strongly with fluorescence emission and can keep the fluorescence intensity low (Kautsky et al. 1960; Munday and Govindjee 1969; Bradbury and Baker 1981; Krause et al. 1982). This is called photochemical quenching (e.g., Bradbury and Baker 1981). If the relationship between *Q*_*A*_ redox state and variable fluorescence were linear, as proposed by Duysens and Sweers (1963), we could simply use the parameter *q*_P_ as it was defined for the quenching analysis (Schreiber et al. 1986, Genty et al. 1989, van Kooten and Snel 1990):

2 $$ q_{\text{P}} = \frac{{F_{m}^{\prime } - F_{s} }}{{F_{m}^{\prime } - F_{o}^{\prime } }} $$

However, as first argued by Joliot and Joliot (1964), this relationship, at least in the presence of a PSII inhibitor like DCMU, is affected by the exchange of excitation energy between the antennae of different PSII RCs. This process has been called connectivity (e.g., Bennett 1983; Dau 1994; Lavergne and Trissl 1995) or grouping (Strasser 1978; Strasser et al. 2004). The parameter *q*_P_ is based on the so-called puddle model, which ignores the connectivity among PSII units. However, if connectivity also affects the whole fluorescence rise in the absence of inhibitors like DCMU, *q*_P_ needs a correction to take this process into account (Kramer et al. 2004b).

The question of how much connectivity affects the fluorescence rise is a contentious one. Strasser and Stirbet (2001) showed, on the basis of a simulation, that in the absence of DCMU connectivity mainly has a measurable effect on the first 300 µs of the fluorescence rise. Beyond that point re-opening and again closing PSII RCs and the effect of the different *S* states on the fluorescence rise (see Questions 15 and 22) disturb the simple relationship that can be derived for DCMU-inhibited PSII RCs. Oja and Laisk (2012) demonstrated only a negligible effect of PSII connectivity and observed that *q*_P_ is proportional to the fraction of open PSII centers in the steady state. Tóth et al. (2007b) showed that the relation between *F*_*J*_ and the area between the OJ rise and *F*_*M*_ as a function of the extent of anaerobiosis was linear, indicating that at the *F*_*J*_ step connectivity no longer affects the fluorescence rise. It has also been suggested that the sigmoidicity of the initial fluorescence rise, which is interpreted to be consequence of connectivity (reviewed by Stirbet 2013) can alternatively be explained on the basis of two overlapping exponential reactions (Vredenberg 2008; Schansker et al. 2011). This can account for the disappearance of the sigmoidicity of the fluorescence rise when the temperature is lowered to −10 °C (Schansker et al. 2011). Schansker et al. (2011, 2014) provided experimental proof that only 70% of the variable fluorescence is related to the redox state of *Q*_*A*_, introducing an additional complication. And finally, there is an important difference between the fluorescence rise in the absence and presence of a PSII inhibitor like DCMU. In the presence of an inhibitor, there is one single charge separation and all *Q*_*A*_ becomes gradually reduced (as a function of the excitation rate). In the absence of an inhibitor, at, e.g., 3000 µmol photons m^−2^ s^−1^, a single excitation of all reaction centers is complete after about 200 µs (cf Neubauer and Schreiber 1987; Lazár and Pospíšil 1999). At longer times, as mentioned above, there is a continuous re-oxidation and re-reduction of *Q*_*A*_ in all RCs (see Questions 16 and 22), where the connectivity effect is likely to average out between all opening and closing PSII RCs.

The experimental data presented in the previous paragraph are not widely known and an approach based on the assumption that connectivity affects the whole fluorescence rise between *F*_*O*_ and *F*_*M*_ (Kramer et al. 2004b) is gaining wider acceptance. Kramer et al. (2004b) derived a modified parameter based on the “lake” model that considers the units to be fully connected, which they called *q*_L_ (Kramer et al. 2004b):

3 $$  q_{{\text{L}}}  = \frac{{F_{m}^{\prime }  - F_{{\text{s}}} }}{{F_{m}^{\prime }  - F_{o}^{\prime } }} \cdot \frac{{F_{o} }}{{F_{{\text{s}}} }}  $$

For the calculation of *q*_L_ without the measurement of *F*_*O*_′, Kasajima et al. (2009) derived the following equation:

4 $$ q_{\rm  L} = {{\left( {\frac{1}{{F_{\text{s}} }} - \frac{1}{{F_{{m}}^{\prime } }}} \right)} \mathord{\left/ {\vphantom {{\left( {\frac{1}{{F_{\text{s}} }} - \frac{1}{{F_{{m}}^{\prime } }}} \right)} {\left( {\frac{1}{{F_{{o}} }} - \frac{1}{{F_{{m}} }}} \right)}}} \right. \kern-0pt} {\left( {\frac{1}{{F_{{o}} }} - \frac{1}{{F_{{m}} }}} \right)}} $$

With the parameters *q*_P_ or *q*_L_, we want to quantify the fraction of open PSII RCs, i.e., the fraction of PSII RCs with *Q*_*A*_ in the oxidized state, in the light-adapted state (Kramer et al. 2004b; Roháček et al. 2008). Depending on the assumptions made (effect of connectivity or not), the value “1 − *q*_P_” or “1 − *q*_L_” represents the approximate redox state of *Q*_*A*_, expressed as *Q*_*A*_^−^/*Q*_*A*_(tot) (Schreiber and Bilger 1987; Weis and Berry 1987). The expression “1 − *q*_P_” represents the balance between excitation rate and forward electron transport and is a reflection of the excitation pressure inside PSII (Ögren and Rosenquist 1992). It is also a measure for the degree of RC closure (Björkman and Demmig-Adams 1995; Roháček and Barták 1999).

*q*_P_ or *q*_L_ values vary between 0 and 1, where 1 is observed in a fully relaxed dark-acclimated state (where *F*_*S*_ = *F*_*O*_) and 0 represents the state when all *Q*_*A*_ is reduced (*Q*_*A*_^−^) and *F*_*S*_ = *F*_*M*_′. See Question 5 for a discussion of the relation between all *Q*_*A*_ reduced and *F*_*M*_.

**Fig. 2:**
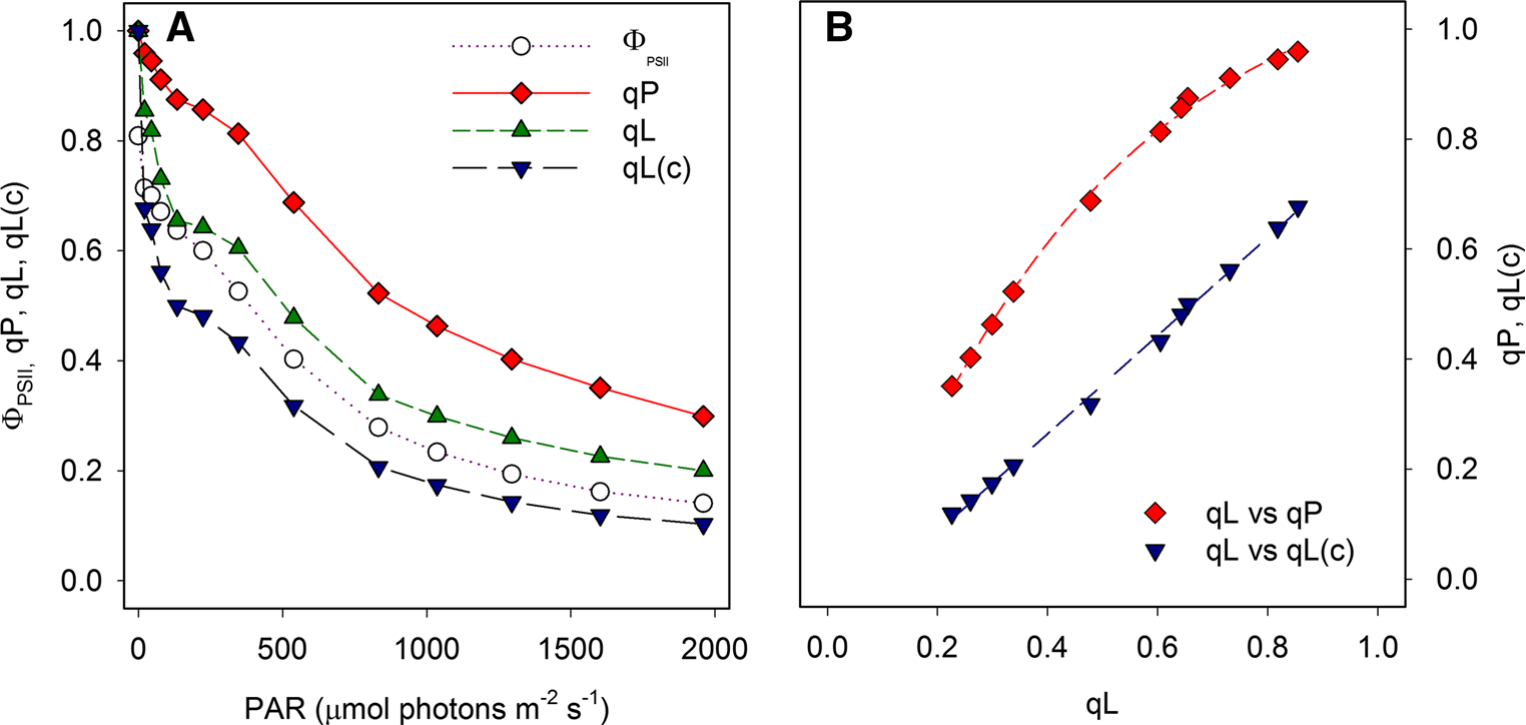
Photochemical quenching. **a** The parameters Φ_PSII_, *q*_P_, *q*_L_ and *q*_L_(c) as a function of the actinic light intensity determined on wheat leaves. **b** The relationship between *q*_P_ and *q*_L_ is nonlinear especially at low light intensities (values close to 1), whereas the relationship between *q*_L_ and *q*_L_(c) is linear with *q*_L_(c) systematically lower than *q*_L_ (Živčák and Brestič, unpublished data)

An example of photochemical quenching (*q*_P_, *q*_L_ and *q*_L_(*c*)) and Φ_PSII_ as a function of the actinic light intensity is presented in Fig. 2a.

Figure 2 demonstrates that there is a considerable difference between *q*_P_ and *q*_L_, as well as between values of *q*_L_ calculated with or without *F*_*O*_′. Figure 2b illustrates that the relationship between *q*_P_ and *q*_L_ is nonlinear. It is worth mentioning that Pfündel et al. (2013) showed that *F*_*O*_′ values estimated on the basis of the method of Oxborough and Baker (1997) are systematically too low (see Question 6).

The choice between *q*_P_ and *q*_L_ depends on the way we look at the Chl *a* fluorescence induced by a saturating pulse. If we treat it as essentially a single charge separation, an analogy with the fluorescence rise in the presence of DCMU can be inferred, as Kramer et al. (2004b) did. If we take into account that the fluorescence rise induced by a saturating pulse consists of many turnovers of *Q*_*A*_, the analogy is lost and then the straightforward parameter *q*_P_, though far from perfect, is probably a much better approximation of the *Q*_*A*_ redox state in the light then the parameter *q*_L_.

## Question 10: Is the electron transport rate (ETR) calculated from Chl *a* fluorescence a reliable parameter?

The electron transport rate (ETR) estimated from Chl *a* fluorescence is often defined as:

5 $$ {\text{ETR}} = \phi_{\text{PSII}} \cdot {\text{PPFD}} \cdot 0.5 \cdot {\text{leaf}}\;{\text{absorptivity}}\; {\text{coefficient}}\; \left( {{\mu\text{mol}}\;{\text{electrons}}\;{\text{m}}^{ - 2} \;{\text{s}}^{ - 1} } \right) $$

where Φ_PSII_ (which is dimensionless) is the effective quantum yield of photosystem II in the light; PPFD (µmol photons m^−2^ s^−1^) is the photosynthetic photon flux density incident on the leaf (or any green organ); leaf absorptivity coefficient (which is dimensionless) is the absorptance of the photosynthetic organ, i.e., the proportion of the incident PPFD effectively absorbed by the photosynthetic surface, and “0.5” is a correction factor for PPFD, assuming that half of the photons are absorbed by PSI and the other half by PSII as first formulated by Krall and Edwards (1992). As formula 5 shows, ETR and Φ_PSII_ are proportional and are, therefore, closely related parameters.

The parameter ETR has been shown to correlate well with linear electron flow calculated on the basis of O_2_ evolution rates (Flexas et al. 1999; von Caemmerer 2000). Genty et al. (1989) observed a linear correlation between Φ_PSII_ and CO_2_ assimilation rate at 1% O_2_ for barley and 20% O_2_ for mays. Edwards and Baker (1993) extended the number of conditions under which a linear correlation was observed. However, in many other studies a nonlinear, somewhat concave, relationship was observed (Peterson et al. 2001 and references therein). Correcting for PSI fluorescence improves the linearity of the relationship (Peterson et al. 2001).

For the leaf absorptivity coefficient, 0.85 is a typical value for C3 plants (Ehleringer and Pearcy 1983; Krall and Edwards 1992; Schultz 1996).

The value “0.5” is a rough estimate. Von Caemmerer (2000) wrote that this factor varies between 0.45 and 0.5. In contrast, in some studies (e.g., Strasser and Butler 1977) it was observed that PSII absorbs more light than PSI. As a first approximation, and in the absence of further information, “0.5” is likely the best choice.

Peterson and Havir (2003, 2004) considered the possibility that the rate constants kN of heat dissipation and/or kF of Chl *a* fluorescence change during the OJIP fluorescence rise. According to Dau (1994) the relationship between 1/*F*_*O*_ and 1/*F*_*M*_ should be linear and proportional (slope = 1) if NPQ is purely due to dissipation of excitation energy in the antenna. Peterson and Havir (2003) tested this assumption for WT and psbS mutants of *Arabidopsis thaliana*. They observed that for WT leaves the relationship is biphasic with a steeper slope at low light intensities than at high light intensities. In the case of the psbS-mutant leaves there is a strong deviation from linearity, mainly because the mutant shows no *F*_*O*_ quenching. Peterson and Havir (2004) extended this study to 10 *A. thaliana* lines and concluded that during an OJIP rise (saturating pulse) the kN and/or kF changes. They showed that this affects the relationship between ETR based on fluorescence measurements and ETR based on gas exchange measurements. They further showed that the relationship between these two parameters could be improved considering the redox state of *Q*_*A*_. Schansker et al. (2011, 2014) proposed that during the OJIP rise the fluorescence yield increases (kF increases) depending on the time *Q*_*A*_ remains reduced, before becoming re-oxidized by forward electron transport. This interpretation model supports the observations and interpretations by Peterson and Havir (2003, 2004).

There are several other factors that may affect the correction factor 0.5: (1) state transitions (see Question 8) can cause a redistribution of light between PSII and PSI on a minutes timescale, especially in algae (Bonaventura and Myers 1969; Depège et al. 2003; Iwai et al. 2008); (2) the extent of stacking and the associated changes in spillover, again especially in algae (see Trissl and Wilhelm 1993 for a discussion of this point), may have a considerable effect on the distribution of light between the two photosystems; (3) as shown by Anderson et al. (1988), there are at least threefold differences in the PSII/PSI ratio (ranging from at least 1:1 to 1:3) between different plant species. This range may in part be compensated by differences in PSII antenna size, but it is likely that it also represents some variability in this parameter; (4) long-term acclimation of a plant species to different light regimes affects PSII antenna size and PSII/PSI ratio (Bailey et al. 2001; Ballottari et al. 2007; Hogewoning et al. 2012; Bielczynski et al. 2016). A change in both the PSII antenna size and the PSII/PSI ratio affects the Chl *a*/*b* ratio and may be used as an indicator for effects related to points (3) and (4).

Not only variations in the value “0.5,” but also corrections of Φ_PSII_ should be considered. As noted in Question 6, a correction of Φ_PSII_ for PSI fluorescence emission will yield a more reliable ETR value, especially for C4 plants, and improve the linear correlation with CO_2_ assimilation measurements (Pfündel et al. 2013).

In Kalaji et al. (2014a) simultaneous Chl *a* fluorescence and CO_2_ assimilation measurements, and the information such measurements can yield, are discussed. In that paper the problems of using Ф_PSII_ or ETR as indicators for the quantum yield of CO_2_ assimilation by the leaf ($\varPhi_{{{\text{CO}}_{2} }}$) were also discussed extensively. By determining the linearity of the relationship between ETR and CO_2_ assimilation (in the case of C3 plants in the presence of 2% O_2_ to suppress photorespiration), the usefulness of ETR as a measure for CO_2_ assimilation can be established for individual cases.

**Fig. 3:**
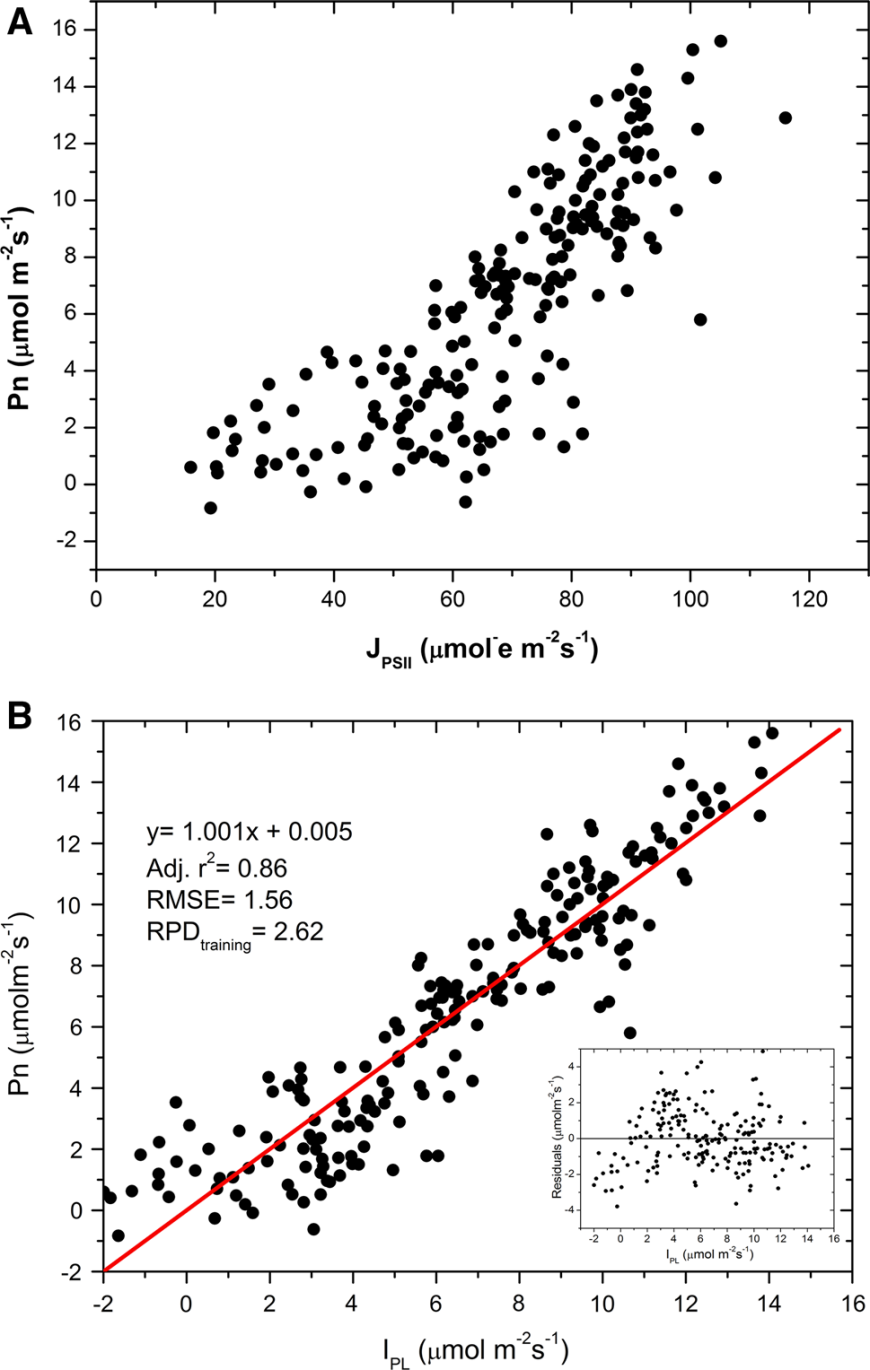
Factors affecting the relationship between the electron transport rate (ETR) and net photosynthesis (*P*_n_) in apple. **a** *P*_n_ as a function of ETR for 21 apple (C3 plant) genotypes exposed to different drought stress conditions. The relation is nonlinear; **b** *P*_n_ as a function of a parameter (*I*_PL_) based on ETR, leaf temperature and the leaf-to-air temperature difference and derived by a multivariate approach. This yielded a strongly linearly correlated relationship with a slope of nearly 1. (Figures **a** and **b** are adapted from Figs. 1d and 3 in Losciale et al. 2015)

In C3 species where the linearity between ETR and net CO_2_ assimilation is often absent due to the existence of alternative electron sinks, especially photorespiration (see Question 11), a multivariate approach was shown to be a good alternative (Losciale et al. 2015). The rationale of this method is: to consider the main factors affecting net photosynthesis; to identify the related variables, and to combine these variables using a multivariate semi-mechanistic approach. Roughly, net photosynthesis (*P*_n_) is a function of: (1) the electron transport rate of the ETC; (2) the CO_2_ concentration at the carboxylative sites, which depends on stomatal and mesophyll conductance; and (3) the carboxylative activity of Rubisco, which depends on the Michaelis–Menten constants for carboxylation, Kc, and photorespiration, Ko. The first factor can easily be determined using Chl *a* fluorescence (ETR), and the last two are strictly related to the leaf-to-air temperature difference (Δ*T*) and the leaf temperature (Tl), used for Ko and Kc estimation (von Caemmerer 2000). Using the function

6 $$ P_{\text{n}} = \alpha + \beta_{1} \left[ {{\text{ETR}}\left( {\frac{\text{Ko}}{\text{Kc}}} \right)} \right] + \beta_{2} \left( {\Delta T} \right) $$

it was possible to estimate accurately net photosynthesis based on the measurements of ETR, leaf and air temperature, only. The parameters *α*, *β*_1_ and *β*_2_ are species-specific, and the model has been parameterized and validated for apple and pear (Losciale et al. 2015). As illustrated in Fig. 3, the calculated *P*_n_, called *I*_PL_ in Fig. 3b, shows a better linear relation with *P*_n_ than *J*_PSII_ (Fig. 3a).

In summary, ETR has been shown to linearly correlate with CO_2_ assimilation under several conditions. CO_2_ assimilation is, of course, only one of the available electron sinks (see Question 11), photorespiration in C3 plants being an important alternative sink, and, therefore, it should not be used to estimate absolute rates of CO_2_ assimilation (Baker 2008). Correcting the ETR calculation for PSI fluorescence emission (Question 6), taking into account possible deviations of the factor from 0.5 as well as the inclusion of several easy to measure parameters as described in the previous paragraph may further improve the usefulness and reliability of this parameter.

## Question 11: What are the experimental differences between Chl *a* fluorescence and gas exchange measurements?

ETR (“Electron Transport Rate”) is the fluorescence parameter that gives a measure for the linear transport of electrons from H_2_O (i.e., from PSII) to the Calvin–Benson cycle (or other sinks, see below) under steady-state conditions. If ETR and the CO_2_ assimilation rate are compared, several theoretical and experimental factors have to be considered.

### Electron sinks

It is important to realize that CO_2_ assimilation is only one of several potential electron sinks. The most important competitor under stress conditions, that cause reduced stomatal opening, is photorespiration. Photorespiration is mainly an issue for C3 plants (Cornic and Fresneau 2002). The most important alternative pathway under high light conditions is also photorespiration (Foyer and Noctor 2009; Bauwe et al. 2010). Photorespiratory activity can be nearly completely suppressed if the oxygen concentration is reduced to 2% or less. In C4 plants photorespiration is negligible (Laisk and Edwards 1998). Other electron sinks are the Mehler reaction (i.e., the reduction of molecular oxygen on the acceptor side of PSI) (Asada 1999; Foyer and Noctor 2009), cyclic electron transport around PSI (e.g., Heber and Walker 1992), nitrogen and sulfur metabolism, which also consume ATP and NADPH (e.g., Neyra and Hageman 1974; Leustek et al. 2000; Kopriva and Rennenberg 2004) and the export of reducing equivalents to mitochondria or peroxisomes (Raghavendra and Padmasree 2003; Yoshida et al. 2007). The importance of these alternative sinks under steady-state conditions has been a discussion issue for many, many years (see, e.g., Peterson and Havir 2004) and is beyond the scope of the present review.

### Structural considerations

The assimilation rate of CO_2_ reflects the photosynthetic activity of the whole leaf, whereas ETR measurements derive mainly from fluorescence emission by chloroplasts in the top cell layers (in most cases, mainly the palisade parenchyma cells) of the leaf. In addition, *P*_n_ is measured by infrared gas analyzers (IRGAs) on an entire leaf or, more commonly, a significant part of it; Chl *a* fluorescence, in contrast, is measured on a much more limited area of the leaf (a few square millimeters) (Rosenqvist and van Kooten 2003). Thus, the chloroplasts placed near the adaxial side of the leaf may be photoinhibited (e.g., by strong light or low temperatures in combination with moderate light), whereas other chloroplasts (deeper in the leaf) are photosynthesizing normally. However, given the steep light gradient inside the leaf, these chloroplasts will receive much less light.

In summary, IRGA and Chl *a* fluorescence refer to different spatial scales in terms of surface and depth. Discrepancies between ETR and CO_2_ assimilation can, at least in part, be explained on the basis of these differences.

Finally, coexistence of mitochondrial respiration and photosynthetic metabolism needs to be considered. Photosynthesis measured by IRGA represents the net exchange of CO_2_—gross photosynthesis minus the CO_2_ produced by respiration and photorespiration. In general, the respiration rate of a leaf is low compared with photosynthesis (around 5–10%) (e.g., Kromer 1995) and can therefore be disregarded. However, in leaves with very low photosynthetic rates (e.g., plants under severe environmental stress or deep-shade-adapted species), respiration becomes comparatively more important.

If a linear relationship between ETR and CO_2_ assimilation is observed, one can conclude either that the contribution of alternative sinks is negligible, or that their contribution is light intensity independent. The same is true for the structural effects. A systematic study of the effects of these factors on the relationship between ETR and CO_2_ assimilation would, therefore, be useful. It is, however, always good to keep in mind the factors mentioned above that may affect the relationship between fluorescence and IRGA measurements.

## Question 12: Is it meaningful to determine energy partitioning?

Demmig-Adams et al. (1996) published a paper in which they observed that the parameters *q*_*N*_ and NPQ only gave relative values for energy dissipation as heat. They wanted to find a way to quantify in absolute terms the fraction of energy dissipated as heat. The authors noted that in sun leaves non-photochemical quenching was high and the PSII reaction centers remained relatively open. In shade leaves little non-photochemical quenching was induced and the PSII reaction centers were to a large extent closed. The authors then concluded that in shade leaves there is little heat dissipation. This conclusion, however, is questionable.

Let’s have a look at a dark-adapted leaf. If a saturating pulse is given to a dark-adapted leaf, all PSII reaction centers become closed, photochemistry is reduced to 0, and excitation energy can only be dissipated as either heat or fluorescence emission (if we ignore connectivity, spillover and oxidative damage). For such a saturating pulse it is also assumed that it does not induce non-photochemical quenching. Under such conditions fluorescence emission has been determined to be ~10% (Barber et al. 1989). This means that at least 90% of the excitation energy in closed PSII reaction centers is dissipated as heat. This happens both in sun and in shade leaves. The induction of *q*_E_ can increase this percentage by no more than 10% by completely outcompeting fluorescence emission. In other words, the induction of the processes associated with *q*_E_ has only a rather small effect on the fraction of excitation energy dissipated as heat. However, the purpose of the processes associated with *q*_E_ is not so much to increase the fraction of heat dissipation; its purpose is to reduce the lifetime of excitation energy in the antenna. It may be expected that the probability that excitation energy will cause oxidative damage is a function of the lifetime of an exciton.

It can, therefore, be argued that the idea of Demmig-Adams et al. (1996) was based on a false premise. Dissipation of excitation energy as heat in closed PSII reaction centers is always 90% or higher. What changes is an increase of the efficiency with which the antenna can dissipate excitation energy as heat if processes associated with *q*_E_ are induced. A shift in the lifetime of excitation energy from 1.6–1.8 to 0.5 ns as violaxanthin is turned into zeaxanthin (Z) + antheraxanthin (A) has been observed by Gilmore et al. (1998). These authors observed that changes in the parameter *F*_*M*_′/*F*_*M*_ can be used to monitor directly fluorescence lifetimes, intrathylakoid pH and [Z + A].

Recently, Lazár (2015) reviewed all the efforts that have been made to improve on the original concept of Demmig-Adams et al. (1996). Demmig-Adams et al. argued that in addition to photosynthesis and dissipation there is an undefined component, called “excess” by the authors. Since no process is associated with this component this is difficult to accept and understand (see Lazár 2015 for a discussion of this point). Another point of criticism of the approach of Demmig-Adams et al. can be that their approach is too simplistic and ignores other processes that affect Chl *a* fluorescence. Kornyeyev and Hendrickson (2007) and Kornyeyev et al. (2013) included photoinhibition and other photochemical and non-photochemical processes in their analysis. However, the observation that only ~10% of the absorbed energy, which in dark-adapted leaves is emitted as fluorescence, can be quenched and emitted as heat is also valid for all these improvements.

In summary, fluorescence, heat dissipation and photosynthesis compete with each other. In a closed PSII RC photosynthesis is 0 and only fluorescence and heat dissipation compete. If the rate constant for heat dissipation (kN) increases, heat dissipation will increase at the cost of fluorescence emission, which is observed as fluorescence quenching. The rate constant kN increases if the lifetime of the associated process decreases as observed by Gilmore et al. (1998) for the xanthophyll cycle.

## Question 13: What is the fate of light absorbed by PSII?

Once light is absorbed by the PSII antenna, it is efficiently transferred to the PSII RCs and if they are in the open state the most likely fate of the excitation energy (~90%) is that it is used to drive a charge separation between the accessory chlorophyll Chl_D1_ and Pheo followed by a transfer to *Q*_*A*_ (see Question 6). Long and Humphries (1994) reported on the basis of the literature that in full sunlight the percentage of absorbed light used for the photosynthetic process as a whole drops to 5–10%. This observation was confirmed by Losciale et al. (2010).

Alternative de-excitation pathways are: (1) dissipation as heat by the carotenoids bound to the antenna proteins (Gibasiewicz et al. 2005; Bode et al. 2009); (2) fluorescence emission by Chl *a* molecules (Butler 1978; Lazár 1999; Maxwell and Johnson 2000; Kalaji et al. 2012b); (3) in the case of photoinhibition of a part of the PSII RCs, transfer of excitation energy from active to inactive RCs (see below); (4) the excitation of oxygen, resulting in singlet oxygen (reviewed in Krieger-Liszkay et al. 2008). These processes are all in competition. If *Q*_*A*_ is reduced, the pathway related to charge separation is blocked, the lifetime of the excitation energy increases, and the other de-excitation pathways become more important.

The rate constant for dissipation as heat is increased considerably by photoprotective quenching, associated with ∆pH, PsbS and the xanthophyll cycle (Ort 2001). The xanthophyll cycle (i.e., the formation of zeaxanthin) can reduce fluorescence emission by 75–90% (Demmig-Adams et al. 1996; Flexas and Medrano 2002), which means that nearly all absorbed energy is dissipated as heat (see also Question 12).

Pogson et al. (1998) found that in addition to the xanthophyll cycle pigments (zeaxanthin and antheraxanthin), the α-carotene-derived xanthophyll lutein, which is a structural component of the subunits of the light-harvesting complexes, contributes to the dissipation of excess absorbed light energy and the protection of plants against photooxidative damage. Lutein is also thought to contribute to q_E_ (Pogson et al. 1998; Müller et al. 2001). The xanthophyll neoxanthin, on the other hand, that also binds to antenna proteins, does not seem to play a role in energy dissipation (reviewed by Jahns and Holzwarth 2012).

A special case, in this respect, is formed by photoinactivated PSII RCs. When inactivated, PSII RCs are disassembled and repaired (Aro et al. 1993). During a clear sunny day, in the absence of any additional stress, the total PSII pool can be completely inactivated and repaired without photoinhibition being detectable (Chow and Aro 2005). If plants also suffer from other stresses like water or nitrogen deficiency (Jifon and Syvertsen 2003; Cheng et al. 2000), a significant population of inactivated PSII RCs may accumulate in the thylakoid stacks. It has been proposed that these RCs play a role in the dissipation of light energy under high light conditions (Matsubara and Chow 2004; Chow et al. 2012). Matsubara and Chow (2004) showed that photoinhibition of PSII enriched membranes, which do not show xanthophyll cycle activity, caused the induction of a 0.3-ns lifetime component at the expense of 1.7- and 3.9-ns components. This suggests that photoinhibited PSII RCs become strong quenchers of light energy. On this basis, Matsubara and Chow (2004) proposed that by connectivity with other active PSII RCs the photoinhibited PSII RCs can act as quenching sinks. In thylakoid membranes isolated from photoinhibited leaves the authors observed 1.25- and 0.58-ns lifetime components, which they associated with photoinhibition. It may be noted, however, that the assignment of the 0.58-ns lifetime component to photoinhibition can be challenged, since Gilmore et al. (1998) ascribed a similar 0.5-ns component to the effect of zeaxanthin formation.

The formation of singlet oxygen by excited Chl molecules is a multistep process. The excited singlet Chl state (^1^Chl) can return to the ground state under the emission of fluorescence. Alternatively, a transition of the ^1^Chl state to the triplet state (^3^Chl) can occur with a certain probability. There are two sources of ^3^Chl: the PSII antenna and ^3^P680 following recombination (Krieger-Liszkay 2004). ^3^Chl can return to the ground state under transfer of its energy to O_2_. This leads to the formation of very reactive singlet O_2_ (^1^O_2_). The recombination pathway depends on the midpoint potential of *Q*_*A*_^−^. Fufezan et al. (2002) showed that in the presence of the phenolic herbicide bromoxynil the midpoint potential of *Q*_*A*_^−^ is lowered, charge recombination between Pheo^−^ and P680^+^ is favoured, and the ^1^O_2_ yield is higher. This indicates that it is this recombination pathway that leads in the RC to singlet oxygen formation via the ^3^P680 state. Singlet O_2_ is thought to play a role in photoinhibition (see Krieger-Liszkay et al. 2008 for a discussion of this point).

The excitation pressure is not only reduced by more efficient heat dissipation but also by a higher electron transport rate (see Question 11). As discussed in Question 8, preventing a strongly reduced acceptor side of PSI is important. Tiwari et al. (2016) showed recently for the pgr5 mutant, in which cyclic electron transport around PSI is inhibited, that the FeS clusters on the acceptor side of PSI become more easily damaged under high light conditions, showing the importance of this process for the protection of PSI. The glutathione–ascorbate cycle is also thought to play a role in the protection of the acceptor side of PSI from damage under conditions of low Calvin–Benson cycle activity (Noctor and Foyer 1998; Baker and Rosenqvist 2004). Baker and Rosenqvist (2004) noted that the particular physiological effects of different stresses determine which alternative process becomes more active.

Regulation of thermal and photochemical de-excitation pathways, together with the PSII recovery system, all contribute to a photoprotective system, which prevents photodamage to the photosynthetic apparatus (Baker and Rosenqvist 2004). Photoinhibition and photoprotective mechanisms can be detected using several techniques, but the most useful method is the measurement of Chl *a* fluorescence (Krause and Weis 1991; Govindjee 1995; Maxwell and Johnson 2000; Losciale et al. 2008, 2010).

## Question 14: How to probe the donor side of PSII?

Oxygen evolution takes place at the donor side of PSII, which consists of a Mn cluster of four manganese ions, a Cl^−^ and Ca^2+^ ion and the surrounding protein environment (Debus 1992; Yocum 2008). Access of reductants like ascorbate is limited by the presence of three extrinsic proteins of the oxygen-evolving complex (OEC), i.e., PsbO, PsbP and PsbQ (reviewed by Tóth et al. 2013). The electron transfer link between the Mn cluster and P680 is a tyrosine molecule located in the D1 protein called TyrZ. During the oxygen-evolving process, the PSII donor side passes through five redox states, *S*0 to *S*4, of which the *S*4 state is not stable and passes to the *S*0 state within about 2 ms under the release of an oxygen molecule. Flash-induced oxygen evolution (Kok et al. 1970; Joliot et al. 1971), flash-induced fluorescence (Bouges-Bocquet 1980) and flash-induced DF (Grabolle and Dau 2005) are characterized by period-4 oscillations. For the analysis of the *S* states, the five-state model as introduced by Kok et al. (1970) is still largely valid: The OEC following dark adaptation is in the *S*1 state, and the period-4 oscillations are dampened by misses and double hits, i.e., a flash induces two charge separations [see, e.g., Dau et al. (2012) for a more mechanistic model for the period-4 oscillations]. The only major change is that a population of about 25% RCs, which Kok et al. ascribed to PSII RCs in the *S*0 state in darkness, is now ascribed to electron donation by reduced TyrD to the Mn cluster between flashes (Velthuys and Visser 1975; Vermaas et al. 1984; Shinkarev 2005). Donation of an electron by TyrD to the Mn cluster is a slow reaction and can, in continuous light, not compete with the light-induced turnover of the OEC. In dark-adapted samples all RCs can be considered to be in the *S*1 state. Period-4 oscillations can be used to analyze the *S* state distribution of a sample (cf. Ioannidis et al. 2000). This analysis can even be extended to super-reduced states such as the *S*-1 and *S*-2 states (Schansker et al. 2002). Period-4 oscillation in the *F*_*O*_ level can be used to follow the decay kinetics of the *S* states under natural conditions, but also in response to external electron donors like hydroxylamine or NO (see Ioannidis et al. 2000). The time resolution of such measurements depends on the flash frequency, which is, in practice, around 10 Hz (one flash every 100 ms). This type of measurement can also be carried out with flash illumination of a sample on a bare oxygen electrode (e.g., Messinger and Renger 1990, 1993). The disadvantage of a bare oxygen electrode is that a rapid injection and mixing of reactants is not possible (Messinger and Renger 1990). Period-4 oscillations are usually measured on PSII enriched membranes, thylakoid membranes or algal cells. David Kramer and coworkers introduced, in 1990, an instrument to measure period-4 oscillations on leaves in the field (Kramer et al. 1990), although few studies using this instrument have been published.

**Fig. 4:**
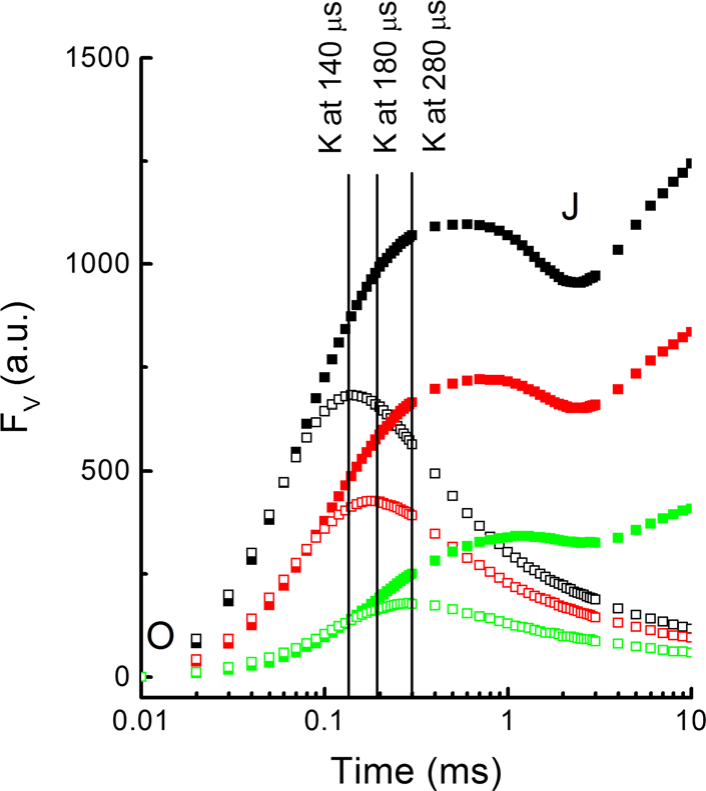
Chl *a* fluorescence transients of untreated (*closed symbols*) and severely high-temperature-stressed (*open symbols*) barley leaves illuminated with 5000 (*green symbols*), 10,000 (*red symbols*) and 15,000 (*black symbols*) µmol photons m^−2^ s^−1^. To allow a comparison of the kinetics the transients were shifted to 0 to have for all transients the same *O* value. The time needed to reach the K peak at the different light intensities is indicated. The K peak equates approximately 1 charge separation in all RCs after which a shortage of electrons that can be donated by the donor side occurs, and the fluorescence intensity decreases due to re-oxidation of *Q*_*A*_^−^ reduced by the first charge separation (modified from Tóth et al. 2007a)

In response to heat stress, the Mn cluster can be destroyed. Heat stress can cause a dissociation of extrinsic proteins, followed by a super-reduction of the Mn cluster, which destabilizes it, resulting in a disintegration of the Mn cluster and a release of the Mn ions into the lumen (Yamane et al. 1998; Pospíčil et al. 2003; Barra et al. 2006). In the case of severe heat stress, the OJ rise turns into a K peak (e.g., Srivastava et al. 1997; Fig. 4). It has been proposed that the K peak, that can be made visible by subtraction of the double-normalized OJ rise, can be used as a probe of PSII RCs with an inactive donor side (e.g., Smit et al. 2009; Yusuf et al. 2010). However, as discussed in Kalaji et al. (2014a), this is tricky, because the same phenomenon is also induced by differences in the PSII antenna size between samples and a difference in the redox state of the PQ pool (Strasser et al. 2001). There are, however, two alternative approaches available to researchers. At approximately 3000 µmol photons m^−2^ s^−1^ one charge separation in all reaction centers takes about 200 µs (K step). PSII RCs with an inactive donor side are capable of a single charge separation and during this time interval the fluorescence rise is the same in active and inactive RCs (Tóth et al. 2007a; Fig. 4). Subsequently, TyrZ is re-reduced with a half-time of about 30 ms (Tóth et al. 2007a, 2009) and during this time no second stable charge separation can occur. This means that the *K* to *J* rise is slowed down as a function the fraction of PSII RCs with an inactive donor side. Making use of this phenomenon, the parameter *F*_*K*_/*F*_*J*_ was introduced to probe the extent of inactivation of the PSII donor side (Srivastava et al. 1995; Lu and Zhang 1999). Making use of the difference in the regeneration time of the donor side, it is also possible to give two strong 5-ms pulses spaced 2.3 ms apart (the dark interval when the pulse interval of the HandyPEA is set to 0). The ratio of the fluorescence intensity at 300 µs of the second and the first pulse can then be used as a measure of the fraction PSII RCs with an inactivated donor side (Oukarroum et al. 2009; see this paper also for a discussion of the different approaches).

In summary, to probe the fraction of PSII RCs with an inactive donor side, it is possible to make use of the difference in the regeneration time of the donor side of PSII (the re-reduction of P680^+^ and TyrZ^+^).

## Question 15: What is the character of the J step?

The J step (*F*_2ms_ or *F*_3ms_) forms the central point in the JIP test analysis. Giving the J step such a central role was one of the major innovations introduced by Strasser and Strasser (1995) together with the consistent use of a logarithmic time base to make the different steps of the OJIP rise visible. The J step of Strasser and coworkers equates the I1 step of Schreiber (Schreiber 1986; Neubauer and Schreiber 1987; Schreiber and Neubauer 1987). Compare for this equivalence panels A and B of Fig. 1 in Lazár (2006). In publications about the analysis of OJIP transients the time point taken for the J step is either at 2 or at 3 ms (e.g., Strasser and Strasser 1995 vs. Tóth et al. 2007b). Stirbet and Govindjee (2012) argued that it had to be 2 ms because this was the time point chosen by Strasser and Strasser (1995) for the JIP test. Kinetically, the choice is important. At low light intensities or for PSII reaction centers with a smaller antenna size the time needed to reach the J step is more than 2 ms, and this provides additional variability, which may be interesting for a stress test. For people interested in the kinetics of the photosynthetic electron transport chain, the 3-ms point is the more logical choice. It is at the end of the step, where the traffic jam of electrons induced by the rate-limiting step presented by the exchange of reduced PQH_2_ for PQ is maximal. As shown in the literature, this time point remains the end of the J step independent of the light intensity; lowering the light intensity gradually makes *F*_3ms_ disappear (Schansker et al. 2005, 2011).

Another phenomenon observed, when using very high light intensities, is a dip occurring around 2–3 ms (Neubauer and Schreiber 1987; Schansker et al. 2005, 2011). A possible explanation for this dip is the presence of P680^+^, which acts as an even stronger fluorescence quencher than *Q*_*A*_ (Steffen et al. 2005; see Question 22). The lifetime of P680^+^ is short for all *S* states, with the exception of the transition from the *S*3 to *S*4 state. As the light intensity is increased, the turnover of PSII will remain more synchronized during the first turnovers and the peak concentration accompanying the *S*3 to *S*4 state transition will not only occur at shorter times, but will also reach a higher peak value causing a stronger and more localized quenching effect.

It has been assumed that the J step is due to the rate limitation caused by the exchange of *Q*_*B*_H_2_ for PQ (cf. Petrouleas and Crofts 2005; Schansker et al. 2005; Tóth et al. 2007b). The role of P680^+^ quenching discussed above suggests that the transitory accumulation of P680^+^, in going from the *S*3 to the *S*4 state (after 3 charge separations), is the physical reason for the existence of the J step. For dark-adapted leaves and continuous light both processes occur synchronously. Using a preflash protocol, a desynchronization between PSII donor and acceptor sides can be induced. This happens following two preflashes. This creates the *S*3ZP680*Q*_*A*_ *Q*_*B*_^−^ state, where the *S*3 state is quite stable during a, e.g., 100-ms flash interval and *Q*_*B*_H_2_ is exchanged within 2 ms for PQ. If 100 ms after the two preflashes the OJIP transient is measured, the induction kinetics of the fluorescence rise are quite drastically changed (Schreiber and Neubauer 1987; Strasser and Strasser 1998).

## Question 16: What is the information that can be derived from the Area parameter?

Joliot and Joliot (1964) published evidence that the relationship between variable fluorescence and *Q*_*A*_ redox state in the presence of an inhibitor (e.g., DCMU) that prevents the re-oxidation of *Q*_*A*_^−^ is nonlinear. The authors proposed that this nonlinearity was due to the exchange of excitation energy between different PSII antennae. As a consequence the relationship became sigmoidal. Two years later, Malkin (1966) and Murata et al. (1966a) came up with an alternative. They proposed that the complementary area between *F*_*M*_ and the fluorescence transient measured in the presence of an inhibitor of re-oxidation of *Q*_*A*_^−^ is linearly related to the *Q*_*A*_^−^ concentration. This was confirmed experimentally by Bennoun and Li (1973). Although this idea can still be applied, several complications have been identified.

First, the complementary area of fluorescence induction curves measured in the presence of DCMU was shown to consist of several rise components. Doschek and Kok (1972) concluded that the complementary area represented a two-electron process. Melis and Homann (1975, 1976) interpreted this phenomenon to represent PSII heterogeneity: PSII alpha and beta centers differing in antenna size. Subsequently, two more phases were identified, designated gamma and delta (e.g., Sinclair and Spence 1990).

Then it was discovered that the area was very sensitive to the place where the *F*_*M*_ was set (Bell and Hipkins 1985). Particularly, in cases where part of the PSII RCs remained uninhibited, it took a long time before *F*_*M*_ was reached. As a consequence, the area grew strongly. In the pre-LED period, when shutters with opening times of 0.8–2 ms were used, measurements in the presence of inhibitors like DCMU had to be made at very low light intensities to record as much as possible of the fluorescence transient. With modern shutterless LED-based systems, such measurements at light intensities of 3000 µmol photons m^−2^ s^−1^ or more can be made without any problem. Such measurements have much better defined *F*_*M*_ levels, which strongly reduces the problem with inhibited PSII RCs.

Using high light intensities, area ratios of alpha/beta/gamma = 0.58:0.33:0.06 were obtained for pea leaves (Tóth and Strasser 2005). With a simulation approach, Lazár et al. (2001) obtained similar values for wheat leaves: 0.64:032:0.04. The percentage of gamma centers agrees well with the percentage of *Q*_*B*_ non-reducing centers determined using another method (Schansker and Strasser 2005). Based on present knowledge, the area growth of alpha centers should parallel the reduction of *Q*_*A*_.

It should be noted that Trissl and Lavergne (1995) argued that changing rate constants during induction would preclude the use of the area as a measure for *Q*_*A*_^−^. However, if we assume that almost each charge separation reduces *Q*_*A*_ (quantum yield of approximately 0.88, see Question 6), then the area growth simply follows the gradual reduction of *Q*_*A*_ in all PSII RCs and does not depend on different reactions and changing rate constants in individual RCs.

The area approach has also been applied to OJIP transients, for example, in the JIP test (Strasser and Strasser 1995; Strasser et al. 2004), but also by Joliot and Joliot (2002) and Tóth et al. (2007b). Joliot and Joliot (2002) showed that the regeneration of the area above the OJIP transient following a saturating pulse of light is defined by two exponential phases that they ascribed to the reoxidation of the acceptor side of PSI (fast phase) and the reoxidation of the PQ pool (slow phase). Tóth et al. (2007b) manipulated the redox state of the electron transport chain using anaerobiosis and showed that both the area between *F*_*M*_ and the OJ rise (0–3 ms) and the area between *F*_*M*_ and the JI rise (3–30 ms) linearly correlate with the *F*_*J*_ intensity.

The area above the OJIP transient is dominated by the area above the JIP rise. Schansker et al. (2011) have provided evidence that the character of the JIP rise differs from that of the OJ rise. During the JIP rise the fluorescence yield per reduced molecule of *Q*_*A*_ reduced increases. In this respect, the calibration of the area above the OJIP transient by the area between the J step and the OJ rise, as done in the JIP test, may not be the best approach. Instead, it is better to use the area between the OJ rise and *F*_*M*_, taking *J* at 3 ms. In this way, the fluorescence yield increase is taken care of. This area would equate approximately 3 electrons, i.e., the reduction of the acceptor side of PSII. The JIP test approach yields for dark-adapted, non-stressed leaves a value *N* of about 30 electrons (=total Area divided by the area between the OJ rise and the J step). Joliot and Joliot (2002) observed that PQ pool and PSI acceptor-side pool size were similar. For the JIP test approach this means 7–8 PQ molecules per PSII, which agrees quite well with a calculation of Lavergne et al. (1992). Kirchhoff et al. (2000), on the other hand, concluded that in stacked thylakoid membranes there are about 3–4 PQ molecules per PSII, which is a considerably lower value. The approach of Tóth et al. (2007b), using the area between OJ rise and *F*_*M*_, yields for *Phaseolus vulgaris* leaves a value *N* of approximately 31 and about 7 PQ molecules. Although the calculation in both cases is quite different, the outcome is quite similar.

The conformational change concept gives a rationalization for the relationship between area and electron transport chain redox state (Schansker et al. 2011, 2014). The induction of the conformational changes depends on the time interval *Q*_*A*_^−^ is reduced before being reoxidized, and this in turn depends on the redox state of the electron transport chain.

The use of the Area—if normalized—is an effective way to detect changes in the redox state of the electron transport chain or changes in the stoichiometry PSII/PQ pool/PSI acceptor side of unstressed leaves. For stressed leaves, the Area can only be used if it has been ascertained that several criteria are met: The *F*_*M*_ should be reached and the rate constants of different electron transport reactions should not be affected (too much). In the case of high-temperature stress, individual PSII RCs are knocked out. The remaining PSII reaction centers have to reduce more electron acceptors before *F*_*M*_ is reached. In this case, the increase in the Area should be a function of the extent of inhibition of the PSII RCs as long as *F*_*M*_ is still reached, which in severe cases is no longer true (Tóth et al. 2005a; see transients in Srivastava et al. 1997).

In summary, the Area is a useful tool to probe electron transport chain capacity.

A related parameter is $t_{{F_{m} }}$, the time needed to reach *F*_*M*_. A physiological characterization of this parameter is still missing. It seems likely that this parameter has a strong sensitivity to the PSII/PSI ratio and the size of the PSI acceptor-side pool.

## Question 17: Can the 77 K fluorescence emission bands be assigned to specific photosynthetic complexes or processes?

**Fig. 5:**
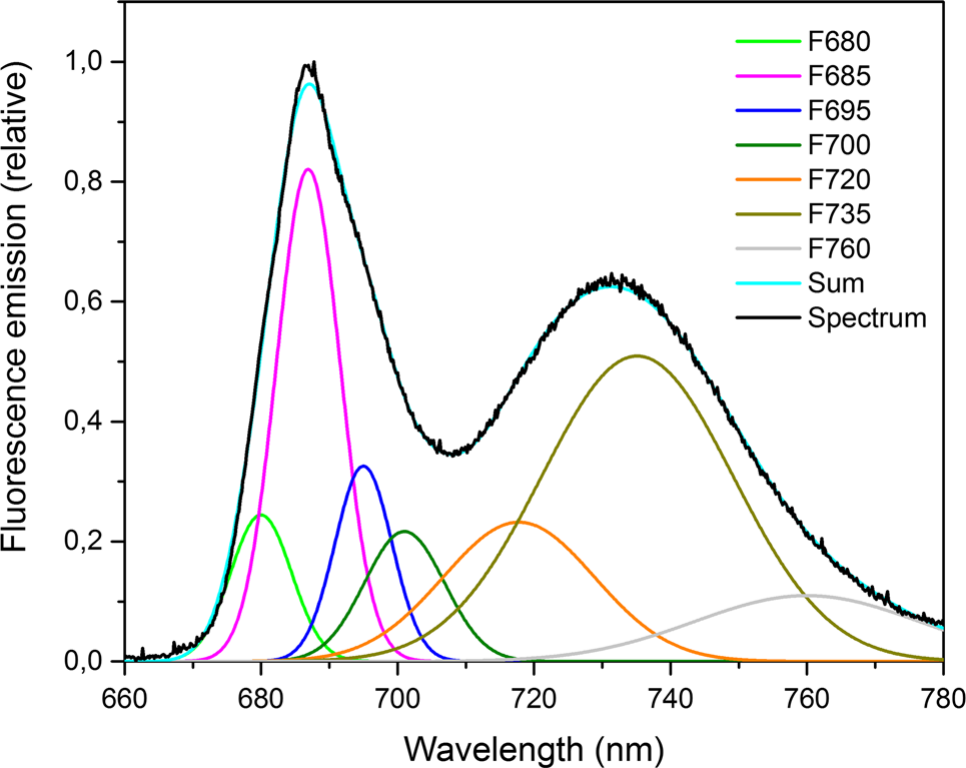
77 K spectrum measured on *Arum italicum* thylakoid membranes with its deconvoluted bands indicated. For the assignment of the bands see the text of Question 17 (Pancaldi and Ferroni, unpublished data)

The interpretation of 77 K fluorescence emission spectra of cyanobacteria, algal cells and higher plants discussed in the previous paper (Kalaji et al. 2014a) was based on the analysis of fluorescence emission spectra measured on isolated complexes of, e.g., CP47 and CP43. The assignment of especially the bands associated with PSII has changed considerably in the last 10 years. In Fig. 5 an example of a 77 K spectrum (of *Arum italicum* thylakoid membranes) with the deconvoluted bands indicated is given.

The commonly accepted assignment of F695 to CP47 and F685 to CP43, as mentioned above, is based on the analysis of the spectral properties of isolated complexes (Nakatani et al. 1984; van Dorssen et al. 1987; Alfonso et al. 1994; Groot et al. 1999).

Andrizhiyevskaya et al. (2005), studying the temperature dependence (4–293 K) of the fluorescence emission spectra, confirmed that F695 is emitted by CP47. The complex contains a Chl that absorbs light at 690 nm and excitation energy trapped by this Chl can only be transferred to the RC by uphill energy transfer. At low temperatures (77 K or lower) this extra energy cannot be drawn from the environment and the excitation energy is irreversibly trapped giving rise to F695. Chls absorbing at 683 nm are found not only in CP43 but also in CP47. Also for excitation energy trapped on these Chls an uphill transfer to the RC is needed, but the energy difference is smaller. Lowering the temperature only leads to a slowdown of energy transfer. Andrizhiyevskaya et al. (2005) further observed the F685 band in CP47-RC complexes, but its intensity was smaller than in core complexes that also contain CP43. The authors concluded that F685 should be assigned to specific Chls in both CP47 and CP43.

The subunit approach came under criticism by a study of Krausz et al. (2005) who compared the emission of PSII holocomplexes with those of isolated LHCII, CP43, CP47 and a minimal PSII core unit formed by D1/D2/cyt*b*_559_ and concluded that a combination of subunit spectra does not add up to the emission spectrum of intact PSII. Like Andrizhiyevskaya et al. (2005), the authors suggested that the PSII fluorescence emission spectrum depends on energy transfer bottlenecks due to a few Chl molecules in CP43 (ca. 2.5 Chls) and CP47 (ca. 1 Chl).

In the majority of the earlier studies a significant emission contribution from PSII RCs was excluded. Chen et al. (2015) made an extensive comparison between published results and new fluorescence emission spectra recorded on isolated PSII cores of spinach and from re-dissolved PSII core crystals of the cyanobacterium *Thermosynechococcus elongatus*. The authors showed that isolation procedures that preserve the intactness of the PSII core result in nearly overlapping 77 K emission spectra of cyanobacteria, the green alga *Chlamydomonas reinhardtii* and spinach. Four main emission sources governing the spectral outline in the PSII region were identified: (1) F695, originating from intact CP47; (2) two emission bands from destabilized CP47, peaking at 691 nm (FT1, matching that of isolated CP47 found in previous studies by van Dorssen et al. 1987) and at 685 nm (FT2); (3) emission near 686–687 nm, originating from pigments of the PSII reaction center. It was also concluded that CP43, whose emission peak is actually at 683.3 nm and not at 685–686 nm (Dang et al. 2008; Reppert et al. 2010), is not an important emission source of F685 in intact PSII. The authors also refer to the observation of Sun et al. (2015) that interaction of LHCII with purified PSII core increases F685 considerably, which would further support their assignment of F685 to PSII RC pigments. In other words, in intact PSII core, the main source for F685 is proposed to be the PSII reaction center.

D’Haene et al. 2015 came with an alternative interpretation of F695. In PSII mutants of the cyanobacterium *Synechocystis* lacking PsbH, a small PSII subunit, F695 is not detected. The authors suggest that the interaction of PsbH with a single monomeric Chl ligated to His114 of CP47 is responsible for F695.

The story of the PSII 77 K emission band assignment is a good illustration of the potential problems of using isolated subunits to study processes affecting the whole complex. In this case, the whole is more than the sum of the parts.

The assignment of the fluorescence emission associated with PSI at around 720 nm is more straightforward. The major fluorescence emission band of PSI is remarkably variable among different species (Murata et al. 1966b) with peak emission occurring between 710 and 730 nm. Isolation of subunits from PSI particles allowed a more detailed characterization of PSI emission (Mullet et al. 1980; Argyroudi-Akoyunoglou 1984; Kuang et al.^1984^). In higher plants two main emission sources were identified at 77 K: F720 and F735 (Mukerji and Sauer 1990; Pålsson et al. 1995). In the green alga *Chlamydomonas reinhardtii*, LHCI-PSI emission is quite blue-shifted compared to higher plants, i.e., a peak at ca. 715 nm instead of 735 nm (Garnier et al. 1986; Ferroni et al. 2011). Croce et al. (1997) showed that actually three Chl spectral forms exist in native PSI from maize thylakoids, having emission maxima at 720, 730 and 742 nm. Analyzing barley mutants lacking individual LHCI subunits, Knoetzel et al. (1998) linked the Lhca1 and Lhca4 subunits of LHCI to emissions at 732 and 742 nm, respectively, but only when such LHCI antennae were associated with PSI. A simplified band attribution can, therefore, be proposed: PSI core emits F720, but is also characterized by a short wavelength emission at 690 nm (Croce et al. 1997). Assembly of LHCI-PSI complexes gives rise to longer wavelength emissions with increased intensity, collectively giving rise to F735. In cyanobacteria, where PSI typically is organized in trimers instead of monomers, a further redshift in emission gives rise to F760 (Shubin et al. 1992; Karapetyan et al. 2014). Origin of PSI fluorescence bands is attributed to the “red chlorophylls” bound to PSI core, Lhca1/4 and Lhca2/3; the emission sources are probably dimers, trimers or aggregates of Chls which absorb light at wavelengths longer than 700 nm, necessitating uphill energy transfer at 77 K as described above for PSII (reviewed by van Grondelle and Gobets 2004).

An important contribution to the 77 K spectra comes from LHCII. The presence of LHCII, formed by trimers of Lhcb1-3 subunits in higher plants, gives the photosystems a high degree of flexibility, which is still the subject of intense study. The following interactions have been observed so far: (1) LHCII association with PSII core dimers to form LHCII-PSII supercomplexes (Boekema et al. 1999); (2) LHCII association with PSI in the so-called LHCII-PSI state transition complex to balance excitation between PSI and PSII (Pesaresi et al. 2009; Wientjes et al. 2013b); (3) aggregation of LHCII trimers related to the transition from the harvesting to the quenched state (*q*_E_) (Johnson et al. 2011); (4) monomerization of LHCII trimers upon long-term acclimation to high light to reduce energy transfer to photosystems (Bielczynski et al. 2016); (5) LHCII trimers surrounding PSI-PSII megacomplexes (Yokono et al. 2015) and/or forming a bridge between PSI and PSII in such megacomplexes (Grieco et al. 2015; Suorsa et al. 2015) mediating photoprotective energy spillover from PSII to PSI (Yokono et al. 2015; Ferroni et al. 2016). Because LHCII can serve PSII and/or PSI, the relative size of F685 and F720 is dependent on the pre-illumination conditions (see also Question 8).

The F680 emission of LHCII, as demonstrated in isolated LHCII monomers and trimers (Kirchhoff et al. 2003; Caffarri et al. 2004; Karapetyan 2004), is not usually found as a distinct peak in 77 K spectra of thylakoids, reflecting the very efficient excitation energy transfer from LHCII to PSII in LHCII-PSII supercomplexes. Association of LHCII with PSII core increased the intensity of F695 (greening pea plants, Srivastava et al. 1999) and/or F685 (in vitro incorporation of LHCII in PSII cores, Sun et al. 2015), testifying to an increased efficiency of exciton transfer to the reaction centers. Conversely, release of LHCII trimers enhances F680. There are several lines of evidence linking F680 to free LHCII, among which LHCII release from grana cores induced by thylakoid unstacking (van der Weij-de Wit et al. 2007), LHCII release from grana margins induced by digitonin treatment of isolated thylakoids (Grieco et al. 2015), and also steady-state conditions in which LHCII trimers are overly abundant as compared to photosystem cores (Pantaleoni et al. 2009; Ferroni et al. 2013). Native LHCII trimers have a strong tendency to aggregate in vitro, leading to the appearance of a new long-wavelength band, F700, also accompanied by a quenching of fluorescence, which could support a role of LHCII aggregates in excess energy dissipation (Mullet et al. 1980; Ruban and Horton 1992; Karapetyan 2004; Schaller et al. 2011). F700 has been recorded also in vivo in intact leaves (Šiffel and Braunová 1999; Oh et al. 2003). The LHCII aggregates that originated in leaves upon a CO_2_ deficit yielded a F700 band with high fluorescence yield (Šiffel and Braunová 1999). It is likely that, in vivo, different populations of LHCII aggregates can be formed, either engaged in thermal dissipation or not.

In summary, much progress has been made with respect to the assignment of fluorescence emission bands to particular subunits at 77 K. For the applied user, this assignment is important for the interpretation of changes in the emission spectra, especially in the case of PSII mutants or environmental stress effects. The molecular mechanisms behind the emission bands represent key information for the simulation of these bands (e.g., Reppert et al. 2010; Renger and Schlodder 2011).

## Question 18: What is the relationship between delayed and prompt fluorescence?

**Fig. 6:**
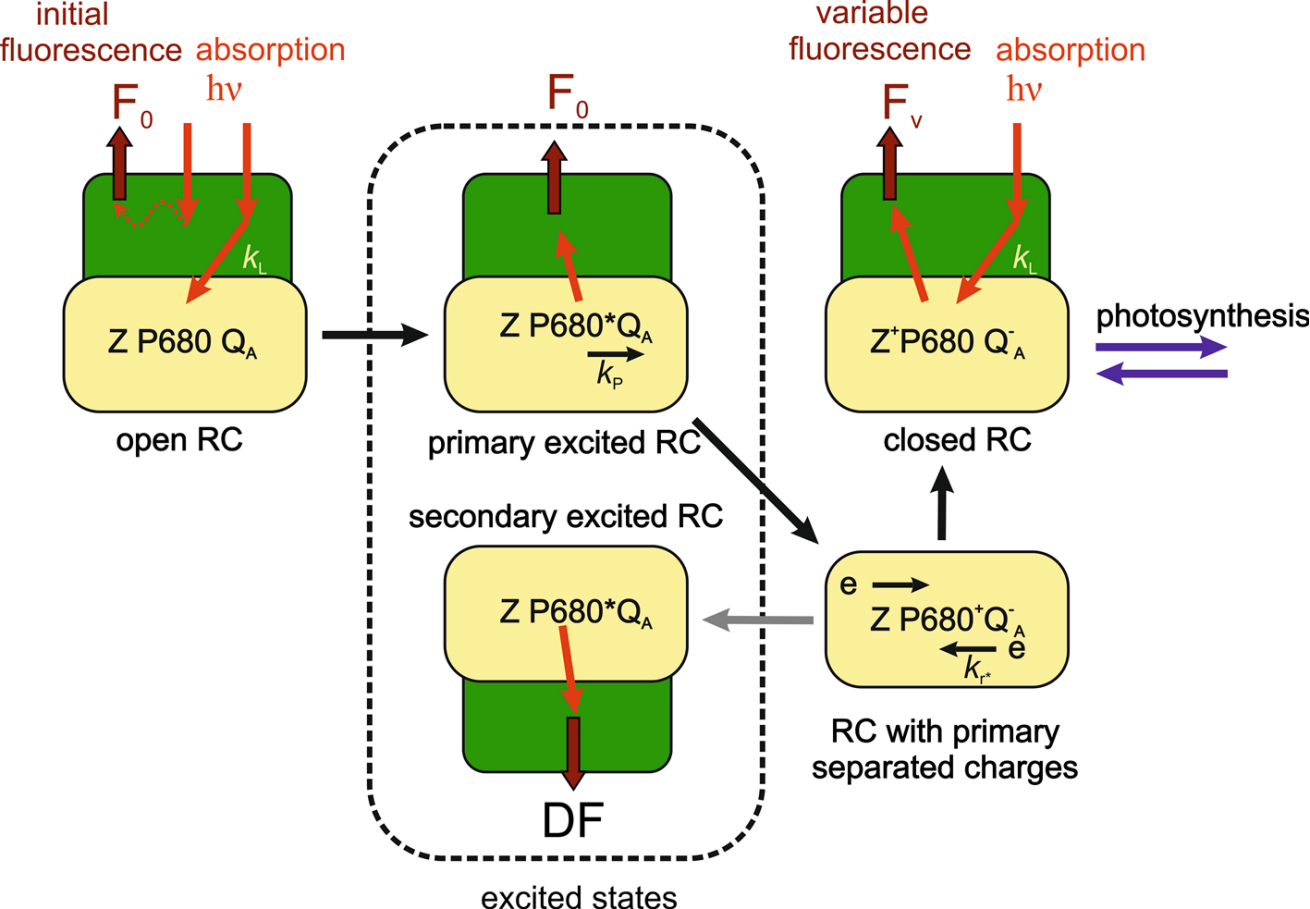
Illustration of the difference between *F*_*O*_ and DF. Following absorption of a photon the excitation energy may be lost in the antenna and re-emitted as fluorescence (*F*_*O*_). The excitation energy may induce a charge separation, which may be followed by electron transfer to *Q*_*A*_ or a recombination reaction. In the latter case the energy may again be lost as fluorescence (*F*_*O*_). Emission occurs at subnanosecond times following excitation. Fluorescence emission induced by recombination between *Q*_*A*_^−^ and the PSII donor side leads to a delay in the emission time with the fluorescence emission occurring ~40 µs or longer following excitation and is called delayed fluorescence (DF) (Goltsev, unpublished data)

Prompt fluorescence (PF) is due to the emission of light following absorption of a photon caused by the return of an excited Chl molecule to the ground state, which takes only 1.5–1.8 ns (Brody and Rabinowitch 1957; Barber et al. 1989; Krause and Weis 1991). Strehler and Arnold (1951) discovered that there is a second type of fluorescence emission that can be detected for quite long times after switching off the light. This type of fluorescence is related to recombination reactions, which, depending on the recombining charge pair, occur with a lifetime of ~40 µs for P680^+^/*Q*_*A*_^−^ (Govindjee and Jursinic 1979) or tens of seconds in the case of the recombination between *S*2 or *S*3 and *Q*_*B*_^−^ (e.g., Rutherford et al. 1984). Half-times for the recombination between *Q*_*A*_^−^ and P680^+^ determined for samples with a destroyed donor side (Tris-washed) give a half-time of about 120–130 µs (Conjeaud and Mathis 1980; de Wijn and van Gorkom 2002). This type of fluorescence emission is called delayed fluorescence (DF) (e.g., Wraight and Crofts 1971; Goltsev et al. 2009) or delayed light emission (DLE) (e.g., Arnold and Thompson 1956; Srivastava et al. 1999) and is much weaker (about 100-fold) than PF (Jursinic and Govindjee 1982; Arnold 1991). In summary, the lifetime of different DF components is determined by the lifetime of the corresponding PSII state (charge pair). The emission spectrum of prompt and delayed fluorescence is identical (Strehler and Arnold 1951; Arnold and Thompson 1956; Sonneveld et al. 1980; Grabolle and Dau 2005). As described by the reversible radical pair model of Schatz et al. (1988) recombination can lead to the formation of “secondary excited” Chl*. This excitation energy can be transferred back to the antenna. There it is emitted by the same mechanism as PF (e.g., Grabolle and Dau 2005). Like PF, DF is predominantly a PSII phenomenon and it always involves a recombination between a negative charge on the acceptor side of PSII and a positive charge on the donor side of PSII. Figure 6 shows a schematic representation of the conditions under which PF and DF emission occurs. Details on the characteristics of DF can be found in a number of reviews (Lavorel 1975; Amesz and van Gorkom 1978; Malkin 1979; Lavorel et al. 1982; Jursinic 1986; Veselovskii and Veselova 1990; Gaevsky and Morgun 1993; Radenovic et al. 1994; Tyystjärvi and Vass 2004; Goltsev et al. 2009; Kalaji et al. 2012a). The older reviews predate the introduction of the reversible radical pair mechanism and contain extensive discussions on the mechanism of DF. These articles are good sources for experimental DF phenomena though.

## Question 19: What is the relationship between delayed fluorescence (DF) and thermoluminescence (TL)?

As noted above, each charge pair has its own particular recombination time. Based on their specific lifetimes, the different charge pairs can be identified and their population/amplitude and recombination rate characterized (reviewed by, e.g., Vass and Govindjee 1996; Vass 2003). Thermoluminescence (TL), light emission as a result of temperature-dependent recombination of charge pairs, is a special application of DF, although it has to be noted that not all DF phenomena have a TL counterpart (Vass and Govindjee 1996). In addition, there are high-temperature TL bands that are due to oxidative chemiluminescence (50–70 °C) (Hideg and Vass 1993; Skotnica et al. 1999) and lipid peroxidation (130–140 °C) (Ducruet and Vavilin 1999). As the temperature is lowered, charge recombinations become gradually inhibited. Below approximately −40 °C/−45 °C no charge recombinations occur anymore and induced charge pairs can be stabilized (Brudvig et al. 1983). For TL, there are different illumination strategies. It is possible to give one or more single turnover flashes at, for example, −20 °C, at which temperature it is still possible to induce stable charge separations in practically all reaction centers, followed by a rapid lowering of the temperature to stabilize the induced state. Alternatively, it is possible to illuminate a sample for a much longer time in the range −60 to −80 °C to induce a single charge separation in all reaction centers (Brudvig et al. 1983). This does not induce Chl or Car radicals as suggested by Stirbet and Govindjee (2012); to induce these radicals the temperature has to be lowered further (e.g., Faller et al. 2001). Inversely, upon warming the sample, the different charge pairs can gradually recombine; each charge pair with its own typical peak temperature (Vass 2003). Longer recombination times go together with higher TL peak temperatures. The *Q* band, the charge recombination between *Q*_*A*_^−^ and the *S*2 or *S*3 state of the OEC, peaks around 0 °C, whereas the B band, the charge recombination between *Q*_*B*_^−^ and the *S*2 or *S*3 state of the OEC, peaks at considerably higher temperatures. TL measurements become distorted during the transition from the frozen to the thawed state (Vass et al. 1981). To minimize the effects of this transition, different solutions have been found, for example a stronger heating around 0 °C (Ducruet and Miranda 1992) or the presence of 30 or 60% glycerol (Demeter et al. 1985). See Ducruet and Vass (2009) for a practical guide to the application of TL.

TL measurements are destructive. A sample does not survive the freezing/heating cycle that is applied during a measurement. DF measurements, on the other hand, are non-destructive. Even so, TL measurements have been more popular than DF measurements due to the absence of commercially available instruments for the measurement of DF. Such an instrument is now available on the market (M-PEA, Hansatech Instruments Ltd, UK; see Question 21). It may also be mentioned that the analysis of TL measurements is better developed (DeVault et al. 1983; Vass 2003) than the analysis of DF transients. TL measurements have been used extensively for the characterization of PSII mutants (e.g., mutants that affect charge recombination properties) (e.g., Etienne et al. 1990; Cser and Vass 2007) and treatments that change the state of PSII (e.g., Cl^−^ and Ca^2+^ depletion from the donor side of PSII or the effects of the presence of herbicides bound to the *Q*_*B*_ site of PSII) (e.g., Krieger et al. 1993; Demeter et al. 1993; Bock et al. 2001). It is also a convenient technique for the study of environmental stresses that affect PSII (e.g., Mohanty et al. 1989; Hideg et al. 1993; Tóth et al. 2005a).

## Question 20: Does a recombination reaction always lead to DF emission?

PF intensity is linearly related to the actinic light intensity (see, e.g., Schansker et al. 2006). In the case of DF only absorbed light that induces a stable charge separation can lead to DF and especially at high light intensities, this is only a small fraction of the absorbed light. In addition, DF emission is much more spread out in time because the DF-inducing charge recombinations can be due to different charge pairs, with different recombination times. A third factor that reduces the DF yield is the existence of several recombination pathways of which only one leads to DF (see, e.g., Krieger-Liszkay and Rutherford 1998 and Cser and Vass 2007 for a discussion of this point). Cser and Vass (2007) give an overview of the different pathways: (1) a direct recombination between *Q*_*A*_^−^ and P680^+^ via tunneling, which is non-radiative; (2) recombination via the triplet state of P680^+^ Pheo^–^, which is also non-radiative, but there is an increased probability of singlet oxygen formation (see Question 13); (3) recombination via the singlet state of P680^+^ Pheo^−^, which decays via light emission. Pathways 2 and 3 are called indirect charge recombination pathways. Krieger-Liszkay and Rutherford (1998) showed that DCMU bound to the *Q*_*B*_ site increases the midpoint potential of *Q*_*A*_, stabilizes the charge separation (shift of TL bands to higher temperatures) and increases the probability that recombination occurs via tunneling (pathway 1). Bromoxynil, on the other hand, decreases the midpoint potential of *Q*_*A*_, destabilizes the charge separation (shift TL bands to lower temperature) and increases the probability that recombination occurs via pathways 2 and 3). Cser and Vass (2007) confirmed this interpretation using mutants in which amino acids near Pheo and P680 were modified using site-directed mutagenesis. The authors showed that the free energy difference between P680 in the excited state and P680^+^Pheo^–^ is an important determinant of TL emission intensity.

## Question 21: How can PF and DF be simultaneously measured?

PSII reaction centers and antennae emit about 100 times more PF than DF. In addition, the emission spectra of both types of fluorescence emission are identical (Strehler and Arnold 1951; Arnold and Thompson 1956; Sonneveld et al. 1980; Grabolle and Dau 2005) (see Question 18). As a consequence, both cannot be detected at the same time and thus a separation strategy is needed. Two approaches for the simultaneous monitoring of PF and DF signals have been developed: (1) quasi-continuous illumination of dark-adapted samples and (2) illumination of a dark-adapted sample by single pulse (usually by a laser) [see, e.g., Steffen et al. (2001) for such a experimental setup and Belyaeva et al. (2015) for a discussion of laser-flash-induced fluorescence data], or by continuous illumination. The results of these two methods differ.

The first approach has been used for the evaluation of the induction kinetics of the two fluorescence signals over short time periods (seconds) (Wraight and Crofts 1971; Zaharieva and Goltsev 2003; Strasser et al. 2010), whereas the second approach has been used for both the analysis of induction kinetics (e.g., Schansker et al. 2011) and the analysis of PF and DF relaxation kinetics within wider time intervals (minutes to hours) (Katsumata et al. 2008, Berden-Zrimec et al. 2011).

The first approach has been successfully implemented using the Multifunction Plant Efficiency Analyzer (MPEA, Hansatech Instruments Ltd, UK). The sample is illuminated by a series of red light pulses of variable duration (produced by light-emitting diodes), followed by short dark periods (duration: 1/3 of the light pulses). This type of illumination is often called quasi-continuous. Prompt fluorescence is measured during the illumination periods, and the decaying DF signal is recorded during the dark intervals. The maximum rate of digitization of both signals in the M-PEA is 1 point per 10 μs. It should be noted that the dark intervals reduce the effective light intensity by one-third. Thus, 5000 µmol photons m^−2^ s^−1^ light pulses yield an effective light intensity of 3334 µmol photons m^−2^ s^−1^. In addition, during the dark intervals the dark reactions continue (electron flow), whereas the light reactions (charge separations) stop, changing the relationship between dark and light reactions.

**Fig. 7:**
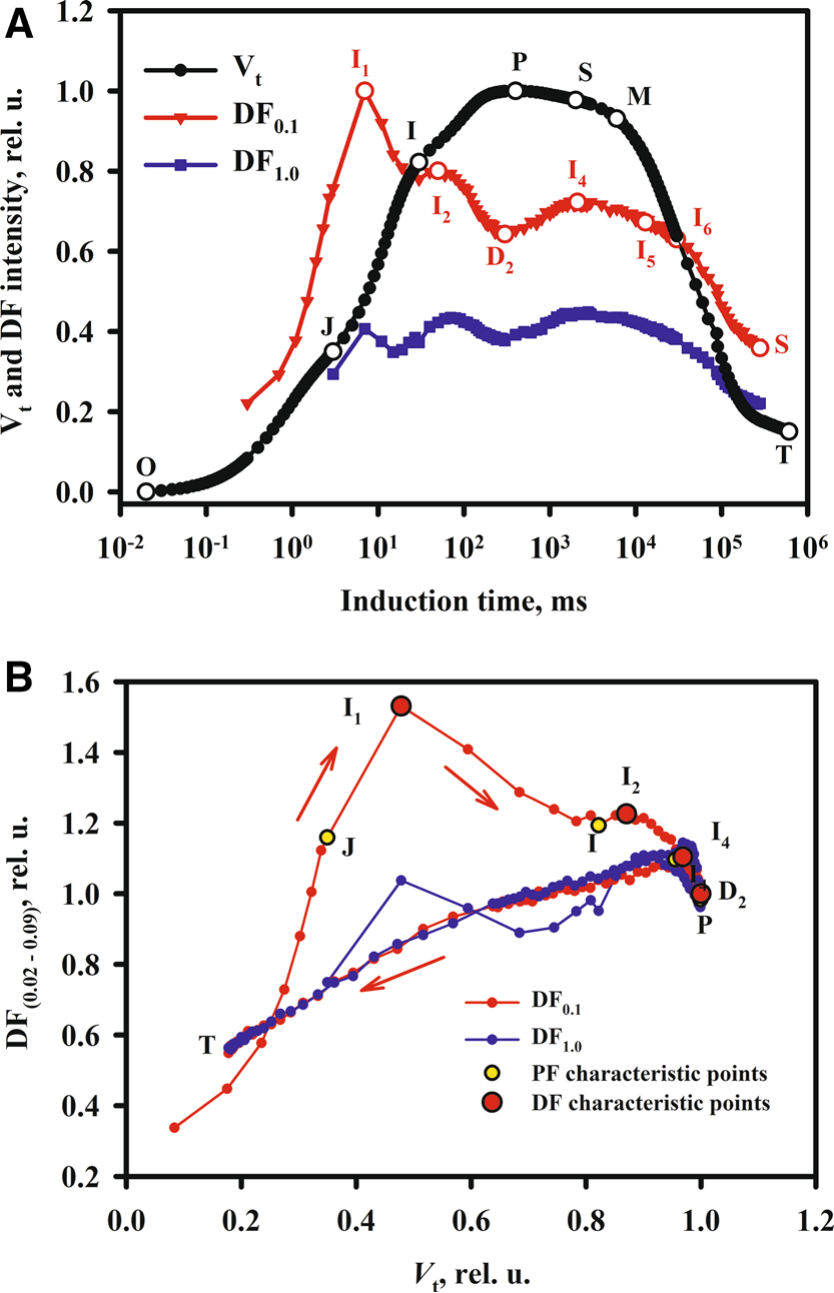
Kinetics of prompt fluorescence (PF) induced by a pulse of 5000 µmol photons m^−2^ s^−1^, interspaced by short intervals of darkness to measure the delayed fluorescence (DF) kinetics. **a** PF intensity, DF intensity following 0.1 ms of darkness and the DF intensity following 1 ms of darkness as a function the time of illumination; **b** DF intensity following 0.1 and 1 ms of darkness as a function of the PF intensity (Goltsev, unpublished data)

Using quasi-continuous illumination, DF induction transients complementing the PF induction transients can be constructed. To do this, the DF intensities measured during a particular dark interval for all dark periods are selected and averaged, and then used as single points for the DF induction curve. By selecting different decay intervals, induction curves can be constructed that show DF kinetic components with different lifetimes (Kalaji et al. 2012a; Fig. 7a).

Using the second approach, Chl *a* fluorescence decay is recorded after a short (ns) laser pulse or after continuous illumination. The strong fluorescence signal registered during the first few nanoseconds following illumination is due to PF, while at longer times after illumination the emitted light is the result of charge recombinations (i.e., DF) (Goltsev et al. 2009). Alternatively, single light pulses of variable length (e.g., 1–200 ms) can be given, during which PF is measured, followed by a dark period during which DF is measured (Schansker et al. 2011). The M-PEA also allows this type of measurement. This approach is much more time-consuming than method (1), because each time point is a separate measurement, but has the advantage that the intensity of the actinic light is also the effective light intensity and, therefore, the results can be related to OJIP transients on a one-to-one basis.

## Question 22: How to compare PF induction transients with induction curves of different DF decay components?

The DF induction curve is often compared to the simultaneously measured PF induction curve. To allow a direct comparison of the maxima for both fluorescence types, they are superimposed on each other on the same timescale (Govindjee and Papageorgiou 1971; Krause and Weis 1991; Malkin et al. 1994; Goltsev et al. 2003, 2005, 2009; Strasser et al. 2010; Kalaji et al. 2012a; Fig. 7a). For the construction of DF induction transients, see Question 21.

During induction, different reactions occur in parallel in different RCs. Upon turning off the light, different reoxidation and recombination reactions all occur separately with their own particular rate constants (for PF see, e.g., Schansker et al. 2005). Since PF is measured during induction (a mix), and DF during dark intervals (in time separated reactions), comparing PF and DF is not straightforward (Mar et al. 1975) and needs an analysis framework.

To understand DF measurements during the first few ms of induction, it is important to understand what happens on the donor side of PSII. The different S states each transfer an electron to Tyr_Z_^+^ with different transfer times: *S*0 → *S*1 = 30 µs, *S*1 → *S*2 = 100 µs, *S*2 → *S*3 = 300 µs, *S*3/4 → *S*0 = 1.5 ms (see Grabolle and Dau 2007 and references therein). This sequence shows that the lifetime of the couple (Tyr_Z_/P680)^+^ increases as a function of the *S* state. As shown by Grabolle and Dau (2005) the fast µs DF component increases on the first three single turnover flashes reaching a maximum intensity on going toward the *S*4 state. These authors also showed for the ms DF component that only the state induced by three flashes (on going from *S*3 via *S*4 to *S*0) still yields a high DF intensity. After a ms, Tyr_Z_^+^ has been re-reduced by the oxygen-evolving complex going to the *S*1, *S*2 and *S*3 states.

According to simulations (Lazár 2003) and actual DF measurements (Schansker et al. 2011, Kalaji et al. 2012a) the maximum DF intensity/maximum [P680^+^] occurs after 3–4 ms (I1 peak) and coincides with the *J* step or slightly after. It is likely that the K peak (in high-temperature treated samples observed after ~300 µs of illumination) is equal to one charge separation and, therefore, the *S*2 state (Tóth et al. 2007a). Simulations of Lazár (2003) suggest that the second charge separation peaks around 1 ms of illumination (*S*3 state). And as mentioned above, the peak DF intensity (*S*4 state) occurs after 3–4 ms. This reaction sequence accounts for the DF rise to the I1 peak.

Using single turnover flashes, several S state cycles can be detected (see Grabolle and Dau 2005 for DF). OJIP transients, on the other hand, are induced by much lower light intensities that are much less concentrated in time. As a consequence the *S* states will dampen (due to double hits and misses; Kok et al. 1970) more quickly. Probably, already shortly after reaching the J step, the donor sides of the PSII RCs will become desynchronized. This means that, at all times, there are 25% *S*0, 25% *S*1, 25% *S*2 and 25% *S*3 and all donor side effects will cancel each other out. Once this has happened the acceptor side of PSII starts to determine DF again. This is observed beyond the J step where an inverse relationship between DF and PF is observed (e.g., Schansker et al. 2011; Kalaji et al. 2012a). Between *J* and *P* the ETC becomes gradually reduced (Schansker et al. 2005). As the PQ pool becomes reduced the availability of oxidized PQ decreases. This means that it takes longer before an oxidized PQ molecule binds to the *Q*_*B*_ site and it takes longer before *Q*_*A*_^−^ can again become re-oxidized. This has two effects: PF increases and DF decreases because only following the re-oxidation of *Q*_*A*_^−^ a new charge separation can occur, and only then there is again a transient induction of P680^+^ (see Schansker et al. 2011, 2014 and Oukarroum et al. 2012 for a more in-depth discussion of this point).

The position of the I1 peak is sensitive to the light intensity. At a relatively low light intensity (500 µmol photons m^−2^ s^−1^; i.e., an effective light intensity of 334 µmol photons m^−2^ s^−1^), the initial DF induction is rather slow and I1 is reached after about 23 ms (Kalaji et al. 2012a, b). Destruction of the Mn cluster changes the DF response. In that case the lifetime of P680^+^ increases and the *Q*_*A*_^−^ concentration strongly decreases (see Oukarroum et al. 2012).

Two DF induction curves are shown in Fig. 7a. They represent different DF decay intervals: 20–90 μs and 0.1–0.99 ms. The characteristic points of the DF induction are indicated according to the nomenclature proposed by Goltsev and Yordanov (1997) and Goltsev et al. (2005, 2009). The maxima (denoted by *I*) and minima (denoted by *D*) are numbered according to their position on the DF induction curve (I1, I2, D2 up to I6; *S* is steady-state DF level).

Similarly to the PF induction curve, the DF induction curve can be divided into fast and slow phases (Itoh et al. 1971; Itoh and Murata 1973; Malkin and Barber 1978; Goltsev and Yordanov 1997; Goltsev et al. 2003, 2005; Fig. 7). The fast phase lasts for about 300 ms and coincides with the OJIP transient of PF. The slow phase lasts several minutes, reaching a stationary level after 5–10 min of illumination by quasi-continuous actinic light. Two maxima I1 and I2 (sometimes with a minimum D1 in between) are observed in the fast phase, after which DF drops to a minimum labeled as D2 (Goltsev and Yordanov 1997; Goltsev et al. 2003; Zaharieva and Goltsev 2003; Kalaji et al. 2012a). The second maximum (I2) appears at about 60–100 ms during the IP phase of PF. I2 strongly depends on the actinic light intensity; at high light intensities it is visible only as a shoulder (Schansker et al. 2011; Kalaji et al. 2012a).

The correlation between simultaneously recorded PF and DF in one sample can be visualized in a “phase diagram” (Malkin et al. 1994; Goltsev et al. 2003; Schansker et al. 2011; Kalaji et al. 2012a). In a 2D graph, DF values collected from different dark delay intervals are plotted as a function of relative double-normalized PF, V_*t*_ (Fig. 7b). For the two plots there is a long induction interval, where the intensities of PF and DF change synchronously (inversely). This appears on the diagram as an almost linear section between phases *S* and *T* of the PF transient. During this time interval both PF and DF intensities are modified, and it has been suggested that this is mainly due to changes in fluorescence quantum yield (Lavorel 1975; Goltsev et al. 2003). In the fast phase of induction (OJIP), PF and DF deviate from linearity for the reasons discussed above. In the first part of the diagram the changes are most pronounced for DF recorded during the 20- to 90-μs decay interval. The dependence between DF and PF is not linear but almost quadratic, which means that PF increases initially much faster than DF. In the time range between points I1 and D2, DF shows a negative correlation with PF—micro- and millisecond DF decreases when PF increases from *J* to *I* and *P*. See above for a discussion of the reasons for this relationship.

Between 0.5 and 5 s DF and PF are again inversely proportional. DF increases from D2 to I4, initially, as a result of the activation of FNR, due to which electron flow restarts (Satoh 1981; Harbinson and Hedley 1993; Schansker et al. 2003, 2008), *Q*_*A*_ starts to turnover again (is re-oxidized and then reduced again), which is accompanied by a transient P680^+^ generation and this leads to an increase of DF emission (and a decrease of PF). The restarted electron flow is also accompanied by a further energetization of the thylakoid membranes. External electric fields are known to stimulate recombination reactions (Vos et al. 1991; Dau and Sauer 1992), and the naturally induced electric field may, therefore, also have an effect on DF emission.

In summary, during induction PF follows the reduction of the electron transport chain, whereas the fast 40-µs and ms DF components are determined by the P680^+^ concentration of the population of PSII RCs, which is determined by the *S* states and the turnover rate of *Q*_*A*_. In other words, both signals monitor the reduction kinetics of the photosynthetic electron transport chain in different ways, providing complementary information.

## Question 23: How do the fluorescence parameters vary during the day?

To produce comparable experimental data several factors are important. In the previous paper the variability among instruments and the extent to which parameters vary naturally was discussed (Kalaji et al. 2014a). With respect to the parameter statistics we want to refer to two additional studies (Lazár and Nauš 1998; Lazár et al. 2005). In the first study it is shown that the statistical distribution of eight OJIP-derived parameters does not follow the Gaussian distribution law, and in the second study it is shown that a stress like high-temperature stress affects the statistical distribution. Changes occurring in leaves during the day and their effect on leaf sampling form another factor. An important variable is the orientation of a leaf. Certain leaves are oriented toward the east and receive most of their daily light dose in the morning, whereas other leaves may be oriented toward the west and receive a large part of their daily light dose in the afternoon. Losciale et al. (2010) and Demmig-Adams et al. (2012) gave experimental examples of this. Another important factor is the angle of the leaf relative to the plane. Leaves that are oriented almost orthogonally will intercept only a relatively small part of the sunlight, whereas leaves oriented nearly parallel to the plane will intercept a large part of the sunlight. A steeper orientation allows a better distribution of the light over different layers of leaves (e.g., Ishida et al. 1999; Falster and Westoby 2003; Stewart et al. 2003). Another factor is wind. Leaves that move around in the wind will continuously change their orientation relative to the sun causing fluctuations in the intercepted light. These fluctuations are further modulated by the passage of clouds and sunflecks. The intensity of sunlight also varies over the day, being low in the early morning and late afternoon and peaking around noon. This natural pattern is responsible for the typical bell curves of parameters like *F*_*V*_/*F*_*M*_ or zeaxanthin + antheraxanthin content during the day (e.g., Demmig-Adams et al. 1996, 2012; García-Plazaola et al. 1997; Bernacchi et al. 2006). In this sense, photosynthetic activity is strongly dependent on the perceived average light intensity, which ultimately drives long-term light acclimation. In shade leaves, having a lower photosynthetic capacity, saturation will be reached at lower light intensities than in sun leaves. It may be noted, on the other hand, that shade leaves exhibit a higher photosynthetic activity at low light intensities and a lower compensation point. Saturation is further modulated by temperature. With the exception of the light reactions (photophysical), photosynthetic electron transport, Calvin–Benson cycle and the photorespiratory pathway have a biochemical character and, as a consequence, photosynthetic activity is quite strongly temperature dependent. A lowering of the temperature will shift the light intensity at which saturation occurs to lower light intensities. Low temperatures associated with high light intensities cause severe winter photoinhibition in Mediterranean (Martínez-Ferri et al. 2004) and Alpine-Central European (Robakowski 2005) tree species. Another important variable is the humidity of the air. Dry air is likely to lead to high levels of evaporation, to which the plant may respond by (partially) closing its stomata (Lange et al. 1971; Schulze 1986). This lowers the uptake of CO_2_ and increases photorespiratory activity at the expense of the assimilatory activity (Medrano et al. 2002). Closing of the stomata will, however, increase the leaf temperature (e.g., Long et al. 2006). All these variables should be considered when designing a leaf sampling protocol for an experiment (Rousseau et al. 2013). Many researchers have experimentally characterized the diurnal pattern of Chl *a* fluorescence parameters in C3, C4 and CAM plants (Adams and Demmig-Adams 1992; Franco et al. 1999; Pollet et al. 2009; Desotgiu et al. 2013). On bright days, solar radiation is supersaturating with respect to the photosynthetic capacity during a considerable part of the day in most plant species and regions of the world (Long and Humphries 1994). Midday high light conditions lead to a decrease in the maximum quantum yield under dark-adapted conditions (*F*_*V*_/*F*_*M*_) and an increase in the rate constant for thermal dissipation reflected by higher levels of *q*_E_ (Gilmore et al. 1998; Adams III and Demmig-Adams 2004). Field observations show the progressive decrease in *F*_*V*_/*F*_*M*_, measured after 20–25 min of dark adaptation, with the lowest values at midday (Desotgiu et al. 2013). An obvious candidate for this decrease is photoinhibition, which causes a loss/quenching of *F*_*V*_ (Long and Humphries 1994). Normally, plants recover from photoinhibition during the night and *F*_*V*_/*F*_*M*_ reaches its maximum value before sunrise (predawn observations) (e.g., Demmig-Adams et al. 2012). Dark acclimation with leaf clips for 20 min during daytime hours ensures the relaxation of the transthylakoid pH difference (Quick and Stitt 1989; Nilkens et al. 2010), inactivation of FNR (Schansker et al. 2006, 2008) and realignment of the chloroplasts within the cell (Cazzaniga et al. 2013; Kong and Wada 2014). Peter Jahn’s zeaxanthin-dependent *q*_*Z*_ quenching, on the other hand, needs considerably more time to recover (Nilkens et al. 2010; Jahns and Holzwarth 2012), just as the release of part of the PSII antenna proposed by Alfred Holzwarth (Holzwarth et al. 2009) and Roberto Bassi (Betterle et al. 2009), and even more time is needed to recover from photoinhibition (Horton and Hague 1988). These last three processes are all associated with a sustained *F*_*V*_/*F*_*M*_ reduction.

Photosynthesis frequently remains depressed during the afternoon hours (Correia et al. 1990; Pollastrini et al. 2013). Apart from photoinhibition and slowly reversing regulatory mechanisms, abscisic acid-induced stomatal closure in the afternoon (Tallman 2004) and feedback inhibition of photosynthesis by accumulating sucrose and/or starch have been considered (see Paul and Foyer 2001 for a discussion). It is worth noting that nighttime recovery of photoinhibition may be inhibited or slowed down by low night temperatures (Strand and Lundmark 1987; Bussotti 2004). The temperature dependence of the repair cycle and its inhibition under low temperature conditions has been described under in vitro conditions (Aro et al. 1990).

The parameter 1 − *V*_*J*_, which is an approximation of the relative amplitude of the thermal phase, follows the same diurnal pattern as the parameter *F*_*V*_/*F*_*M*_ (Desotgiu et al. 2013; Pollastrini et al. 2011, 2014). In the JIP test this parameter is associated with electron transport activity (Strasser et al. 2004); however, there are several processes to which this parameter is sensitive, including severe high-temperature stress associated with inactivation of the PSII donor side (Schreiber and Neubauer 1987; Srivastava et al. 1997; Tóth et al. 2007a), and a partially reduced PQ pool (Tóth et al. 2007b), but also certain regulatory mechanisms (Schreiber et al. 1995; Schansker et al. 2006). Photoinhibition, reducing the donation capacity of all PSII RCs together, may, in this respect, have the same effect on OJIP transient as high-temperature stress. The water status of plants may play an important role. Desotgiu et al. (2012a) observed for well-watered plants of *Fagus sylvatica* L. that 1 − *V*_*J*_ increased at midday. The amplitude of the IP phase, which was shown experimentally to correlate with leaf PSI content in fully dark-adapted leaves, was always found to be enhanced during the noontime hours as well as in sun leaves (Cascio et al. 2010; Desotgiu et al. 2013; Pollastrini et al. 2014). However, the reason for this observation is different in these two cases. In dark-acclimated sun leaves the PSII/PSI ratio may be lower than in dark-acclimated shade leaves (Anderson et al. 1988) and this is expected to lead to an increased IP amplitude. If leaves suffer from a significant amount of photoinhibition at noontime, the electron donation capacity of PSII will be lower. This is comparable to illumination at a lower light intensity (cf. Schansker et al. 2011) or high-temperature stress (cf. Srivastava et al. 1997), both conditions that increase the relative amplitude of the IP phase. The electron transport rate (ETR), the effective quantum yield (Φ_PSII_) and photochemical quenching (*q*_P_) derived from PAM fluorimetry on light-adapted samples are higher during the day than at night (Larcher 2000; Pollet et al. 2009; Desotgiu et al. 2013). An explanation for this observation may be that leaves kept overnight in darkness may have lower sink activities and a reduced stomatal opening (Felle et al. 2000) compared to plants that have been exposed for hours to ~600 µmol photons m^−2^ s^−1^.

## Question 24: How do fluorescence parameters vary within a tree canopy?

Leaves from a tree form a population, and each single leaf may differ from the others in terms of size, age and position in the tree canopy. The position of the leaf in the canopy (top vs. bottom leaves; outer vs. inner) determines primarily the exposure to sunlight and the differentiation between sun and shade leaves. Sun and shade leaves differ significantly in their photosynthetic apparatus and performance (Lichtenthaler et al. 1981; Anderson et al. 1988). Sun leaves have in general a lower Chl *a* and *b* content, smaller thylakoid stacks, lower LHCII content, higher Chl *a*/*b* ratio, lower Chl(*a* + *b*)/carotenoid ratio and a higher PSI/PSII ratio when compared with shade leaves. Furthermore, sun leaves have a higher Calvin–Benson cycle capacity relative to the capacity of the electron transport chains, and more efficiently dissipate excess energy as heat, compared to shade leaves. Shade leaves, on the other hand, are more efficient at exploiting low PAR levels for photosynthesis, having a larger PSII antenna and more extensively stacked thylakoid membranes (Lichtenthaler et al. 1981).

The Chl *a* fluorescence parameters most sensitive to sunlight exposure during the day are (1) the maximum quantum yield of primary photochemistry (*F*_*V*_/*F*_*M*_) as a measure for the efficiency of the whole PSII population and the sensitivity of PSII to photoinhibition, and (2) non-photochemical quenching (NPQ). NPQ induction in response to solar radiation is stronger in sun leaves than in shade leaves. Among the chlorophyll fluorescence parameters derived from OJIP transients, the amplitude of the IP phase, that reflects the relative PSI content (Oukarroum et al. 2009; Ceppi et al. 2012), is higher in sun leaves than in shade leaves, which is in agreement with the higher PSI/PSII ratio.

The Chl *a* fluorescence parameters are also sensitive to the age and/or senescence of leaves/needles in evergreen tree species and to the age of leaves appearing in the spring compared with those developed during the summer or autumn (Desotgiu et al. 2012b).

Some stress factors act preferentially on a specific side of the canopy. These factors include, e.g., wind blowing dominantly out of a particular direction and provoking desiccation of leaves, salt from the sea shore, and chemicals from local pollution sources. Other environmental factors, such as soil properties and air pollutants, affect the canopy in a different way. The effects of these factors depend on the physiology, ontogeny and the position of leaves in the crown. We can thus have damaged leaves at specific levels in the tree crown, e.g., ozone symptoms on leaves in the lower part of the crown, but not in the higher part (Desotgiu et al. 2012b).

Finally, when leaves are lost from the branches due to senescence, or damaged by biotic or abiotic factors, the photosynthetic activity of the remaining foliage may increase (Eyles et al. 2011). Such a response affects the value of several Chl *a* fluorescence parameters, especially the amplitude of the IP phase of the OJIP transient (Desotgiu et al. 2012b).

## Question 25: What do Chl *a* fluorescence measurements tell us about drought stress?

The usefulness of individual Chl *a* fluorescence parameters and protocols for evaluation of drought depends on the severity and duration of drought stress (Suresh et al. 2012). Mild-to-moderate drought stress causes a decrease in the photosynthetic rate, mainly due to stomatal closure, whereas drought has no direct effect on the capacity of individual metabolic reactions (Brestič et al. 1995; Cornic and Massacci 1996; Flexas and Medrano 2002). Recently, it was observed that blue-light-induced chloroplast movements are very sensitive to drought stress. Inhibition occurred already at RWC values considerably above 70%, and was, therefore proposed to be a sensitive tool for small changes in RWC (Nauš et al. 2016). In typical C3 plants, critical leaf relative water content is about 70% and below this value non-stomatal effects occur. These phenomena are also reflected in Chl *a* fluorescence and calculated fluorescence parameters.

**Fig. 8:**
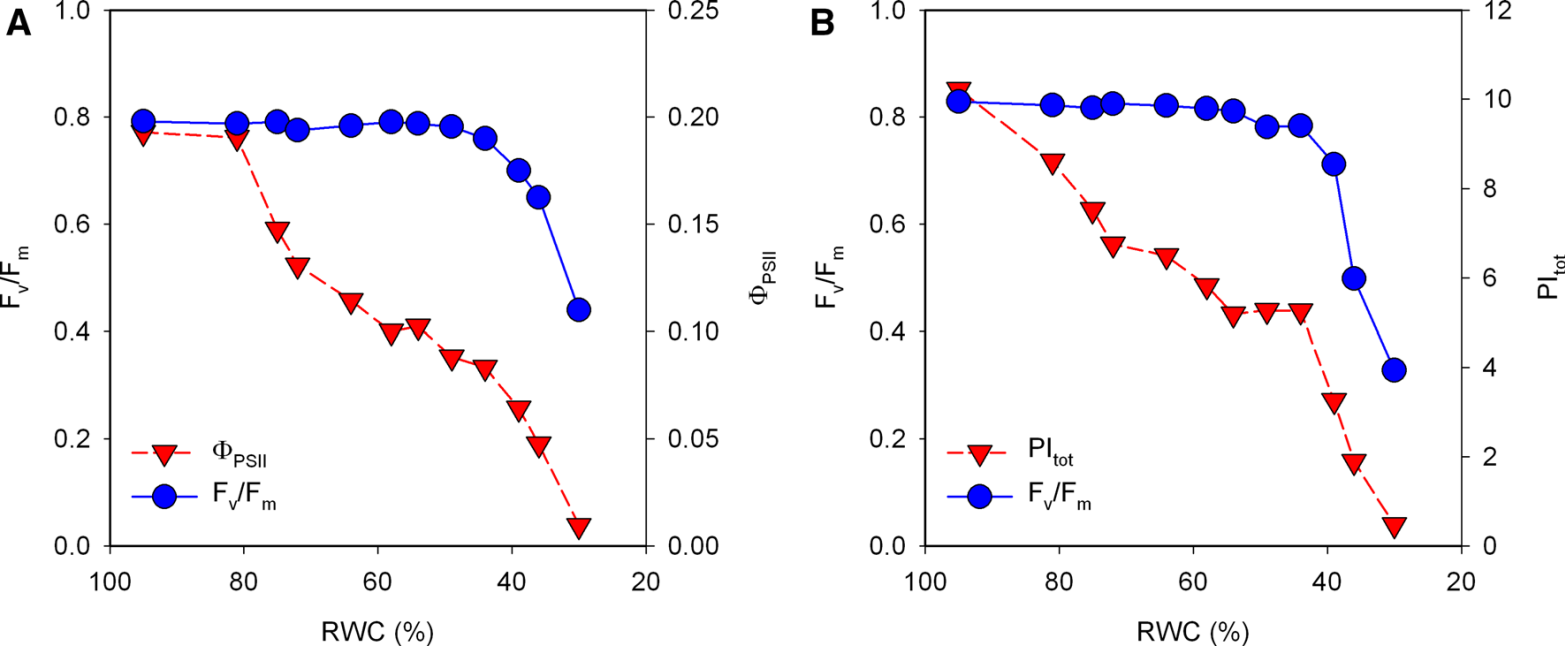
Response of Chl *a* fluorescence parameters to drought stress. **a** The parameters *F*_*V*_/*F*_*M*_ and Φ_PSII_ measured with a PAM fluorometer as a function of the relative water content (RWC); **b** the parameters *F*_*V*_/*F*_*M*_ and *PI*_tot_ measured with a HandyPEA as a function of the RWC (modified from Brestič and Živčák, Molecular Stress Physiology of Plants, Chapter 4, republished with permission of Springer)

The most frequently used fluorescence parameter in photosynthetic or environmental research (including drought stress) is the maximum quantum yield of PSII photochemistry (*F*_*V*_/*F*_*M*_). This parameter is easy to measure and is generally well accepted as a measure for the PSII status (or more correctly the status of the population of PSII RCs). However, it is highly insensitive to stomatal changes and other effects occurring under moderate drought stress (see Fig. 8, recorded during quick dehydration of a wheat leaf in very low light). *F*_*V*_/*F*_*M*_ values are extremely stable, starting to decline at a dehydration level that is lethal for typical leaves. If drought stress persists under field conditions for a longer period (days) the decrease in *F*_*V*_/*F*_*M*_ values can be dramatic (Živčák et al. 2008b). Hence, decreases in *F*_*V*_/*F*_*M*_ cannot be used to monitor early drought stress effects. However, the *F*_*V*_/*F*_*M*_ measurements during drought stress may draw attention to the effects of co-occurring stresses (high-temperature stress, photoinhibition, etc.) or to leaf senescence (Lu et al. 2002; Kotakis et al. 2014).

Both the slow and fast Chl *a* fluorescence kinetics provide parameters sensitive to drought stress (Fracheboud and Leipner 2003; Oukarroum et al. 2007, 2009; Živčák et al. 2008a, 2014; Goltsev et al. 2012 ). The early decrease of the effective PSII quantum yield (Φ_PSII_) in drought-stressed leaves compared to well-hydrated ones is mainly due to lack of CO_2_ inside the leaf (closed stomata). In C3 leaves, the decrease of Φ_PSII_ is not linearly correlated with net photosynthetic rate, as increased photorespiration efficiently consumes part of the electrons flowing through the photosynthetic electron transport chain. Moreover, alternative electron transport pathways also consume electrons generated by linear electron transport under drought stress conditions, complicating the physiological interpretation of the results (Živčák et al. 2013; Questions 11 and 13). For example, in C3 plants PSII activity (O_2_ evolution rate) relative to Rubisco activity is ~equal to the carboxylase activity + the oxygenase activity which in turn correlates with PSII activity measured by Chl *a* fluorescence analysis (Krall and Edwards 1992; Oberhuber et al. 1993).

In summary, measurements of slow fluorescence kinetics and calculation of quantum yields and electron transport rate (ETR) are useful for determination of drought stress effects, reflecting both stomatal and non-stomatal effects. However, such measurements during drought stress cannot be directly related to CO_2_ assimilation (Baker 2008). Indeed, the relative fluorescence decrease ratio (*R*_Fd_) was shown to be more sensitive and better correlated with the photosynthetic CO_2_ assimilation rate than PSII quantum yield or ETR (Lichtenthaler et al. 2005a). *R*_Fd_ was introduced as a so-called vitality index, calculated as *R*_Fd_ = (*F*_*P*_ − *F*_*S*_)/*F*_*S*_, where *F*_*P*_ and *F*_*S*_ denote the fluorescence intensities at the fluorescence peak after 200–500 ms of illumination (*F*_*P*_), i.e., *P* of the OJIP transient, and the fluorescence level in the steady state (*F*_*S*_) reached after a few minutes (usually 2–5 min) of illumination. When a plant is stressed, *F*_*P*_ decreases due to processes such as photoinhibition and under stress conditions *F*_*S*_ can also increase, leading to a decrease of *R*_Fd_ (Lichtenthaler et al. 1986). Lichtenthaler (2013) showed that *R*_Fd_ is sensitive to the acclimation state of leaves (sun–blue-shade–half-shade–shade) and correlates roughly with *P*_n_, and there is a strong correlation with *F*_*V*_/*F*_*O*_. The fluorescence decrease from *F*_*P*_ to *F*_*S*_ depends on several factors that have not yet been characterized completely, complicating the physiological interpretation of *R*_fd_ values (Roháček 2002).

Recently, fast Chl *a* fluorescence kinetics and the JIP test (Strasser et al. 2000) have become popular for rapid screening of stress effects. The measurements are as simple as *F*_*V*_/*F*_*M*_ measurements and provide additional information on the photochemistry of PSII and the photosynthetic electron transport chain. Figure 8 shows the comparison of the response of three parameters [*F*_*V*_/*F*_*M*_ measured by the PAM (Fig. 8a) and HandyPEA (Fig. 8b), Φ_PSII_ (Fig. 8a) and the Performance Index (*PI*_tot_) (Fig. 8b)] to decreasing leaf water content (RWC) in wheat. The *F*_*V*_/*F*_*M*_ level is quite insensitive to a decrease in RWC, whereas Φ_PSII_ and *PI*_tot_ respond strongly to a RWC decrease, showing a similar dependence on RWC. Similar decreases have been observed under natural conditions during slowly advancing drought stress (Živčák et al. 2008b). The decrease in the Performance Index is associated with changes in Chl *a* fluorescence transients and, by extension, changes in rate constants of individual electron transport steps and/or the state of the ETC that are detected by fast Chl *a* fluorescence induction (reviewed, e.g., by Lazár 1999; Strasser et al. 2004). The simplicity of the fast Chl *a* measurements and automated analysis have sometimes led to misapplication. Analysis should be supported by parallel measurements using other methods, and the restrictions of the JIP test analysis should be kept in mind (is the *F*_*M*_ level still reached, is the *F*_*O*_ truly measured, is the electron transport chain fully reoxidized?).

## Question 26: Can Chl *a* fluorescence be used for high-temperature stress tolerance comparisons?

Photosynthesis is very sensitive to high temperatures and can be partially or even completely inhibited before other stress symptoms are observed. High temperatures affect photosynthesis by their effect on the rates of chemical reactions and on the structural organization of the photosynthetic apparatus (Pastenes and Horton 1996).

Photosynthetic CO_2_ assimilation decreases at moderately high temperature levels (up to 38 °C) due to thermal inactivation of Rubisco activase, the enzyme that removes inhibiting molecules from the active site of Rubisco (Feller et al. 1998; Law and Crafts-Brandner 1999; Salvucci and Crafts-Brandner 2004). This decrease is reversible and leads to a decrease in the linear electron transport rate, which can be detected using the saturating pulse method.

Direct impairment of PSII occurs when the leaf temperature reaches ~40 °C and higher. This may be due to high-temperature-induced changes in the properties of thylakoid membranes (Sharkey and Zhang 2010; Yamauchi and Sugimoto 2010), the dissociation of the manganese-stabilizing protein from the PSII reaction center complex and the release of Mn atoms (Yamane et al. 1998). The Mn cluster can be reconstituted (Ananyev and Dismukes 1996 and references therein); however, in the leaf this does not seem to happen and once the Mn cluster has fallen apart, PSII follows the same repair cycle as in the case of photoinhibition (Tóth et al. 2005b; Komayama et al. 2007).

Damage to PSII can be observed in slow Chl *a* fluorescence kinetics using the saturating pulse method (e.g., Pastenes and Horton 1996), but the most efficient way is through measurements taken after a period of dark acclimation. The high-temperature effects on PSII photochemistry and high-temperature tolerance at the PSII level have frequently been characterized in terms of *F*_*O*_ increases or *F*_*M*_ decreases. However, the basal and maximum fluorescence values are rather variable between different samples, even under non-stressed conditions, and their use can, therefore, become a source of uncertainty.

The maximum quantum yield, *F*_*V*_/*F*_*M*_, is the most frequently used measure of direct high-temperature effects on PSII. This parameter is based on the assumption that *F*_*O*_ is measured for open RCs (*Q*_*A*_ fully oxidized) and *F*_*M*_ for closed RCs (*Q*_*A*_ fully reduced). Under high-temperature conditions, *F*_*O*_ can slightly increase as high temperatures enhance the process of chlororespiration, leading to a partial reduction of *Q*_*A*_ in the dark due to a more reduced PQ pool (Sazanov et al. 1998). In high-temperature-stressed samples with a large population of PSII with an impaired oxygen-evolving complex (OEC), electron transport rate is lower and it takes longer to reach the maximum fluorescence intensity. In high-temperature treated samples, FNR may be activated before *F*_*M*_ is reached and then the measured *F*_*M*_ does not relate to a fully reduced ETC—a prerequisite for *F*_*M*_ determination under non-inhibited conditions—causing an underestimation of *F*_*M*_ (Tóth et al. 2005a, 2007a). Both the overestimation of *F*_*O*_ and underestimation of *F*_*M*_ lead to *F*_*V*_/*F*_*M*_ underestimation and therefore overestimate the temperature effect. In addition, the observed change in the *F*_*V*_/*F*_*M*_ value is related to a loss of electron donation capacity and not to a change in the PSII quantum yield.

**Fig. 9:**
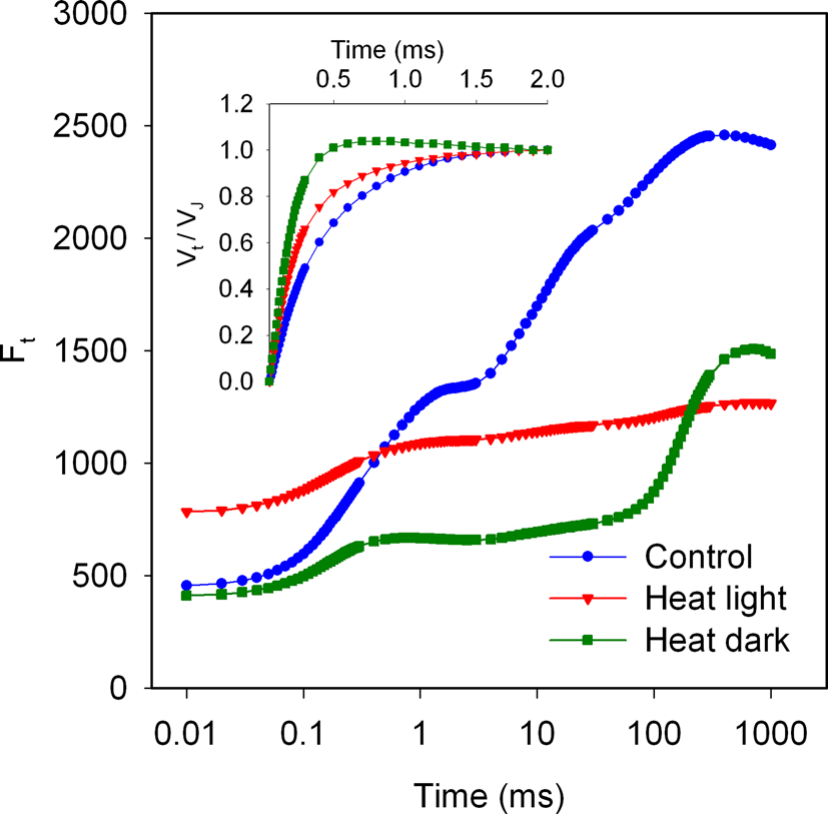
Comparison of the effect of high-temperature stress applied in the light and in darkness on the OJIP kinetics. In the inset the Chl *a* fluorescence transients double-normalized between *O* and *J* are shown (Živčák and Brestič, unpublished data)

Rate constants or some parameters derived from fast Chl *a* fluorescence induction show a greater sensitivity to high temperatures than conventional fluorescence parameters such as *F*_*V*_/*F*_*M*_. The typical “visual” symptom of PSII high-temperature injury is the appearance of a new peak within the fluorescence induction curve at approximately 300 µs (denoted as the K step). This is frequently accompanied by a slowdown of the *J*–*I* rise and by an increase in the amplitude of the *I*–*P* rise (Fig. 9). The K step appears as a consequence of a high-temperature-induced destruction of the Mn cluster in a considerable fraction of PSII RCs (Srivastava et al. 1997; Tóth et al. 2005a).

**Fig. 10:**
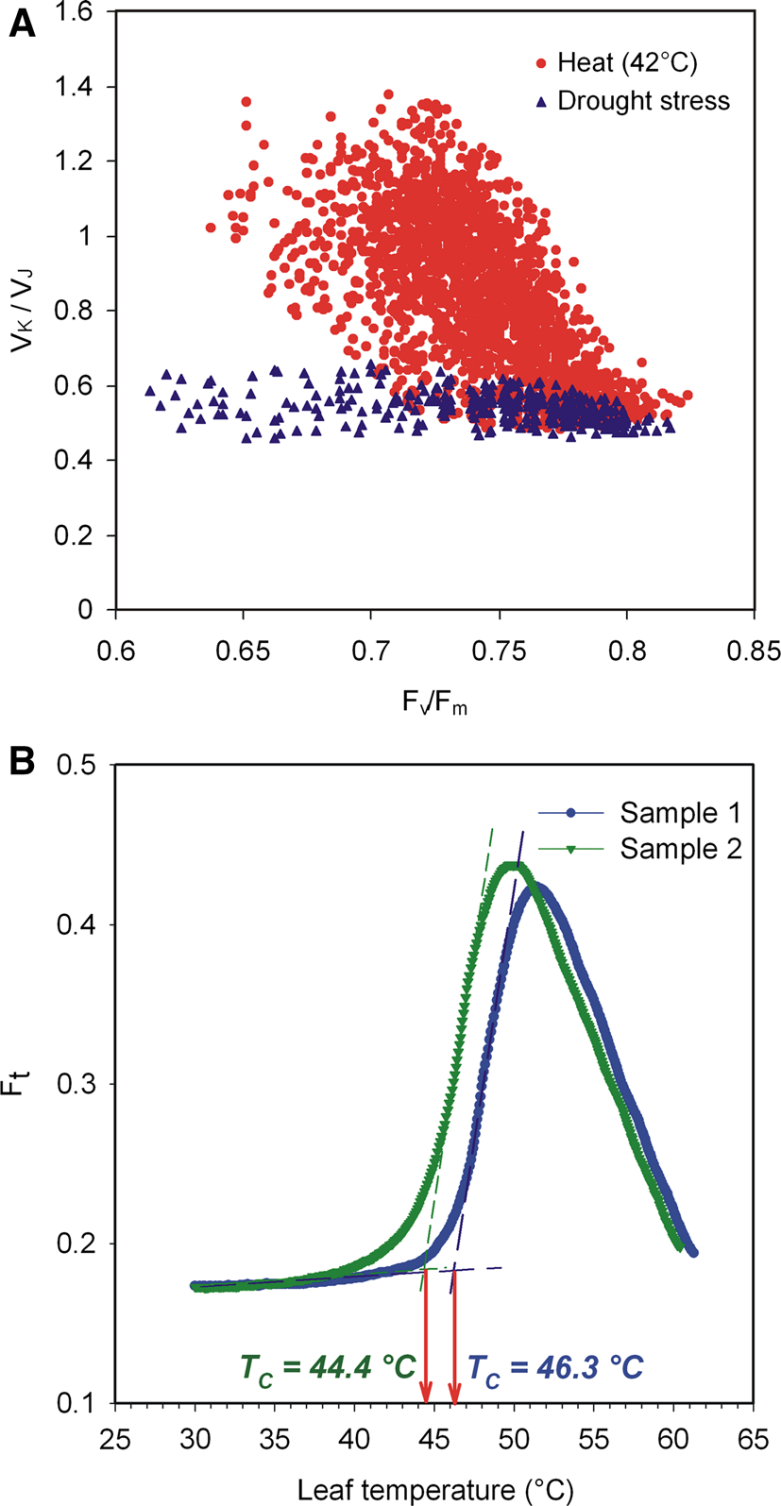
High-temperature stress effects. **a** The parameter *V*_*K*_/*V*_*J*_ as a function of the parameter *F*_*V*_/*F*_*M*_ used here as a measure for the severity of the treatment. Heat stress induces a significant increase in the value of the parameter *V*_*K*_/*V*_*J*_, whereas drought stress has no effect on this parameter. **b**. Demonstration of the critical temperature (*T*_C_): gradually increasing the leaf temperature leads above a certain critical temperature to an increase of the *F*_*O*_ value. *T*_C_ is determined as indicated in **b**; it differs between photosynthetic samples and may be used as an indicator for adaptation or tolerance to heat (Živčák and Brestič, unpublished data)

The *K* step has only been described in response to high-temperature stress or manganese deficiency, and its occurrence is thus a very specific symptom of high-temperature-induced damage of PSII. This is clearly illustrated in Fig. 10a, where *V*_*K*_/*V*_*J*_ values derived from fluorescence kinetics recorded on wheat plants subjected to progressive drought stress and a high-temperature treatment are shown. It is evident that even very strong drought stress did not induce any increase in *V*_*K*_/*V*_*J*_, whereas short periods of high temperatures caused substantial increases in this parameter. High-temperature stress responses vary considerably when data from a large collection of wheat genotypes of diverse provenance are used (Brestič et al. 2012).

The *K* step is much more apparent if the leaves are heated in the dark (Fig. 9), since light causes a constant oxidation of the Mn cluster and therefore prevents the super-reduction of the Mn cluster, which is responsible for the disintegration of the Mn cluster in the absence of extrinsic proteins (e.g., Beck and Brudvig 1987). As a consequence, the *K* peak is much lower under such conditions (Fig. 9). RCs that lack a Mn cluster are very sensitive to light (e.g., Blubaugh and Cheniae 1990) and, therefore, quickly become photodamaged. High-temperature susceptibility of PSII differs depending on species, age, physiological status, acclimation level, etc. The ability of PSII to tolerate high-temperature stress is termed “PSII thermostability” and is clearly demonstrated by the dependence of basal fluorescence on temperature (*F*_*O*_-T curve), introduced by Schreiber and Berry (1977). The *F*_*O*_-T curve method is based on a continuous increase of sample temperature during which the *F*_*O*_ value is continuously recorded. The critical temperature *T*_C_ represents the temperature at which *F*_*O*_ starts to increase steeply (Fig. 10b). As shown by Ducruet (1999), this increase is due to a shift in the (*Q*_*A*_ *Q*_*B*_)^−^ equilibrium toward *Q*_*A*_. The critical temperature for different species may vary considerably, with the critical temperature ranging from 42 °C up to more than 50 °C (Froux et al. 2004). It is known that increasing the saturation level of membrane lipids increases the tolerance to high temperatures (Murakami et al. 2000) and differences in this respect between plant species may explain in part the observed variability.

Analogous to the continuous *F*_*O*_ measurements, the exposure of fresh leaf samples to a single temperature (the graduated temperature approach) (Živčák et al. 2008c; Brestič and Živčák 2013) provides more detailed information about high-temperature effects on PSII photochemistry and enables the estimation of the *T*_C_ for *F*_*O*_. Critical temperatures represent the point at which a severe disorganization of structure and loss of main functions occur. Hence, the estimated critical temperature can occur above the “physiologically relevant” temperature range, reaching temperatures as high as 50 °C. This estimate is non-physiological, as in most cases leaves in a field are not typically exposed to 50 °C or more. On the other hand, the use of graduated temperatures with fast Chl *a* fluorescence not only gives a real-life *T*_C_ measurement, but also enables the calculation of other parameters. A broad study on 30 field-grown wheat genotypes demonstrated that the *K* step occurred even at temperatures 4–5 °C lower than the steep *F*_*O*_ increase (Brestič et al. 2012). Moreover, the capacity to increase thermostability of the K step was higher than for the *F*_*O*_ increase. High-temperature acclimation can occur quite quickly. Lazár et al. (1997a, b) observed a shift of the temperature at which the K peak appeared (*T*_K_) by 3 °C (from 47 to 50 °C) following the incubation of barley leaves for 2–3 h at 35 °C. Using possibly the same acclimation data set Lazár and Ilík (1997) observed an approximately linear relationship between *T*_C_ and *T*_K_. However, *T*_K_ increased more than *T*_C_ following acclimation and *T*_K_ was always higher than *T*_C_. This contradicts somewhat the above-mentioned observation of Brestič et al. (2012).

PSII thermostability is only a small part of overall high-temperature tolerance. The reversible inactivation of Rubisco activase occurs already at considerably lower temperatures than PSII inactivation. However, inactivation of the donor side of PSII takes considerably longer to repair than re-activation of Rubisco activase. As a consequence, PSII donor side inactivation may not so much play a role during a high-temperature episode; instead, it may have an effect on plant photosynthetic productivity in its aftermath. In any case, the application of fast Chl *a* fluorescence measurements with *F*_*O*_, *F*_*V*_/*F*_*M*_ along with the K step determination appears to be an efficient approach for screening PSII thermostability, enabling rapid identification of high-temperature-resistant or high-temperature-sensitive samples.

## Question 27: Can variable Chl *a* fluorescence be used as a biomarker of environmental pollution?

By using Chl *a* fluorescence it is possible to detect the effects of environmental stressors (e.g., herbicides, ozone, trifluoroacetic acid, acid rain, many heavy metals) that disrupt photosynthesis (Judy et al. 1991; Lewis et al. 2001; Guidi et al. 2010; Chaudhary et al. 2013). Chl *a* fluorescence-based methods have been applied in ecotoxicological studies to examine the effects of pollutants on algae and plants (Kumar et al. 2014). These methods have many advantages over existing bioassays, especially with regard to sensitivity, rapidity, and the non-destructiveness and non-invasiveness of the methodology. Depending on the stressor, changes can be detected before visible symptoms appear (Guidi et al. 1997, 2000; Popovic et al. 2003).

While it is commonly assumed that parameters linked to PSII electron transport are indicators for toxicity in plants (e.g., Perreault et al. 2010), it is important to specify which kind of parameter could be a good biomarker for a particular agent. For instance, in *Lemna* plants exposed to copper oxide nanoparticles, the effective PSII quantum yield (Φ_PSII_) is a reliable indicator for toxicity after 24 h of exposition, but *F*_*V*_/*F*_*M*_ remains unaffected (Perreault et al. 2010). However, upon long exposure of *Lemna* to a heavy metal such as Cr, *F*_*V*_/*F*_*M*_ and *F*_V_/*F*_*O*_ showed a clear dose-dependent decrease (Reale et al. 2016). Similarly, *F*_*V*_/*F*_*M*_ can be used for the detection of several herbicides that inhibit linear electron transport (see Question 28) and petrochemicals that can contaminate natural waterways or reservoirs (Conrad et al. 1993; Ralph and Burchett 1998; Dorigo and Leboulanger 2001; Choi et al. 2012). The effective quantum yield has been found to be a sensitive indicator for certain heavy metals, herbicides and petrochemicals (Ralph and Burchet 1998; Haynes et al. 2000; Juneau et al. 2001; Marwood et al. 2001; Macinnis-Ng and Ralph 2003; Perreault et al. 2010; Wilson and Ralph 2012).

The tolerance to pollutants differs between plant species. Uptake reduction, compartmentalization or differential detoxification are strategies that have been demonstrated in plants (Hall 2002). For every bioassay it is, therefore, important to choose plant or algal species that are sensitive to the pollutant or mixture of pollutants of interest. In the case of crops, the comparison of sensitive and tolerant cultivars can also be interesting (e.g., Calatayud et al. 2002; Degl’Innocenti et al. 2002).

In summary, for a successful bioassay, the choice of a species sensitive to the pollutant and of a Chl *a* fluorescence parameter that is affected by the pollutant in the concentration range of interest is critical (Choi et al. 2012). Taking these considerations into account, Chl *a* fluorescence methods represent a more rapid and sensitive methodology than growth assays (Kvíderová 2010; Fai et al. 2007).

## Question 28: Can toxicity induced by different herbicides be identified?

Screening for herbicide efficacy and plant sensitivity is usually a lengthy process. The experiments take up a lot of space, and the evaluation of results, either quantitatively or qualitatively, is normally completed more than a week after the treatment (Christensen et al. 2003). In this respect, the most straightforward category of herbicides is those that bind to the *Q*_*B*_ site of PSII (e.g., DCMU, atrazine and phenolic herbicides like ioxynil or bromoxynil). Full inhibition of PSII by these herbicides raises *F*_*J*_ to the *F*_*M*_ level, and the percentage of increase between *F*_*J*_ and *F*_*M*_ can be easily quantified (cf. Lazár et al. 1997a, 1997b, 1998; Tóth et al. 2005b). However, as shown by Krieger-Liszkay and Rutherford (1998), the effect of these herbicides on the midpoint potential of *Q*_*A*_ affects their working mechanism. DCMU increases the midpoint potential of *Q*_*A*_, stabilizing the charge separation, whereas phenolic herbicides decrease the midpoint potential of *Q*_*A*_ and destabilize the charge recombination (see also Question 13). The first type of herbicides reduces the probability that a charge recombination induces singlet oxygen, whereas the second type increases this probability. In other words, the extent of inhibition is only half the story.

The activities of a surprisingly large number of herbicides are directly or indirectly influenced by light (Hess 2000). Herbicides that catalyze the formation of reactive oxygen species (e.g., paraquat and diquat), block the synthesis of carotenoids directly or indirectly, inhibit protoporphyrinogen oxidase during Chl biosynthesis or inhibit glutamine synthetase in the nitrogen assimilation pathway can, therefore, also be detected using fluorometric methods (Fuerst et al. 1985; Kaňa et al. 2004; Merkell et al. 2004; Søbye et al. 2011). Paraquat, also known as methylviologen, can accept electrons from the FeS clusters of PSI in competition with Fd. In dark-adapted leaves this is observed as a suppression of the IP phase of the OJIP transient (e.g., Schansker et al. 2005). Herbicides binding to the *Q*_*B*_ site do not necessarily affect the *F*_*O*_ or *F*_*M*_ values (Tóth et al. 2005b). However, the measuring light of a PAM instrument will strongly increase the measured *F*_*O*_ value in samples in which the re-oxidation of *Q*_*A*_^−^ is fully or partially inhibited. This effect decreases the calculated *F*_*V*_/*F*_*M*_ value and allows the detection of such herbicides (Bolhar-Nordenkampf et al. 1989).

Glyphosate application leads to a depletion of free phosphate, leading to an inhibition of ATP synthesis, and isoxaflutole inhibits PQ synthesis. Søbye et al. (2011) showed that *F*_*J*_ and the slopes at the *J* and *I* steps can be used to titrate the effects of the herbicides glyphosate and isoxaflutole as well as mixtures of these two compounds. However, looking at the OJIP transients shown in that paper, these parameters seem to reflect mainly the destruction of either the photosynthetic system or alternatively PSII, since the main effect was observed on the *F*_*V*_ amplitude. The authors note that the fluorescence data obtained 48 h after the treatment gave the same information as the biomass measurements carried out 3 weeks after the treatment.

Special equipment for the detection of herbicides in microalgae was introduced by the laboratries of Jean-Claude Duval and Claudia Büchel (Arsalane et al. 1993; Conrad et al. 1993). The principle is the same as that for the PAM and *F*_*V*_/*F*_*M*_ mentioned above, but the equipment was considerably more sensitive than the PAM. The equipment works with low-intensity-modulated light that does not lead to the induction of fluorescence in the absence of herbicide, leads to the induction of fluorescence as a function of the fraction of PSII RCs inhibited by herbicide.

Herbicides that inhibit the synthesis of amino acids or lipids have indirect effects on photosynthesis through their effect on the carbon metabolism or the stability of thylakoid membranes and may consequently alter PSII and PSI photochemical activity. Their effects on the fluorescence kinetics will only be apparent after a much longer exposure time than in the case of PSII-type inhibitor herbicides (Popovic et al. 2003). Olesen and Cedergreen (2010) and Yanniccari et al. (2012) have argued that the inhibition of CO_2_ assimilation is a better and more direct probe for glyphosate effects than Chl *a* fluorescence. This is true for OJIP transients, but it can be pointed out that a full Kautsky curve measured till the steady state is reached would also give information on the inhibition of CO_2_ assimilation. As noted by Yanniccari et al. (2012), the parameter ETR (a parameter determined for steady-state conditions) is a good probe for herbicides that have a kinetic effect on photosynthetic activity since fluorescence measurements are easier and faster than quantification of CO_2_ assimilation by IRGA.

Chl *a* fluorescence imaging does not only allow the measurement of a whole plant or several small plants simultaneously, and it also makes it possible to follow the spread of herbicides in leaves or whole plants (see Lichtenthaler et al. 2005b). Herbicide-induced perturbations of plant metabolism have been detected using changes in the derived images of fluorescence parameters before any visual effects on growth were observed (Barbagallo et al. 2003). Konishi et al. (2009) made a three-dimensional analysis of the uptake of DCMU, in plants of *Cucumis melo*, in which the third dimension was the uptake time. This allowed the authors to show in detail how the herbicide, arriving in the leaf via the xylem, spread inside the leaf. Saura and Quiles (2009), using Chl *a* fluorescence imaging, to compare the uptake of DCMU and paraquat in *Chrysanthemum morifolium, Rosa meillandina* and *Spathiphyllum wallisii*, showed (1) that paraquat, an herbicide acting on the acceptor side of PSI, can also be monitored by Chl *a* fluorescence imaging and (2) that the more water-soluble paraquat affected the leaves more homogeneously than DCMU. Muller et al. (2008) showed how a combination of the Maxi imaging PAM, black 96 well plates and *Chlorella vulgaris* and *Phaeodactylum tricornutum* as biosensors can be used as a rapid and inexpensive bioassay for herbicides in, e.g., water samples.

Chl *a* fluorescence imaging has not only been used to detect the effects of herbicides on the photosynthetic performance of plants but also of algae as recently reviewed by Kumar et al. (2014).

## Question 29: Can fluorescence parameters be used for QTL studies?

QTLs or quantitative trait loci refer to a location on a chromosome coding for one or more genes that affect a certain characteristic or process, e.g., stress tolerance or sensitivity. It is likely that, e.g., drought stress tolerance is affected by many genes that can be found on several chromosomes. The word quantitative refers to the fact that the genes linked to a QTL only have partial control over a characteristic. With respect to photosynthesis there is one more peculiarity. QTLs are located in the nuclear genome, whereas many important photosynthetic genes are found in the chloroplast genome.

Several studies have been carried out that had as goal to identify QTLs related to fluorescence parameters (see Question 30 for a discussion of a rational choice of parameters for such studies). There are two main approaches to the identification of QTLs, but for the study of QTLs related to fluorescence parameters only one of them, association mapping, has been used (see Flood et al. 2011 for a discussion of linkage mapping). Czyczyło-Mysza et al. (2013) used for their study 94 daughter lines from the cross of two wheat varieties: Chinese spring × SQ1. For these daughter lines the authors determined the following parameters: *F*_*V*_/*F*_*M*_, *PI*_abs_ (performance index), ABS/CSm, TRo/CSm, ETo/CSm, DIo/CSm, RC/CSm, Chl *a* + *b*, SPAD, Car, DWP (dry weight per plant), GWE (grain yield of the main stem), YP (grain yield per plant).

Separately, a genetic map was made based on the DNA of 90 lines. This resulted in a Chinese spring × SQ1 genetic map defined by 1039 genetic markers of which 472 were derived from their study. In addition, 165 genes related to the photosynthetic light reactions, pigment metabolism.

The authors then tested, which of the studied parameters were linked, in the sense that they were inherited together in the different daughter lines. By studying the extent of linkage between the parameters and the genetic markers, the genes controlling the variability in the studied parameters could be assigned to areas on particular chromosomes. Czyczyło-Mysza et al. (2013) observed that the productivity traits were not consistently correlated with any of the fluorescence or pigment traits.

Similar studies were carried out by several other groups. Stamp and coworkers published several QTL studies related to chilling tolerance in maize. Fracheboud et al. (2002) studied 233 recombinant inbred lines derived from a cross between a drought-tolerant and a drought-sensitive maize variety grown at 15 and 25 °C. The authors screened the following parameters: CO_2_ assimilation, Φ_PSII_, *F*_*V*_/*F*_*M*_, *F*_*O*_ (rel.), stomatal resistance, Chl *a* × *b*, Chl *a*/*b*, β-carotene/lutein and different carotenes/xanthophylls. In a second study, Fracheboud et al. (2004) screened: *F*_*O*_, *F*_*V*_/*F*_*M*_, Φ_PSII_, *F*_*V*_′/*F*_*M*_′, *q*_P_, CER (CO_2_ assimilation), SPAD, shoot DW, *N* (%) (nitrogen content). Apart from an assignment of several QTLs, these studies also yielded a lot of biological variability allowing the study of the relationship between different parameters. Yang et al. (2007) studied 150 recombinant inbred lines of the cross Hanxuan 10× Lumai 14 lines of wheat under drought and well-watered conditions. The authors screened the following parameters: Chl content, *F*_*O*_, *F*_*M*_, *F*_*V*_, *F*_*V*_/*F*_*M*_, *F*_*V*_/*F*_*O*_. The authors noted that for each of the two conditions studied different QTLs were found. An observation that is made in several other studies as well. Yin et al. (2010) studied 184 recombinant inbred lines of crosses of the soybean varieties Kefeng no 1 and Nannong1138-2. The authors used the following parameters for screening: TRo/ABS (*F*_*V*_/*F*_*M*_ JIP test), ETo/TRo, REo/ETo, ABS/RC, *PI*_abs_, *F*_*V*_/*F*_*M*_ (PAM), *F*_*V*_′/*F*_*M*_′, *q*_*P*_, Φ_PSII_, *P*_n_ (CO_2_ assimilation rate). The authors planted at different times in order to be able to measure all the plants at the same age. Remarkable was the observation that the *F*_*V*_/*F*_*M*_ measured with the HandPEA did not yield the same results as the *F*_*V*_/*F*_*M*_ measured with a PAM instrument. It is possible that the authors erroneously used the 50-µs point for the *F*_*O*_ value of the OJIP measurements, but it may also suggest that there were methodological problems. Šimić et al. (2014) studied 205 recombinant inbred lines of the cross of the maize lines B73 × M017 in four environmental conditions differing in soil moisture levels. The authors used the following parameters for screening: TRo/ABS, ETo/TRo, ABS/RC, TRo/RC, ETo/RC, DIo/RC, TRo/DIo, ETo/(TRo − ETo), *PI*_abs_. The authors identified 10 significant QTLs, but also observed that different QTLs were found for different environmental conditions.

With respect to the JIP test parameters chosen in the above-mentioned studies, it can be noted that most of them have not been characterized physiologically. See the next question for a discussion about the choice of parameters for this type of study.

Fracheboud et al. (2002, 2004) tried to link their QTLs to candidate genes. In most of the other cited studies the link with the physiology of the studied plants is less evident.

## Question 30: Which parameters to choose for QTL studies?

Fluorescence parameters reflect an underlying process or processes. If we would assume, for example, that a decrease in PSI content during stress is a marker for oxidative damage, then the resistance of the IP amplitude to stress could be a good candidate for a QTL study. Although the meaning of changes in the ratio between the amplitude of the photochemical phase and the amplitude of the thermal phase has not been established, it probably reflects a fundamental property of the chloroplast and may, therefore, be another candidate. Another parameter could be the normalized area [the parameter Sm of the JIP test (Strasser et al. 2004)], or even the normalized area split in parts (area between *F*_*M*_ and the OJ, JI and IP rise, respectively; see Tóth et al. 2007b and Question 16) that give information on the ratio between PSII and the number of electron acceptors downstream (PQ pool and acceptor-side PSI). For the *q*_E_ several genes have already been identified (Jung and Niyogi 2009) and it may, therefore, be a less interesting parameter for a QTL study. The parameter *q*_P_, as a rough indicator of the balance between excitation pressure and electron flow, in response to a certain stress may be another candidate parameter to screen for stress resistance genes.

Parameters that have not been characterized physiologically are less rational choices for QTL studies. Many JIP test parameters are only used conceptionally and have so far not been characterized physiologically in a proper way. Quite a few of the JIP test parameters mentioned in the previous question belong to this category of conceptional parameters. Other parameters are so fundamental that they are unlikely to change much. The quantum yield of PSII has been shown to be sensitive to antenna size (Wientjes et al. 2013a), but, apart from that, is essentially invariable (see Question 6). On the other hand, the parameter *F*_*V*_/*F*_*M*_ can be used to probe the resistance to photoinhibition.

QTL studies are, in principle, not so different from mutant screening. If you do not have a well-thought-out strategy, it is unlikely that you will obtain interesting information.

In summary, in our opinion, QTL studies can yield more interesting information if a more rational approach, making better use of our knowledge of the meaning of fluorescence parameters, is applied.

## Question 31: Can Chl *a* fluorescence measurements/parameters be used for crop improvement?

As discussed in response to several questions in this paper (e.g., 25 and 26), Chl *a* fluorescence can be used to monitor environmental stress. Therefore, fluorescence parameters can, in principle, be used as selection tools in plant breeding programs and for analyzing genotype–environment interactions (Araus et al. 1998; Kalaji and Pietkiewicz 2004). In quite a few studies this approach has been advocated (Greaves and Wilson 1987; Baker and Rosenqvist 2004; Kalaji and Pietkiewicz 2004).

There are a few points that should be considered: (1) It is important to obtain Chl *a* fluorescence-related traits showing a high correlation with yield or plant performance in addition to Chl *a* fluorescence-related traits that are specific for resistance to the stress of interest; (2) the measurements should cause only minimal perturbations in growth conditions: The shorter the measurements the better; (3) short measurements that are easy to carry out, to allow the accumulation of many measurements in a short time—plant characteristics change during growth in response to both age and environmental factors. To allow comparisons between different cultivars, varieties or crosses of all plants of interest, measurements have to be made within a short time interval. With respect to point (1), it may be noted that our knowledge of plant stress responses is often too limited to decide with certainty which trait will improve both stress resistance and plant yield. For example, in the case of photoinhibition it has been argued widely that this represents damage. More recently, however, it has been suggested that the inactivation of PSII by light (reversible damage) prevents damage to PSI (largely irreversible damage) (Grieco et al. 2012; see Question 8). Breeding for plants with PSII that is no longer inactivated by light could, therefore, have catastrophic consequences for the survival chances of such plants. Another example of such a conflict of interest is the observation that increasing the synthesis of enzymes that scavenge ROS may make the plant more resistant to abiotic stress, but, at the same time, more sensitive to biotic stress, because it weakens programmed-cell-death-based defense mechanisms against pathogens (Mittler 2002).

Thus, two Chl *a* fluorescence-related traits have to be used to screen for crop improvements: (1) processes that are closely correlated with plant performance, which can be monitored by Chl *a* fluorescence and (2) Chl *a* fluorescence-related traits that correlate with the stress of interest and are under genetic control and can be genetically manipulated.

In the field, plants often are subjected to several types of biotic or abiotic stresses during the growing season. If two or three types of stress interact, the response of the plant may differ considerably from the response to individual stresses. This complicates the analysis of data for breeding programs directed at a particular stress. It should be noted that the growth conditions of horticultural plants grown in greenhouses can, in this respect, be much better controlled.

If stress combinations are common, it is also possible to target such a combination of stresses. Rosyara et al. (2010) looked at the combination of high-temperature stress and spot blotch in wheat. They observed that the parameters *F*_*V*_/*F*_*M*_, Chl content (SPAD measurements) and the parameter canopy temperature depression (measured using infrared thermography) showed a better correlation with plant yield (e.g., thousand kernel weight) than the leaf area affected by spot blotch.

The inheritance of Chl *a* fluorescence features in different stress environments indicates typical quantitative traits determined by multiple QTLs (Fracheboud et al. 2004; Hund et al. 2005; Yang et al. 2007; Guo et al. 2008; Kalaji and Guo 2008; Kiani et al. 2008; Zhang et al. 2010; Czyczyło-Mysza et al. 2013; Anithakumari et al. 2012). Depending on the complexity of the processes associated with a trait, the number of QTLs and their effect size differ.

Although the technical quality of Chl *a* fluorescence measurements and the efficiency with which they can be carried out have increased over the years, there still remain many basic interpretation issues of which quite a few are mentioned here and in Kalaji et al. (2014a). Chl *a* fluorescence analysis is now rapid, sensitive, non-destructive and relatively cheap (Misra et al. 2001a, b, 2006; Kalaji et al. 2012a). A discussion issue remains under which conditions Chl *a* fluorescence measurements can be used for early detection of stress and which parameters are subsequently the best tools. Chl *a* fluorescence can be measured on whole tree canopies down to single cells or even chloroplasts (Malenovsky et al. 2009; Snel and Dassen 2000). It has to be noted though that for imaging applications, the time resolution is considerably lower than for point measurements and homogeneous illumination of the measured leaves or plants remains a concern for kinetic fluorescence measurements. Also, for tree crown measurements, the relatively large distance between measuring equipment and photosynthetic sample imposes considerable limitations on the Chl *a* fluorescence parameters that can be measured (Malenovsky et al. 2009).

One of the most important problems associated with the use of Chl *a* fluorescence parameters is the heterogeneity of photosynthetic samples. Different factors such as senescence (Wingler et al. 2005), injury/wounding (Quilliam et al. 2006), microbial infection (Scharte et al. 2005; Bonfig et al. 2006; Berger et al. 2007), leaf water status (Meyer and Genty 1999; Nejad et al. 2006), photosynthetic induction (Meyer and Genty 1999), spatial gradients in thylakoid differentiation (Pantaleoni et al. 2009), chilling (Hogewoning and Harbinson 2007) and ozone exposure (Leipner et al. 2001) can cause photosynthetic heterogeneity. This type of photosynthetic heterogeneity is difficult to capture with non-imaging fluorescence techniques. Recent advances in fluorescence imaging technology has turned it into an important tool for resolving spatial heterogeneity of leaf photosynthesis (Nedbal and Whitmarsh 2004; Oxborough 2004). But even when using imaging techniques, it is not easy to quantify the effect of a pathogen infection on the photosynthetic capacity of a leaf based on Chl *a* fluorescence measurements alone. In such cases, it would probably be necessary to calibrate such measurements using, for example, CO_2_ assimilation measurements that yield absolute activities.

For a breeder it is important to measure all plants in a breeding program in a short time to be able to compare the different crosses (see above). However, it is also possible to use imaging or even remote sensing methods depending on the question whether the chosen parameter(s) can be determined using such techniques. Overall, the measurement of Chl *a* fluorescence can be helpful for breeding purposes if the questions “what to measure” and “how to measure it efficiently” can be resolved.

## Question 32: Are machine learning methods useful for the analysis of Chl *a* fluorescence induction curves?

Prompt fluorescence (PF) induction curves reflect energy and electron transfer processes in thylakoid membranes following a dark-to-light transition of dark-adapted photosynthetic samples. Analyzing the shapes of the induction transient, the absolute values of the fluorescence signal at different times F_*t*_ during induction (OJIP), their relative amplitudes, normalized to the variable part of the signal, or difference curves reflecting stress effects, we can study different sites of the photosynthetic electron transport chain and monitor stress effects on them. Stress often affects not only specific sites of the photosynthetic structure or specific reactions, but modifies a wide range of cell structures (which may not all be directly related to the photosynthetic apparatus) and change their function. This may have secondary (indirect) effects on photosynthetic processes. These effects can be detected looking at changes in the “fingerprint”—a term also used by Tyystjärvi et al. (1999)—formed by the constellation of parameters derived from OJIP transients or by studying the modified shape of the fluorescence rise kinetics. This “stress response” often contains hidden information concerning the stress type, a specific plant tolerance to the applied stress, and other important and interesting information related to the plant as whole and that, at first sight, is not directly connected to the photosynthetic apparatus of the plant.

The identification of such kinds of hidden information is possible through additional secondary fluorescence data processing, using methods of artificial intelligence that allow the analysis of large data sets, the amount, precision and complexity of which cannot not be efficiently analyzed by traditional methods (Samborska et al. 2014). Here, the analysis of OJIP transients is discussed. In an earlier series of articles, Tyystjärvi and coworkers tried to apply similar artificial intelligence methods to the analysis of fluorescence data induced by a sequence of different types of illumination (low light intensity, saturating pulse, far-red, etc.) in order to identify plant species (Tyystjärvi et al. 1999; Keränen et al. 2003; Codrea et al. 2003). Simulating OJIP transients is another approach to mine the information contained in OJIP transients, which is not further discussed here (reviewed by Lazár and Schansker 2009).

Machine learning methods, like artificial neural networks (ANNs) or self-organizing maps (SOM), are powerful tools of Chl *a* fluorescence data analysis. They enable us to quickly *classify* the different responses of plants to environmental stress by (1) *finding different shapes* of Chl *a* fluorescence induction curves, (2) determine the most important Chl *a* fluorescence parameters or points on the Kautsky curve that differentiate them. Moreover, we can also (3) *predict the values* of other environmental or physiological variables on the basis of Chl *a* fluorescence data (Goltsev et al. 2012).

### Reducing data complexity

For an analysis, we only need the table of points of Chl *a* fluorescence induction curves, obtained from fluorimeters, or the table of measured/calculated Chl *a* fluorescence parameters. Chl *a* fluorescence data usually show noisy patterns of many variables and parameters cluttered on the charts. By using multivariate analyses similar to principal component analyses (PCAs) we can reduce the large set of Chl *a* fluorescence variables to the few most informative ones (Legendre and Legendre 2012; Goltsev et al. 2012). This way, we can also detect the main *trade*-*offs* among Chl *a* fluorescence parameters and remove errors from the data set.

### Relating Chl a fluorescence parameters to environmental or physiological processes

Artificial neural networks are designed to perform analyses that relate the selected Chl *a* fluorescence parameters (input) and output variables through a network of interconnected cells called neurons in a learning process in the same way the human brain works (Goltsev et al. 2012). The typical objective of ANNs is to find the Chl *a* fluorescence parameters that characterize the “stressed” or “unstressed” plants (Kalaji et al. 2014b) or to enable us to predict values [e.g., water content in plant tissue (Goltsev et al. 2012), or predict the plant biomass].

### Visualization and classification

Self-organizing maps (SOM) are used as a data mining and visualization method for complex data sets. SOM is a type of neural network and can be seen as a nonlinear form of PCA. This method can also be used for the classification of fluorescence induction curves measured with portable fluorimeters by displaying the groups of Chl *a* fluorescence parameters in an easy to understand form in a two-dimensional plane (Maldonado-Rodriguez et al. 2003).

#### *Example 1*

**Fig. 11:**
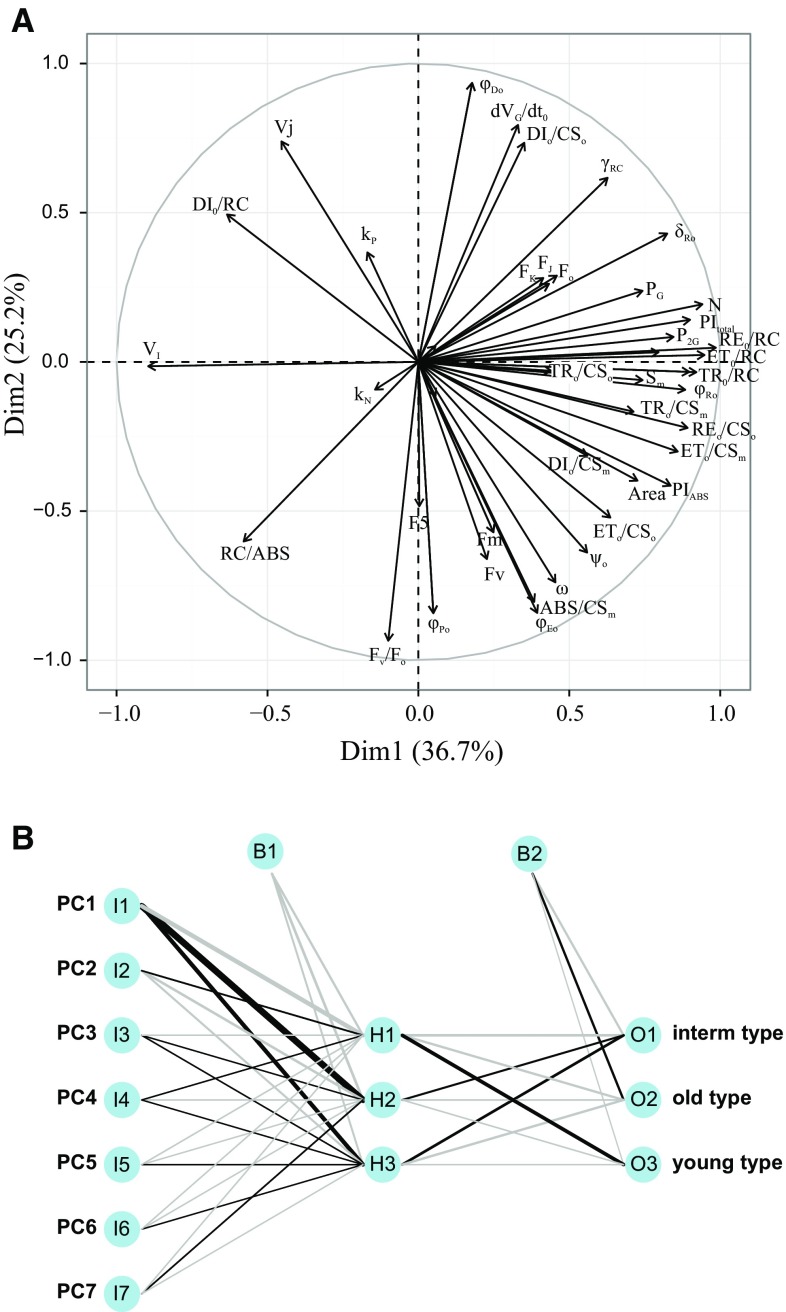
Machine learning methods. **a** principal component analysis (PCA) of 54 Chl *a* fluorescence parameters derived from a study on the effect of the density of grass on OJIP measurements. **b** Artificial neural network (ANN) analysis of the same data set. For further details see Question 32 (Bąba, unpublished data)

In Fig. 11a, the results of a PCA analysis of 54 Chl *a* fluorescence parameters randomly sampled from a large set of Chl *a* fluorescence measurements measured on *Brachypodium pinnatum* (a grass) are shown. The grass grew in meadows, and its density increased with age. The relative variable fluorescence at the *I* step (*V*_I_) decreases, when we move along the first PCA axis (Dim1), from the left to the right side of Fig. 11a. At the same time an increase of the parameters that, according to the JIP test, represent specific energy fluxes expressed per active PSII reaction center (TR_*O*_/RC, ET_*O*_/RC, RE_*O*_/RC) is observed. When we move along the second PCA axis (Dim2), from bottom to upper side of Fig. 11a, the increase of the values of the parameters *F*_V_/*F*_*O*_ and maximum quantum yield of primary PSII photochemistry (**ϕ**_Po_) can be seen. In this way we find the four most important parameters (instead of the initial 54), which describe the state of the photosynthetic apparatus, related to the studied increase in the stand density of grass.

#### *Example 2*

In Fig. 11b, the artificial neural network (ANN) analysis of Chl *a* fluorescence of the same data set is shown. As in the previous analyses we performed the PCA and retained the first 7 axes (the two most important axes are related to fluorescence parameters from *Example 1*). The ANNs find the relationships between these parameters and the plants of different age growing in grasslands. In other words, the analyses allow the comparison of the responses of the photosynthetic apparatus to the condition of these three plots.

#### *Example 3*

**Fig. 12:**
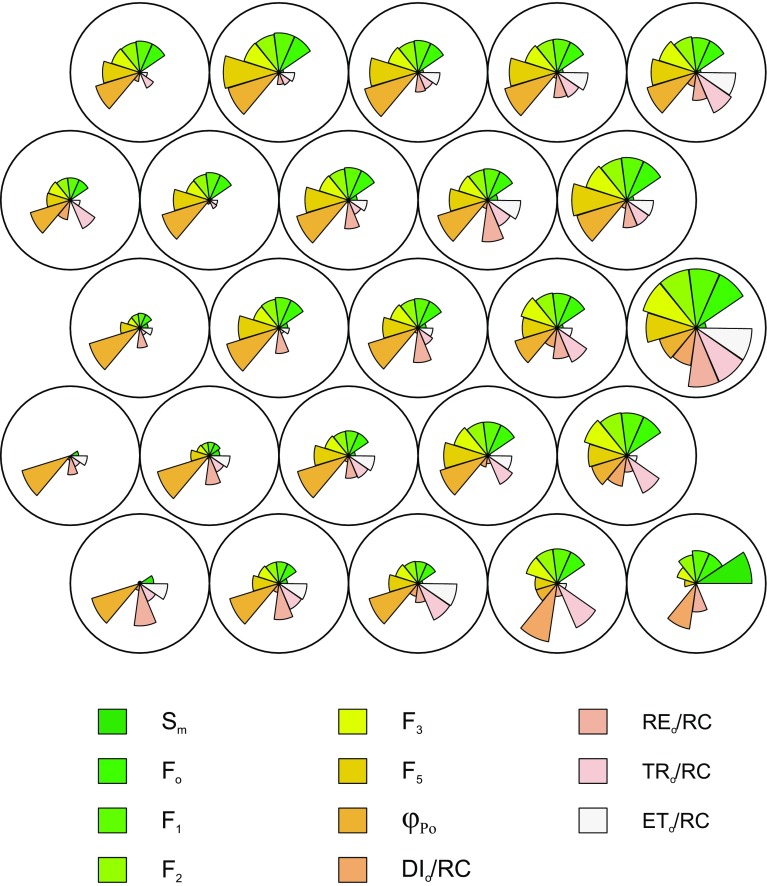
Example of a self-organizing map (SOM) based on a set of OJIP measurements of unstressed and drought-stressed plants. For further details see Question 32 (Bąba, unpublished data)

Unsupervised self-organizing maps (SOM) using another population of plants growing under stressed (drought stress) and unstressed (well-watered) conditions. The pattern of differences in Chl *a* fluorescence parameters (*F*_0_, *F*_1_, TR_0_/RC, ET_0_/RC, RE_0_/RC, Sm, **ϕ**_Po_) is easily detectable (SOM; Fig. 12).

Examples of successful use of machine learning methods for Chl *a* fluorescence analysis from the literature are:

1. Taxonomic classification of plant species based on Chl *a* fluorescence parameter data using an artificial neural network (Kirova et al. 2009)
2. Determination of relative water contents (RWCs) in leaves based on PF, DF and MR820 signals (=820 nm reflection signal) or calculated JIP parameters (Goltsev et al. 2012)
3. Prediction of the yield of wheat based on selected factors in wheat crop production with self-organizing maps (Pantazi et al. 2014)

How to perform the analyses? All analyses presented above are implemented using statistical packages, for example Statistica (StatSoft Inc. 2011) or SAS (SAS Enterprise Miner; SAS Institute, Cary, NC). Neural network analyses can also be performed in MATLAB with the Neural Network Toolbox (MathWorks).

## Question 33: Which are the most useful books about Chl *a* fluorescence?

There are not many books dealing with Chl *a* fluorescence. Since the 1980s there have been two classic references: “Light Emission by Plants and Bacteria” (1986), edited by Govindjee, Amesz and Fork; and “Applications of Chlorophyll Fluorescence in Photosynthesis Research, Stress Physiology, Hydrobiology and Remote Sensing” (1988), edited by Lichtenthaler. More recently, Laisk and Oja (1998) published “Dynamics of Leaf Photosynthesis on Dynamic Measurements of Photosynthesis in Leaves” and DeEll and Toivonen (2003) published “Practical Applications of Chlorophyll Fluorescence Science in Plant Biology.” Further there are three books in the series Advances in Photosynthesis and Respiration that contain much information on Chl *a* fluorescence: “Chlorophyll *a* Fluorescence: a Signature of Photosynthesis” (2004), edited by Papageorgiou and Govindjee, “Photosynthesis in Silico: Understanding Complexity from Molecules to Ecosystems” (2009), edited by Laisk, Nedbal and Govindjee and “Non-photochemical Quenching and Energy Dissipation in Plants, Algae and Cyanobacteria” (2014), edited by Demmig-Adams, Garab, Adams and Govindjee. A recent addition is “Applications of Chlorophyll Fluorescence in Understanding Plant Performance” (2016), edited by Kalaji et al. In addition, several review papers have been published: Krause and Weis (1991), Dau (1994), Govindjee (1995), Rohácek and Barták (1999), Maxwell and Johnson (2000), Roháček (2002), Lazár (2006), Logan et al. (2007), Baker (2008), Henriques (2009), Allakhverdiev (2011), Gorbe and Calatayud (2012), Misra et al. (2012), Kalaji et al. (2012b), Murchie and Lawson (2013) and Lazár (2015).

## Concluding remarks

Chlorophyll *a* fluorescence is a simple, non-invasive, non-destructive, quick and relatively easy way to determine the efficiency and activity of the photosynthetic electron transport chain. However, as this paper and the previous paper (Kalaji et al. 2014a) demonstrate, the interpretation of fluorescence measurements demands knowledge. Both knowledge of the sample (e.g., do I still measure the true *F*_*O*_ or true *F*_*M*_) but also of all the photosynthetic processes that can affect the fluorescence intensity, are important. Once these two requirements are met, Chl *a* fluorescence offers, without doubt, a very broad range of probes for the monitoring of processes related to photosynthesis.

The parameters derived from fluorescence signals can provide information about the structure (PSII antenna size, photosystem integrity and stoichiometry of components of the ETC) and function of PSII and the ETC. It has to be emphasized once more that the utility Chl *a* fluorescence is not limited to PSII. Chl *a* fluorescence measurements can also give insight into the function and content of PSI, cyclic electron flow and excitation transfer to and between the photosystems. Several fluorescence methods (e.g., prompt and delayed Chl *a* fluorescence), measured using various protocols and instruments, are applied at different temperatures to decipher the functional integrity of the pigment protein complexes in different photosystems. The impact of biotic and abiotic environmental stress on green plants—but also on algae and cyanobacteria—and in many cases their early detection is possible. Fluorescence methods can also be combined with other photosynthetic techniques to help decipher the intricate regulation and complex interactions between a plant’s metabolic systems.

Thanks to the efforts of several generations of scientists in the field of photosynthetic research, Chl *a* fluorescence is becoming more and more one of the success stories in plant physiology. Over the last decades the number of users of this technique in basic and applied research has grown exponentially. However, if new users lack sufficient basic knowledge of Chl *a* fluorescence and of the experimental possibilities of Chl *a* fluorescence-based techniques, there is a high risk that the users will misinterpret or overinterpret their results, that they will insufficiently take into account the preconditions that have to be met to successfully apply Chl *a* fluorescence and that they will not make use of all the possibilities the method offers to them.

In this review we have treated an additional set of questions relevant to contemporary Chl *a* fluorescence research. Nevertheless, many details and specific aspects of Chl *a* fluorescence are beyond the scope of this review and every reader is urged to consult the primary literature for a fuller treatment of this rapidly moving area of research. Finally, as many of the treated questions imply, much is still unknown, many topics rest on weak foundations, new protocols can be designed, and new instruments developed. There is still considerable potential for chlorophyll fluorescence techniques to be improved and to expand, providing new insights into the fundamental process of photosynthesis.

## Abbreviations

ANN: Artificial neural network
Area, Sm: Complementary area above the fluorescence rise and this area normalized to *F*_*V*_, respectively
ATP: Adenosine triphosphate
Car: Carotenoid
Chl: Chlorophyll
^1^Chl, ^3^Chl: Singlet chlorophyll and triplet chlorophyll
Chl_D1_: Accessory Chl molecule bound to the D1 protein
CP43, CP47: Core antenna proteins of PSII of 43 and 47 kDa, respectively
CSm: Cross section (in the JIP test it is assumed that *F*_*M*_ is a measure for the cross section)
cyt: Cytochrome
D1 protein: One of the major PSII reaction center proteins, the other being D2
DCMU: 3-(3,4-Dichlorophenyl)-1,1-dimethylurea
ETC: Electron transport chain
ETR: Electron transport rate
Fd: Ferredoxin
FNR: Ferredoxin NADP^+^ reductase
*F*_*O*_, *F*_*M*_, *F*_*O*_′, *F*_*M*_′: Minimum and maximum fluorescence intensity emitted by dark- and light-acclimated samples, respectively
*F*_PSI_: Chlorophyll *a* fluorescence emitted by photosystem I
*F*_S_: Steady-state chlorophyll *a* fluorescence
*F*_*V*_/*F*_*M*_: Maximum quantum yield of primary photosystem II photochemistry
IRGA: Infrared gas analyzer
JIP test: Analysis framework for the interpretation of OJIP transients developed by Bruno and Reto Strasser
K step: Fluorescence intensity at 300 µs
kF, kN and kP: Rate constants for Chl *a* fluorescence, heat dissipation and photochemistry
LED: Light-emitting diode
LHC, LHCI and LHCII: Light-harvesting complex, in general, associated with PSI and mainly associated with PSII, respectively
M_*o*_: The initial slope (first 250 µs) of the OJIP transient times 4, normalized to *F*_*V*_
NADP^+^: Nicotinamide adenine dinucleotide phosphate, oxidized form
NO: Nitric oxide
NPQ, *q*_*N*_: Non-photochemical quenching expressed as (*F*_*M*_/*F*_*M*_′ − 1) and (1 − *F*_*V*_′/*F*_*V*_), respectively
OEC: Oxygen-evolving complex
OJIP: Fluorescence rise on a dark-to-light transition from a minimum value *O* via the intermediate steps *J* and *I* to the maximum value *P*, which is *F*_*M*_ if the light is saturating
P680, P700: PSII and PSI reaction center chlorophyll dimer, respectively
PAR: Photosynthetically active radiance
PCA: Principal component analysis
PF, DF: Prompt fluorescence and delayed fluorescence, respectively
Pheo: Pheophytin, cofactor bound to PSII
*P*I_abs_, *P*I_tot_: Performance indexes of the JIP test
*P*_n_, *I*_PL_: Net rate of carbon fixation and model based calculated net rate of carbon fixation, respectively
PPFD: Photosynthetic photon flux density
PsbO, PsbP and PsbQ: PSII extrinsic proteins
PSI, PSII: Photosystems I and II, respectively
*Q* cycle: Cyclic electron transport through cyt b6f and the PQ pool
*Q*_*A*_, *Q*_*B*_, PQ: Primary and secondary quinone electron acceptors of PSII and free plastoquinone, respectively
*q*_E_: Energy quenching, fluorescence quenching dependent on an acidification of the lumen
*q*_P_, *q*_L_: Photochemical quenching calculated based on the puddle and lake model, respectively
QTL: Quantitative trait locus
*q*_*Z*_: Non-photochemical quenching of Chl *a* fluorescence related to the xanthophyll cycle
RC: Reaction center
*R*_Fd_: Relative fluorescence decrease ratio
RLC: Rapid light curve
ROS: Reactive oxygen species
Rubisco: Ribulose-1,5-bisphosphate carboxylase/oxygenase
RWC: Relative water content
SOM: Self-organizing map
SPAD: Refers to an instrument used to estimate the leaf Chl content
*S* states *S*0, *S*1, *S*2, *S*3 and *S*4: Redox states of the oxygen-evolving complex
$t_{{F_{m} }}$: Time needed to rise from *O* to *P*
Tl: Leaf temperature
TL: Thermoluminescence
TyrD, TyrZ: Tyrosine D and Z, redox active tyrosines in the D2 and D1 proteins of PSII, respectively
UV: Ultraviolet
V, A, Z: Violaxanthin, antheraxanthin and zeaxanthin, respectively
VDE: Violaxanthin de-epoxidase
*V*_*J*_, *V*_*I*_: Relative position of the *J* and *I* steps between *O* and *P*
Φ_P0_: Maximum quantum yield of primary photochemistry
Φ_PSI_, Φ_PSII_: PSI and PSII operating efficiency, respectively
ψ*E*_*o*_: JIP test parameter thought to be related to forward electron transport, defined as 1 − *V*_*J*_
